# Supplementary material for: Phenothiazine- and Carbazole-Cyanochalcones as Dual Inhibitors of Tubulin Polymerization and Human Farnesyltransferase
Source: Pharmaceuticals (Basel). 2023 Jun 16;16(6):888. doi: 10.3390/ph16060888 (PMC10305003; doi:10.3390/ph16060888)

Phenothiazine- and carbazole-cyanochalcones as dual inhibitors of tubulin polymerization and human farnesyltransferase

Andreea Zubaș [1], Alina Ghinet [1,2,3,*], Amaury Farce [4], Joëlle Dubois [5] and Elena Bîcu [1,*]

[1.] *'Alexandru Ioan Cuza' University of Iasi, Faculty of Chemistry, Bd. Carol I, nr. 11, 700506 Iasi, Romania.*
[2] *Junia, Health and Environment, Laboratory of Sustainable Chemistry and Health, F-59000 Lille, France.*
[3] *Univ. Lille, Inserm, CHU Lille, Institut Pasteur Lille, U1167 - RID-AGE - Facteurs de risque et déterminants moléculaires des maladies liées au vieillissement, F-59000 Lille, France.*
[4] *Univ. Lille, Inserm, CHU Lille, U1286 – Infinite-Institute for Translational Research in Inflammation, F-59000 Lille, France.*
[5] *Institut de Chimie des Substances Naturelles, UPR2301, CNRS, Centre de Recherche de Gif, 91190 Gif-sur-Yvette Cedex, France*

* Correspondence: Corresponding authors. alina.ghinet@junia.com; elena@uaic.ro

Supplementary information

^{1}H, ^{13}C NMR, 2D NMR and IR spectra of compounds 2

One-dose full graphs obtained on NCI-60 cancer cell lines panel 81

Figure S1. Results of the *in vitro* human cancer cell growth inhibition for selected compounds **2k**, **2l** and **2o**. 84

Figure S2. Docking of all dual FTIs/MTIs identified in this study on farnesyltransferase and colchicine binding sites 85

Spectra of compounds

^{1}H NMR (400 MHz, $CDCl_3$)-**1a**

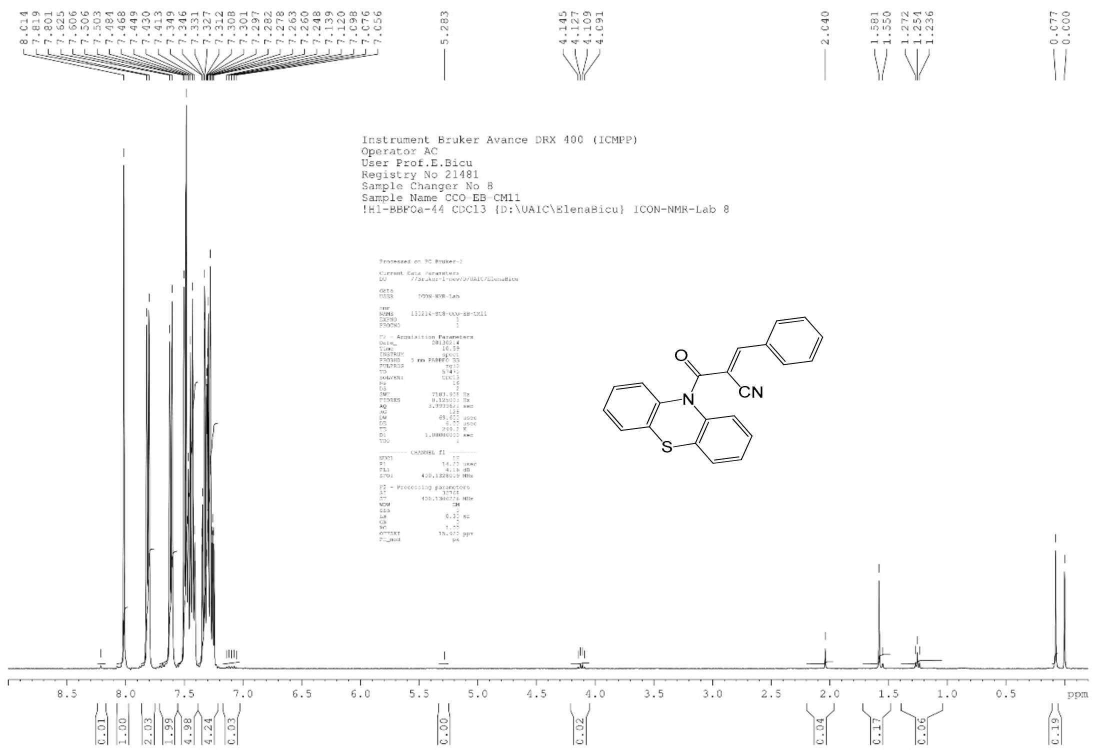

^{13}C NMR (100 MHz, $CDCl_3$)-**1a**

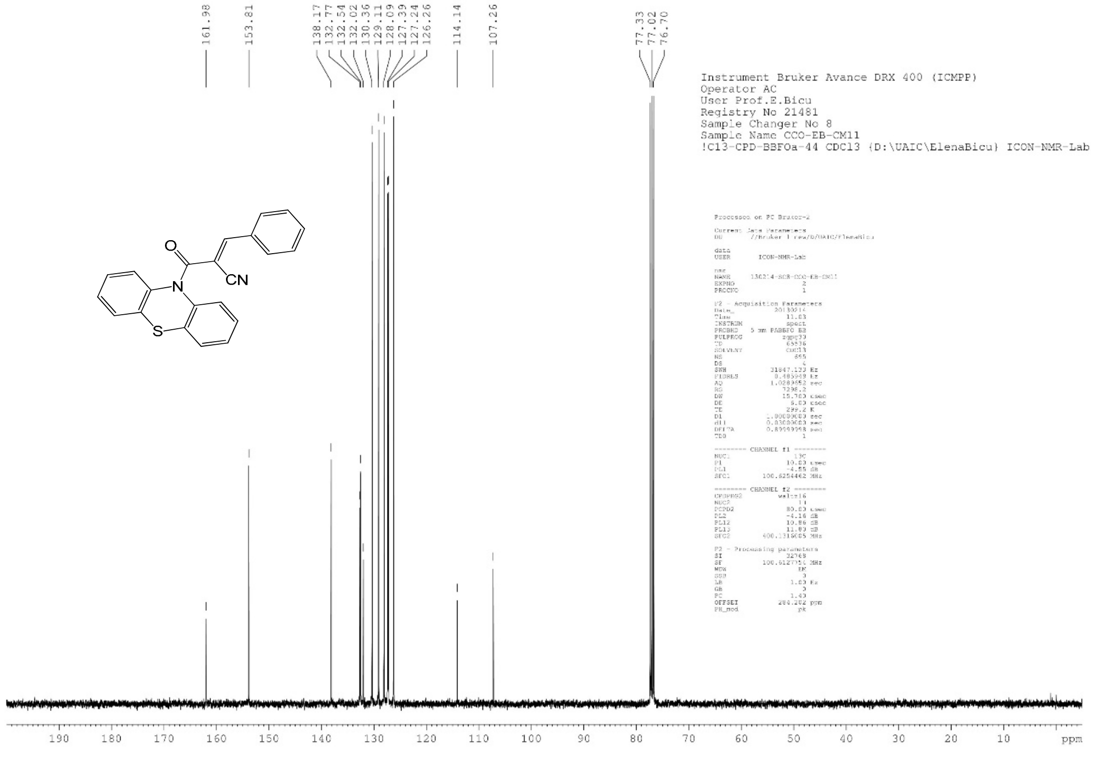

IR-1a

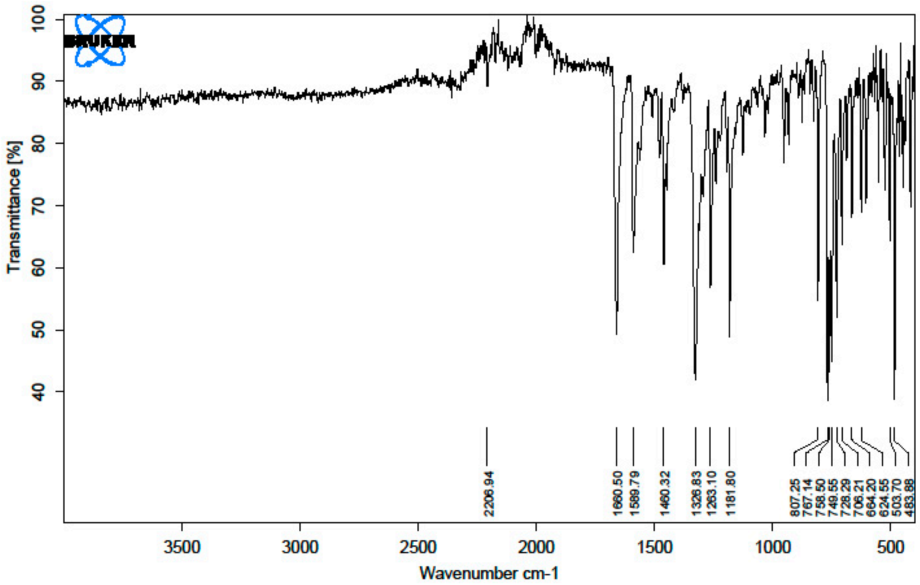

^{1}**H NMR** (500 MHz, $CDCl_3$)-**1b**

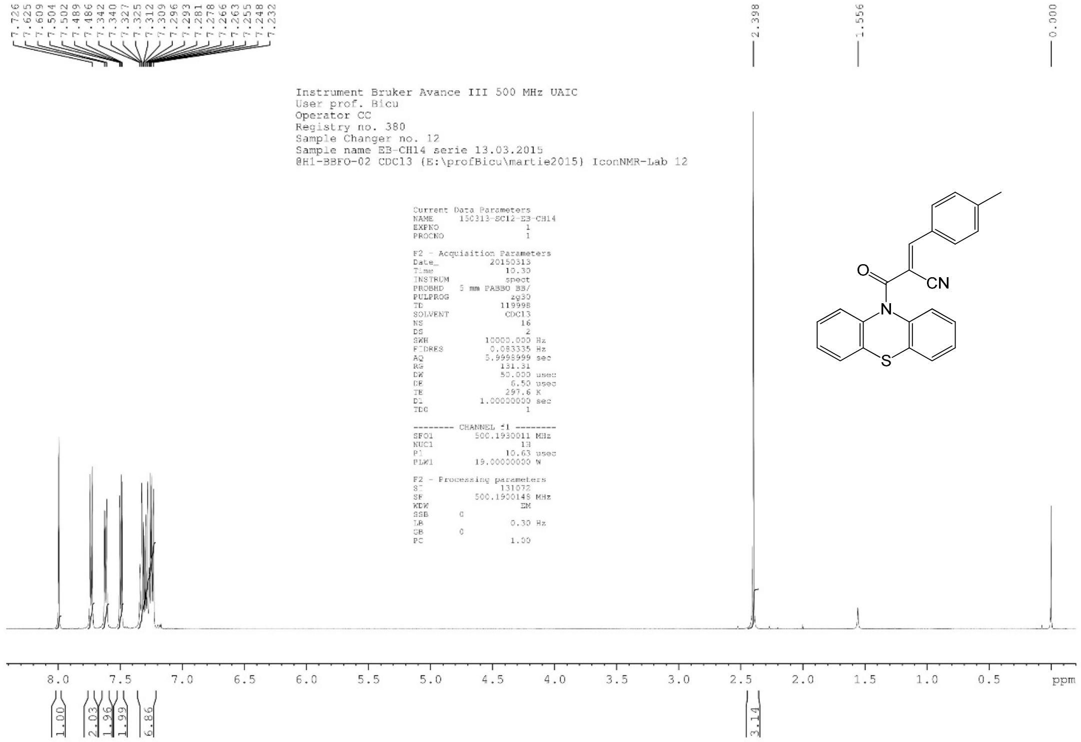

^{13}C NMR (125 MHz, $CDCl_3$)-**1b**

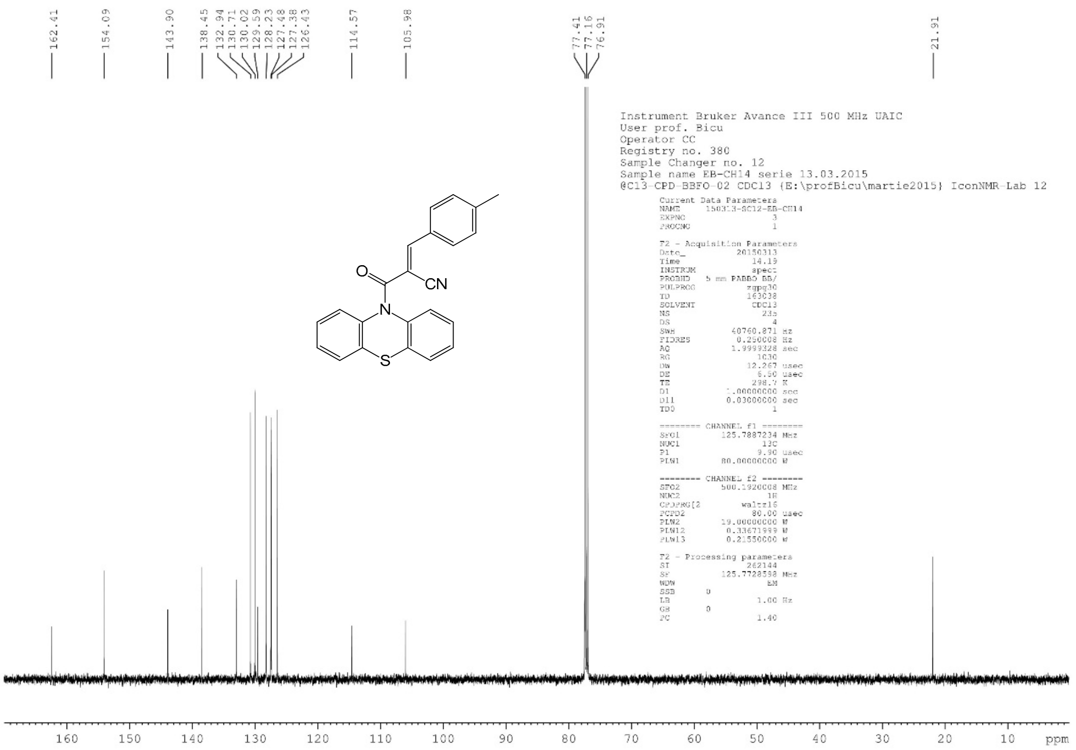

IR-1b

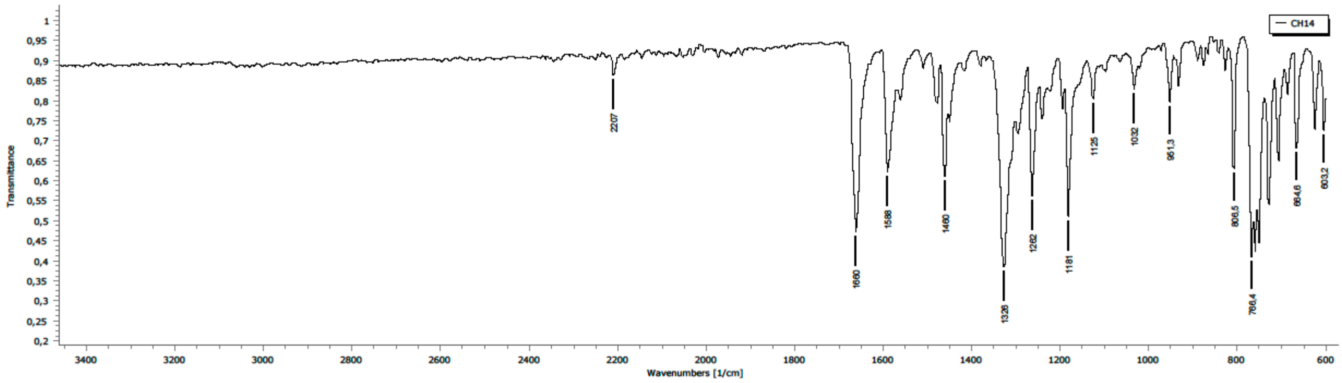

^{1}H NMR (500 MHz, $CDCl_3$)-**1c**

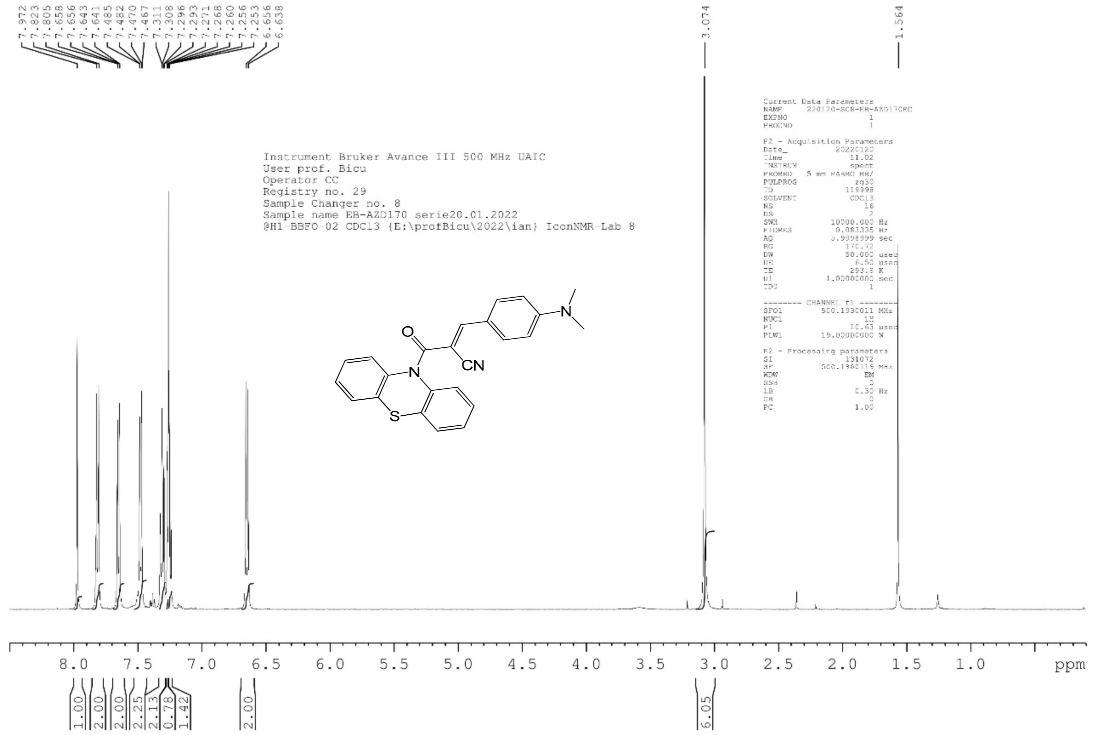

^{13}C NMR (125 MHz, $CDCl_3$)-**1c**

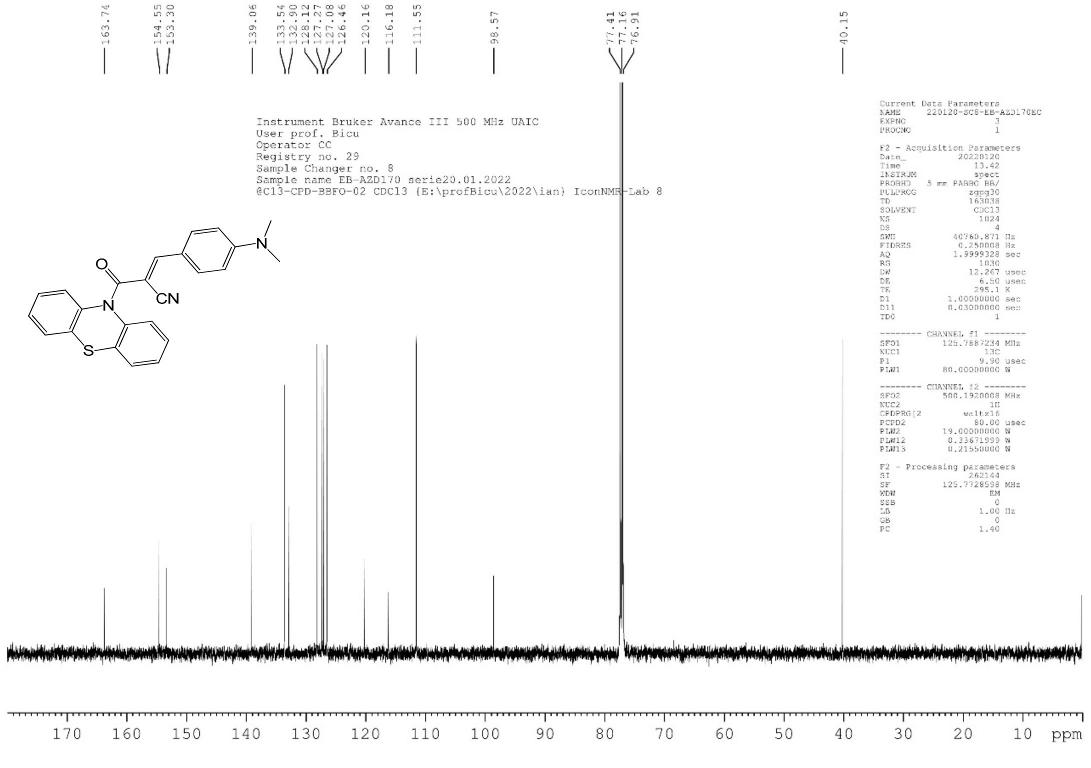

IR-1c

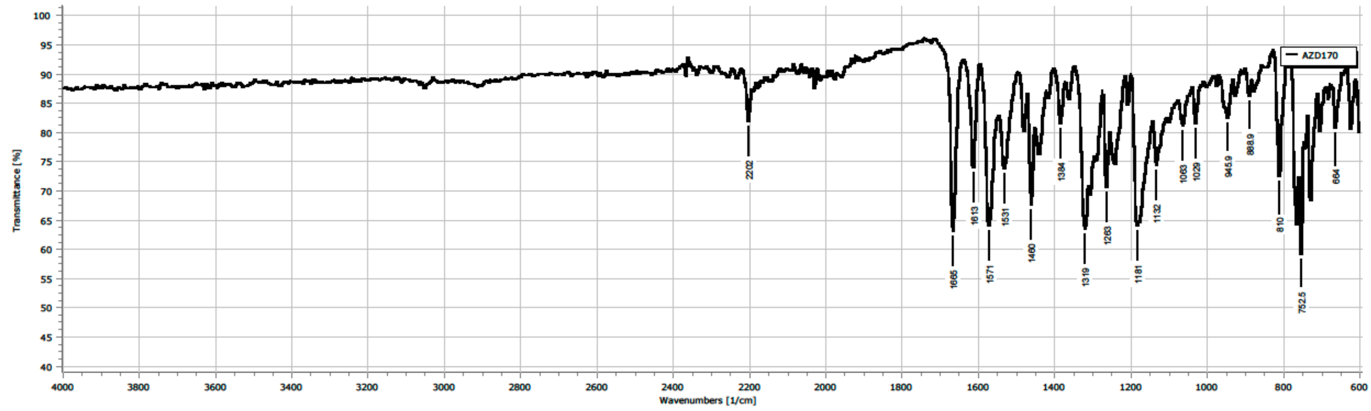

^{1}**H NMR** (500 MHz, $CDCl_3$)-**1d**

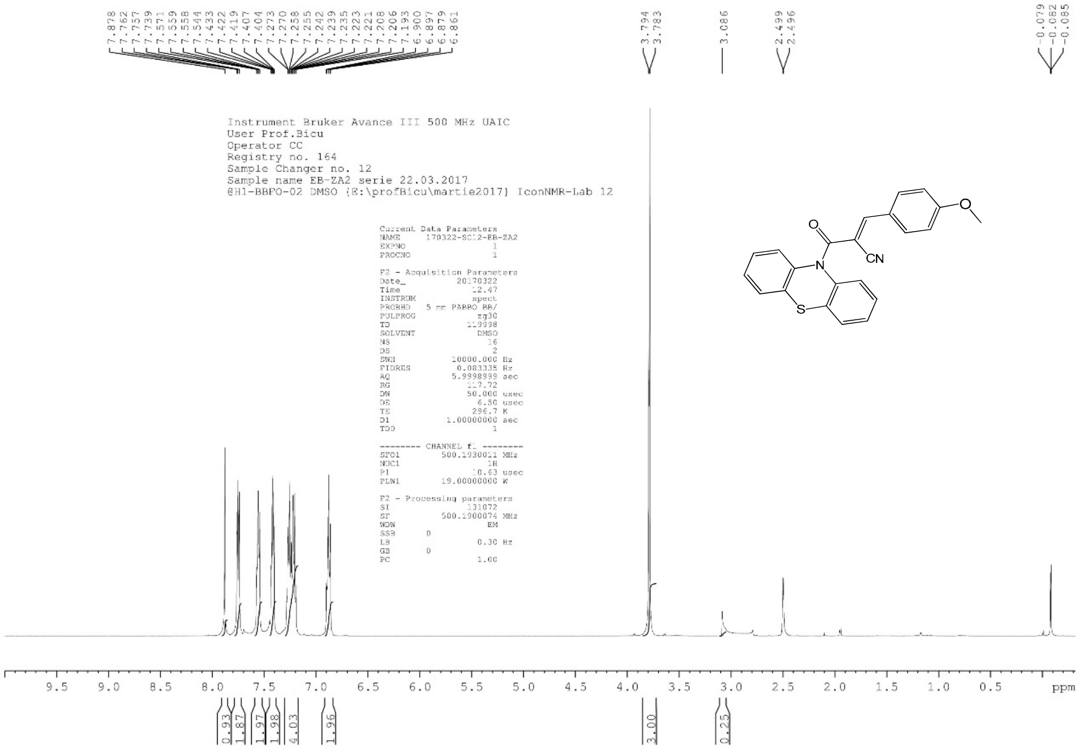

^{13}C NMR (125 MHz, $CDCl_3$)-**1d**

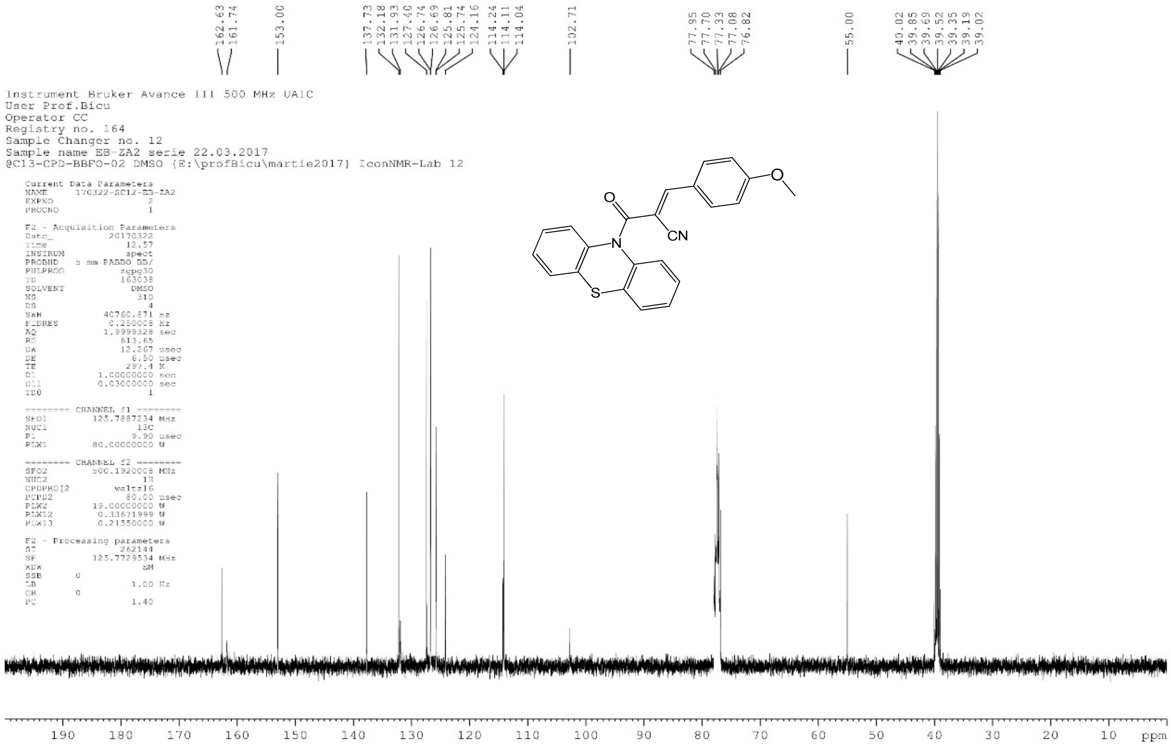

IR-1d

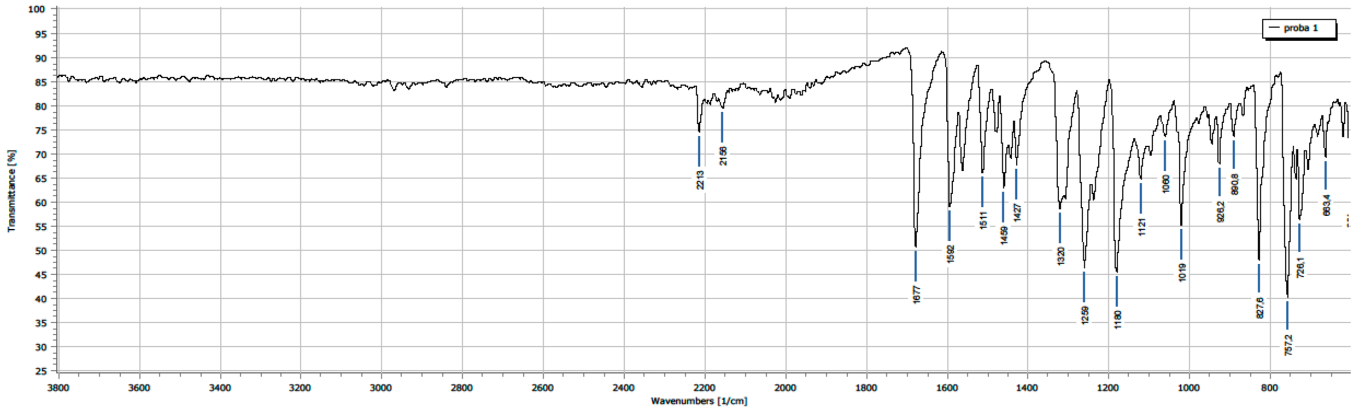

^{1}H NMR (500 MHz, $CDCl_3$)-**1e**

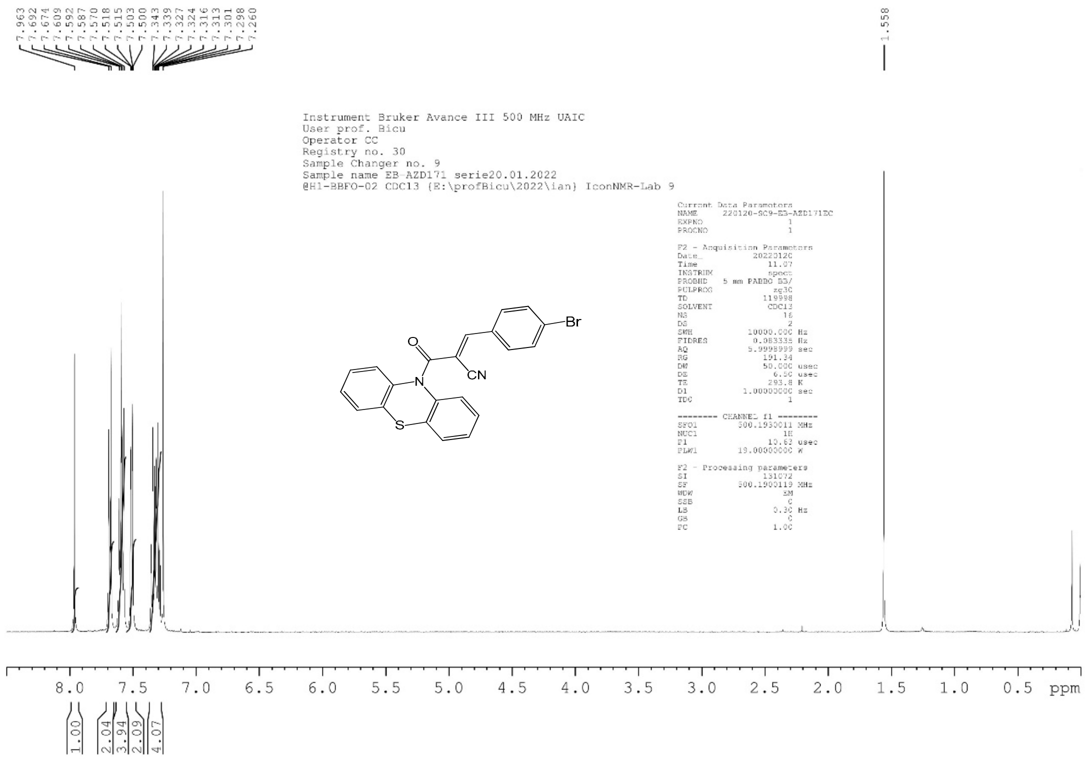

^{13}C NMR (125 MHz, $CDCl_3$)-**1e**

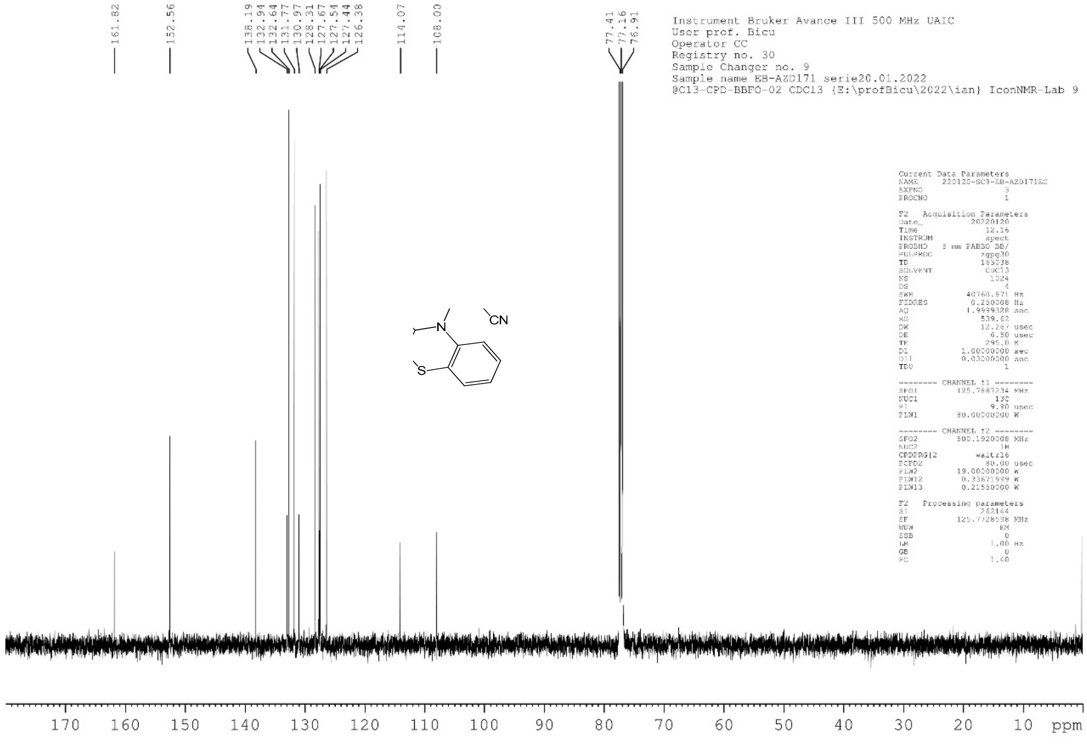

IR-1e

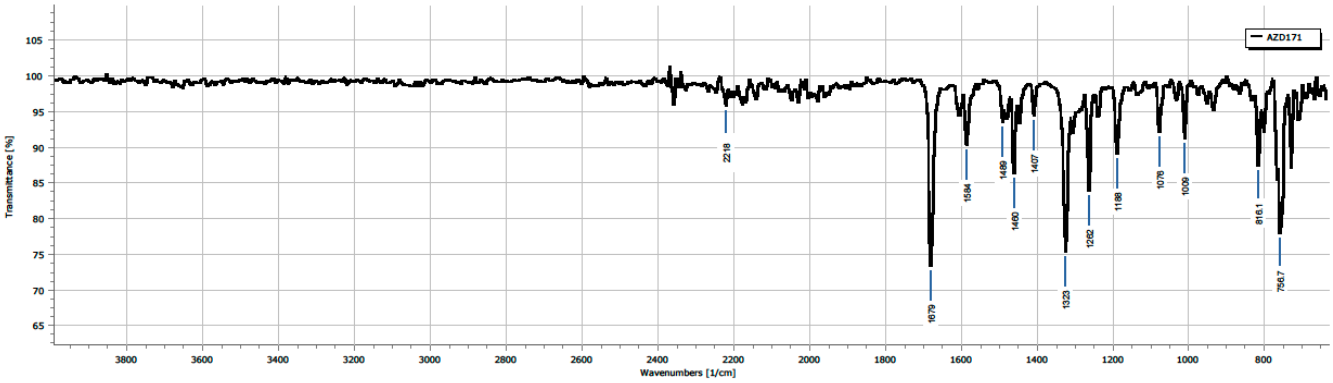
AZD171
Transmittance [%]
Wavenumbers [1/cm]
2218
1679
1584
1489
1460
1407
1323
1262
1188
1076
1009
816.1
756.7

^{1}H NMR (400 MHz, $CDCl_3$)-**1f**

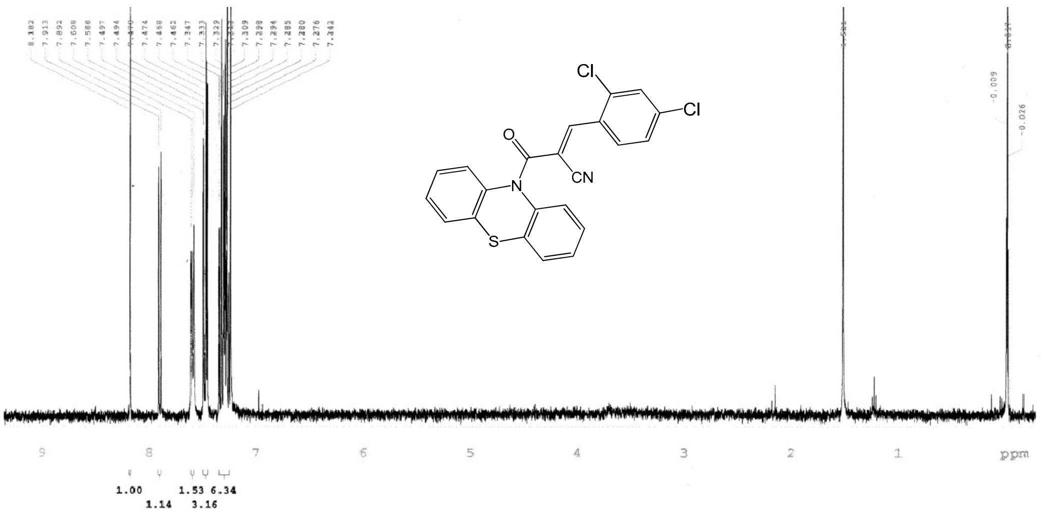

^{13}C NMR (100 MHz, $CDCl_3$)-**1f**

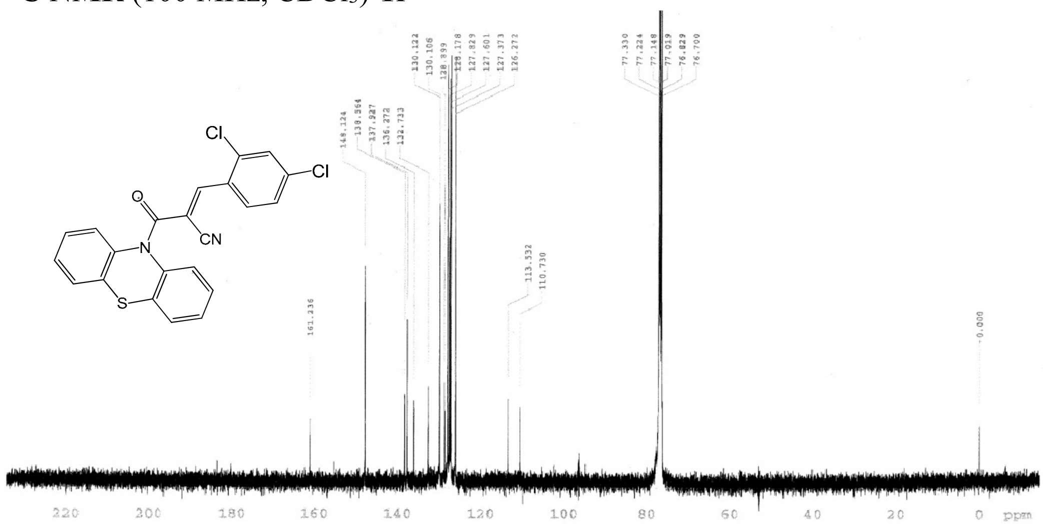

IR-1f

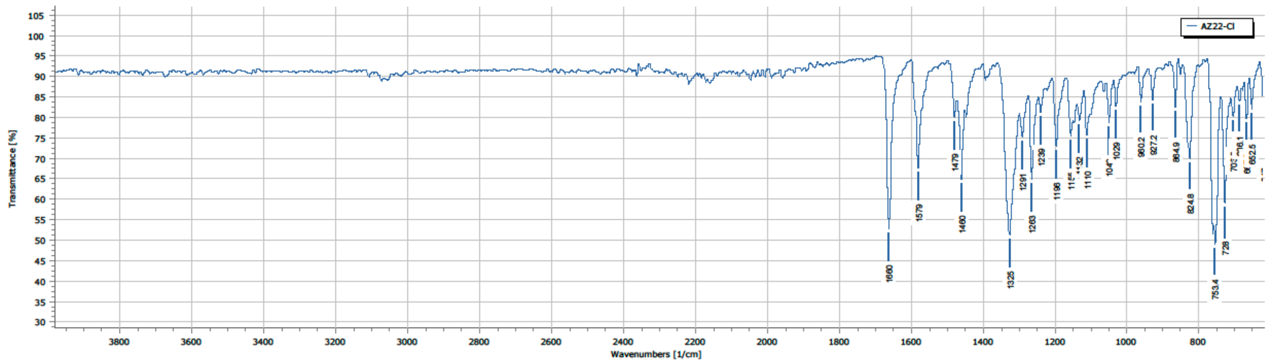

^{1}H NMR (500 MHz, $CDCl_3$)-**1g**

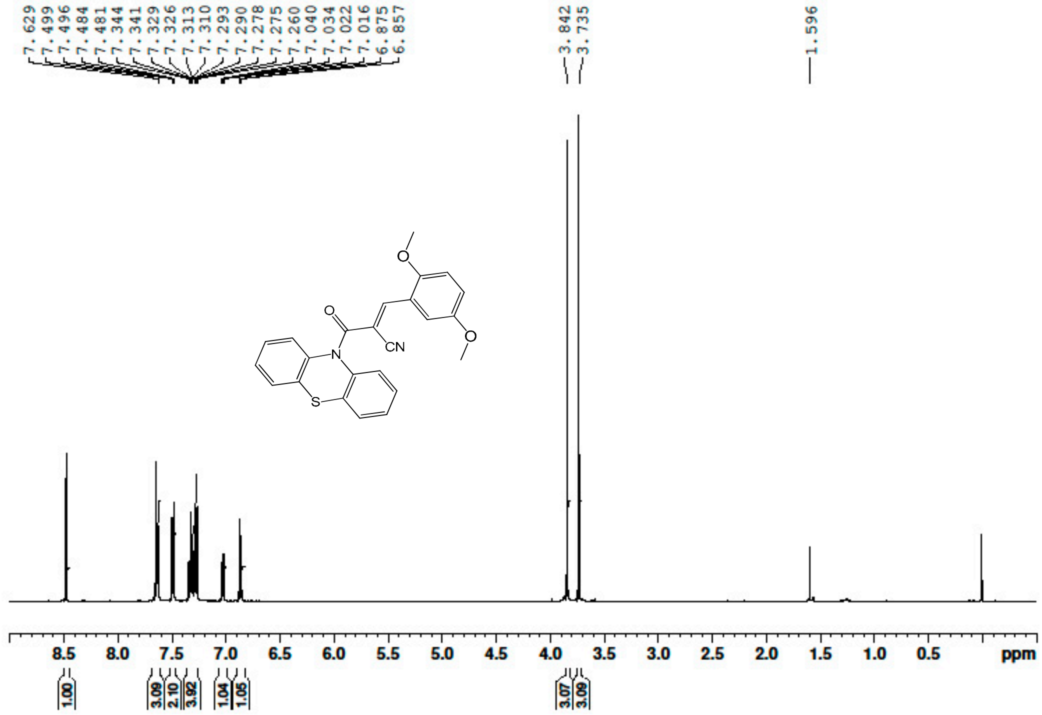

^{13}C NMR (125 MHz, $CDCl_3$)-**1g**

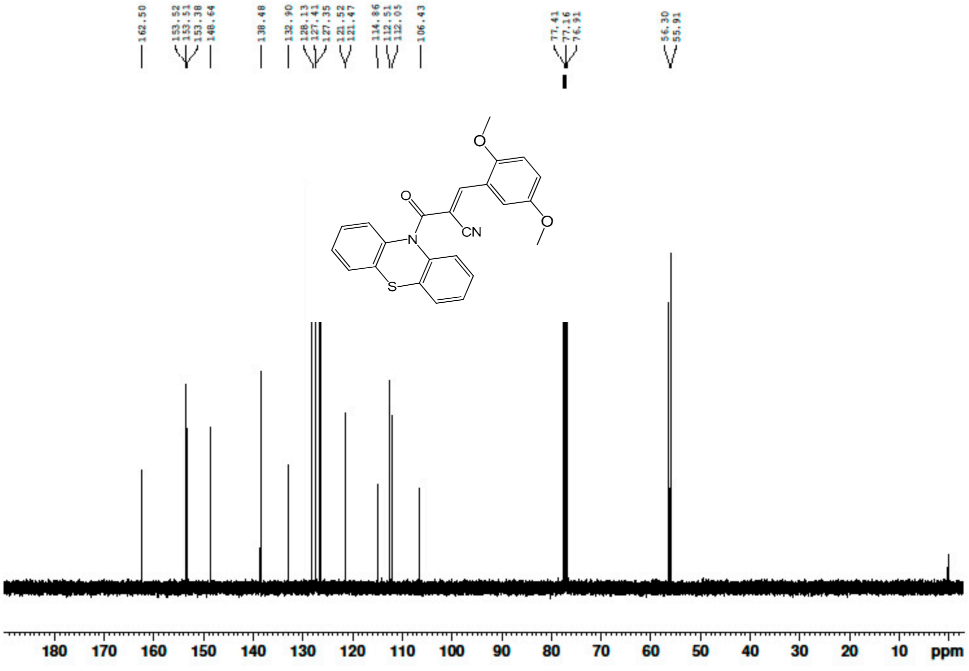

^{13}C-DEPT NMR (125 MHz, $CDCl_3$)-**1g**

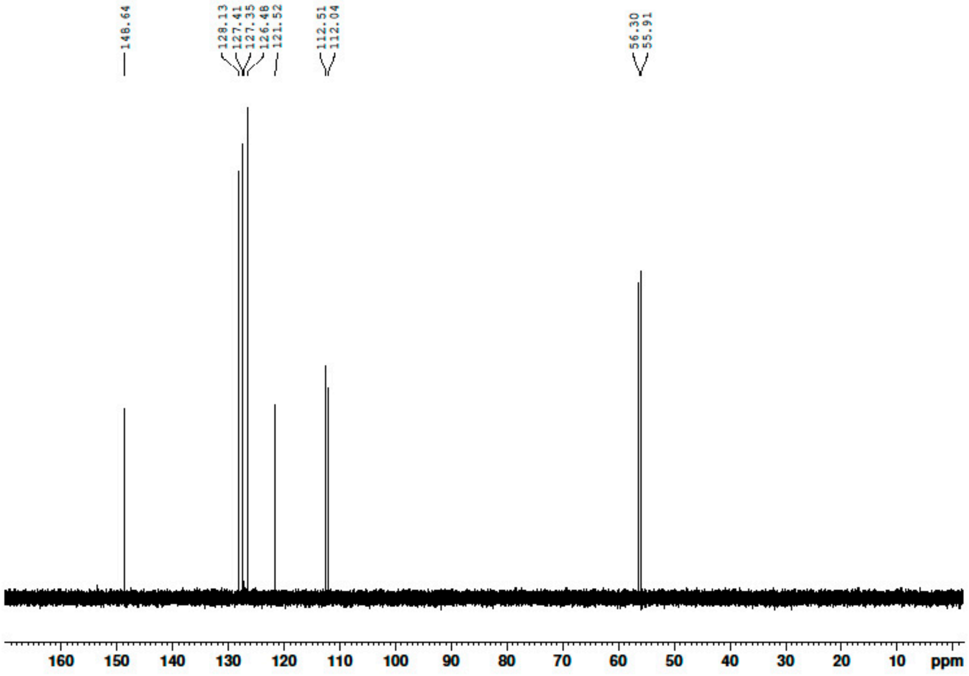

HMQC NMR (500 MHz, $CDCl_3$)-**1g**

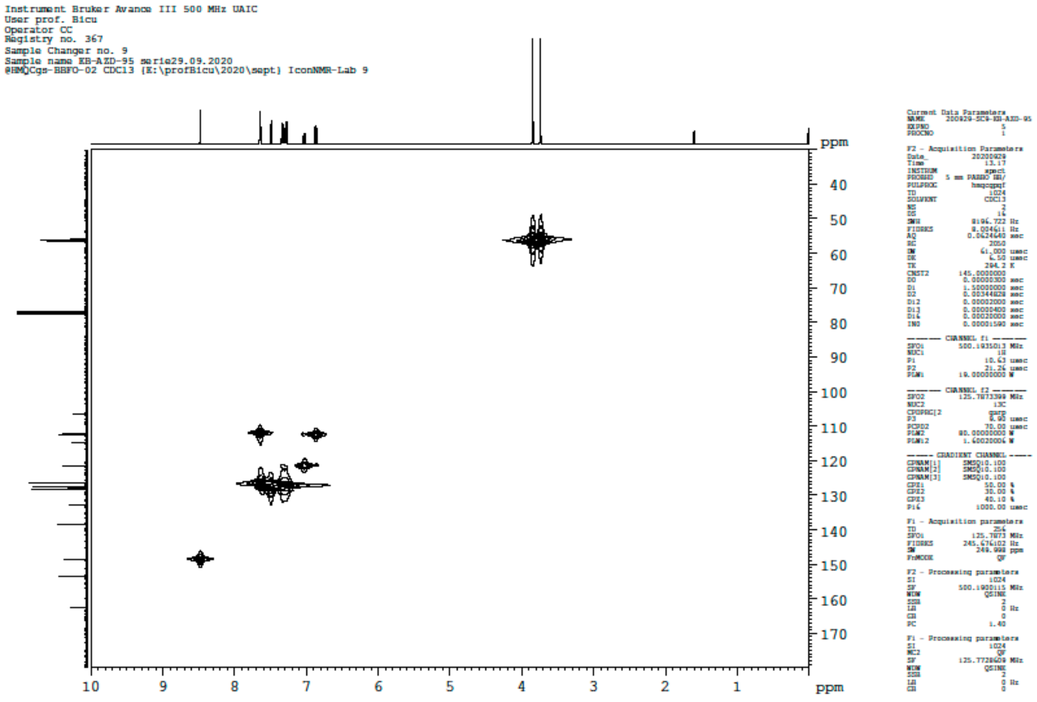

IR-1g

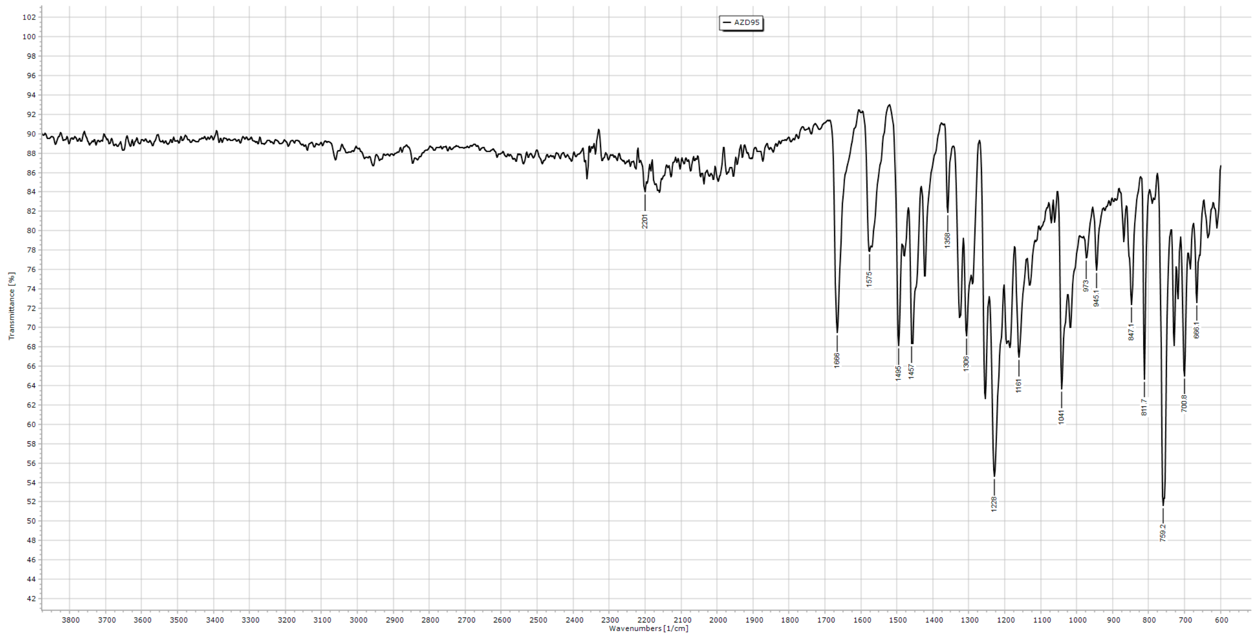

^{1}H NMR (500 MHz, $CDCl_3$)- **1h**

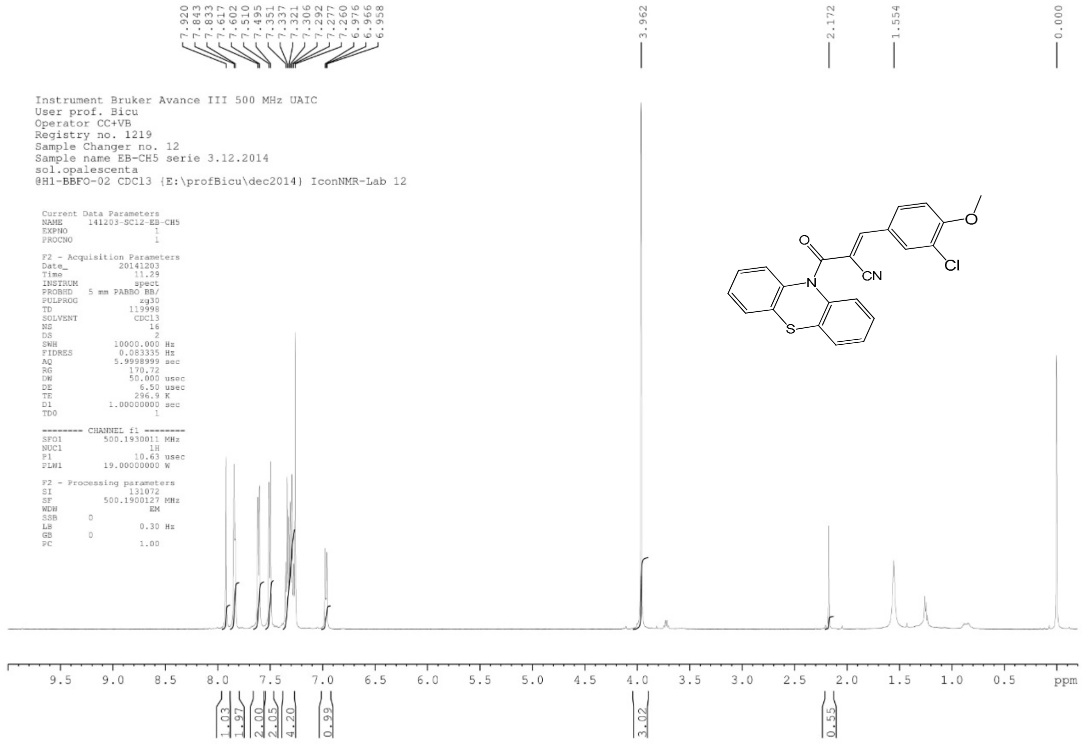

^{13}C NMR (125 MHz, $CDCl_3$)**-1h**

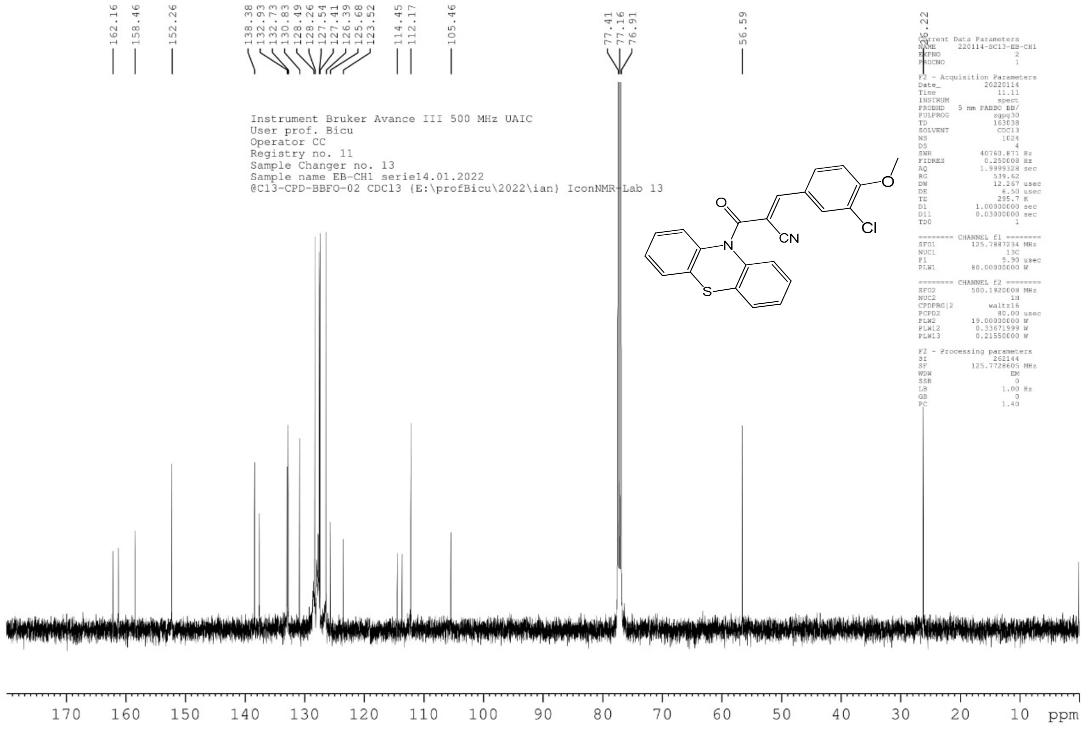

IR-1h

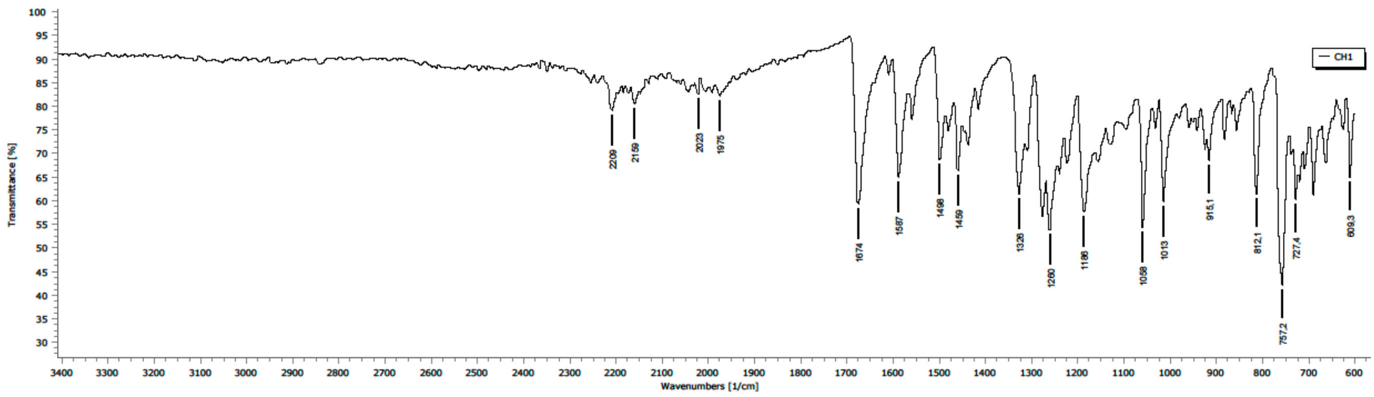

^{1}H **NMR** (500 MHz, $CDCl_3$)- **1i**

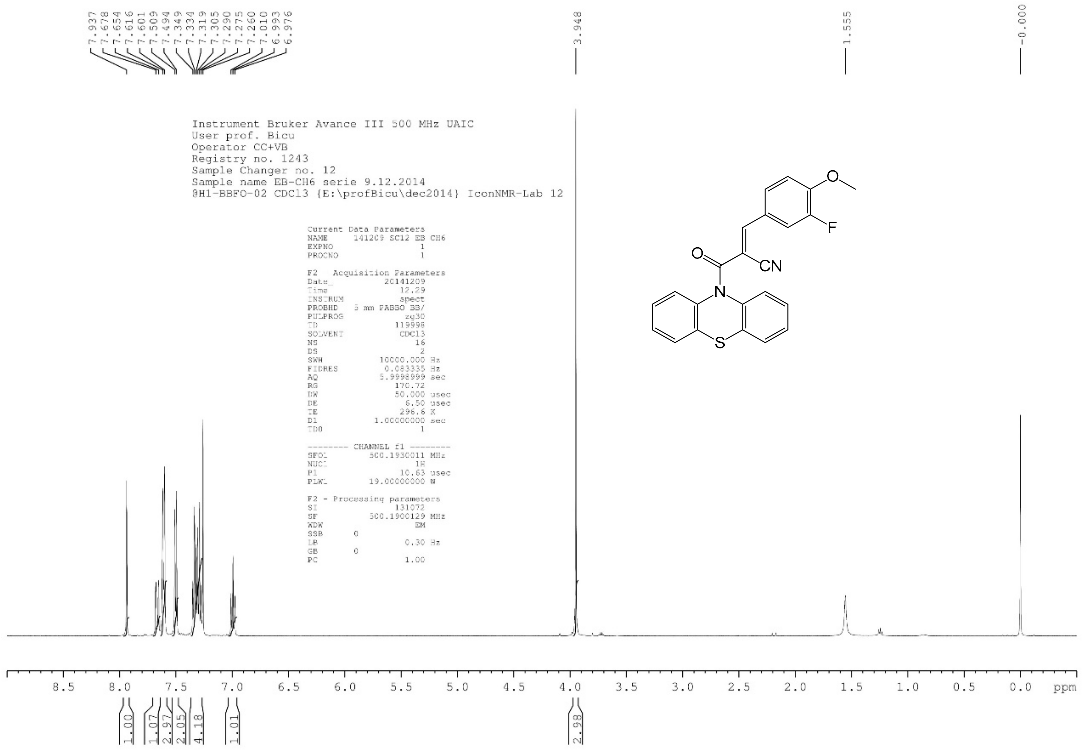

^{13}C **NMR** (125 MHz, $CDCl_3$)-**1i**

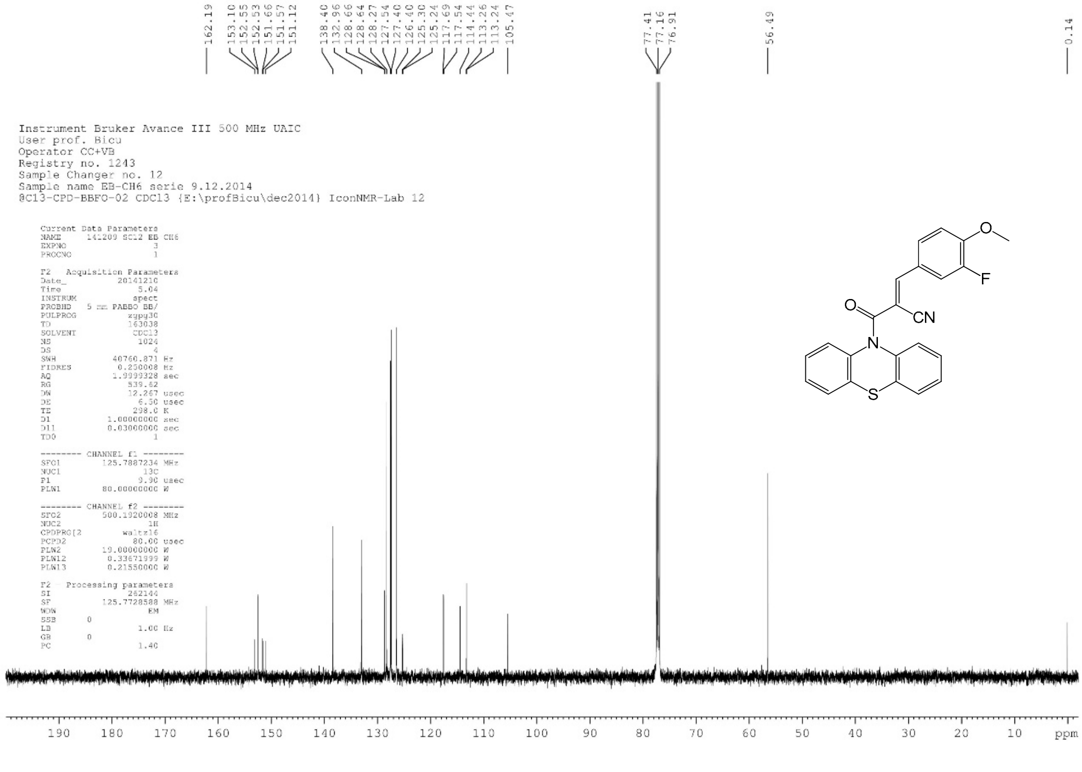

IR-1i

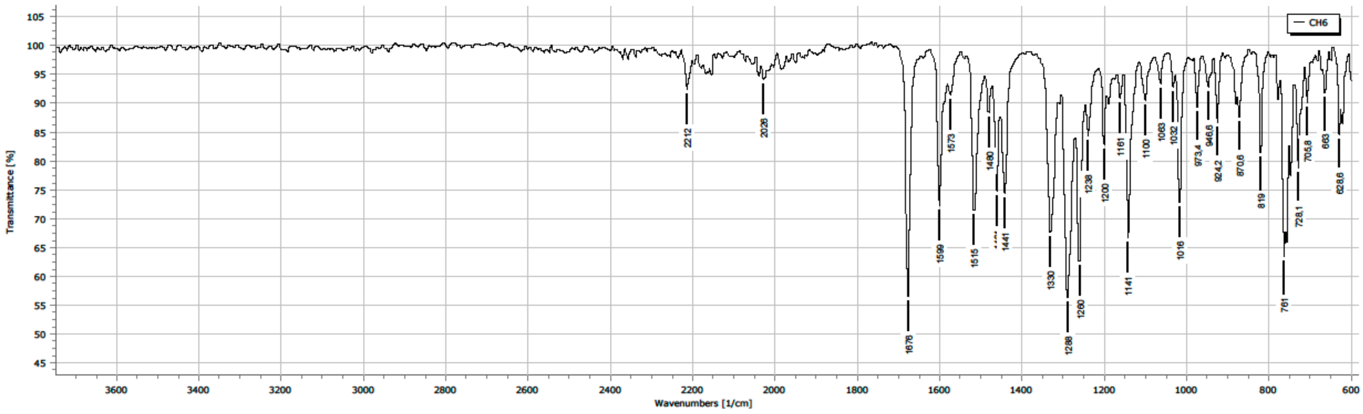

^{1}H NMR (500 MHz, $CDCl_3$)- **1j**

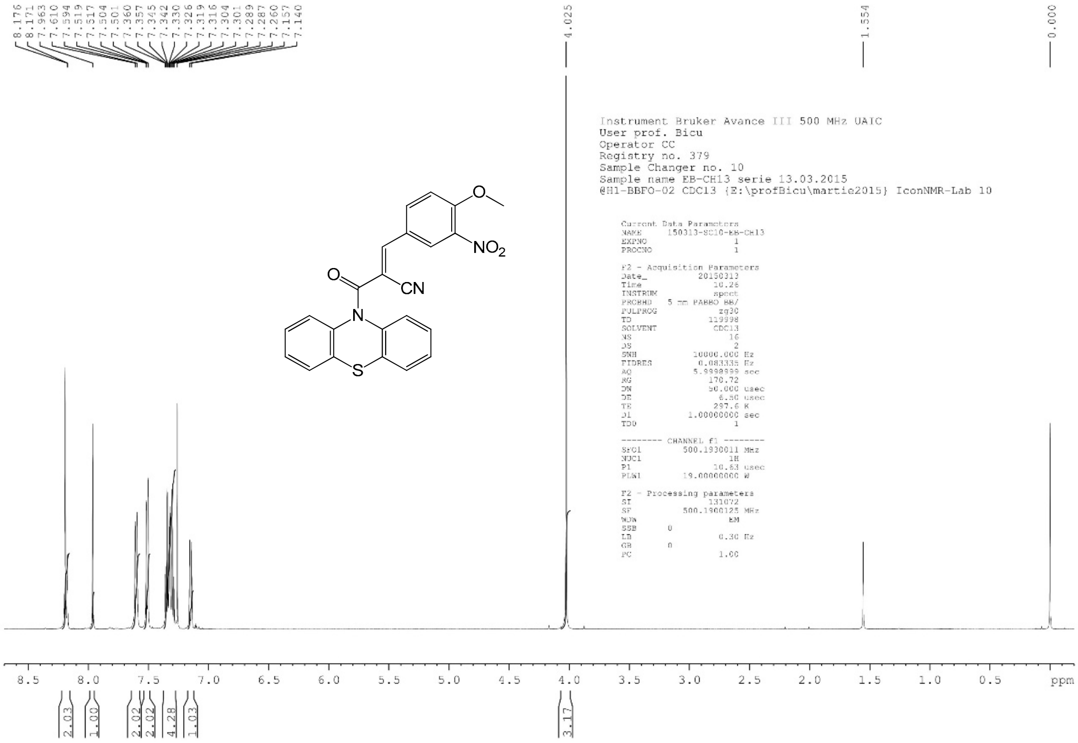

^{13}C NMR (125 MHz, CDCl$_3$)-**1j**

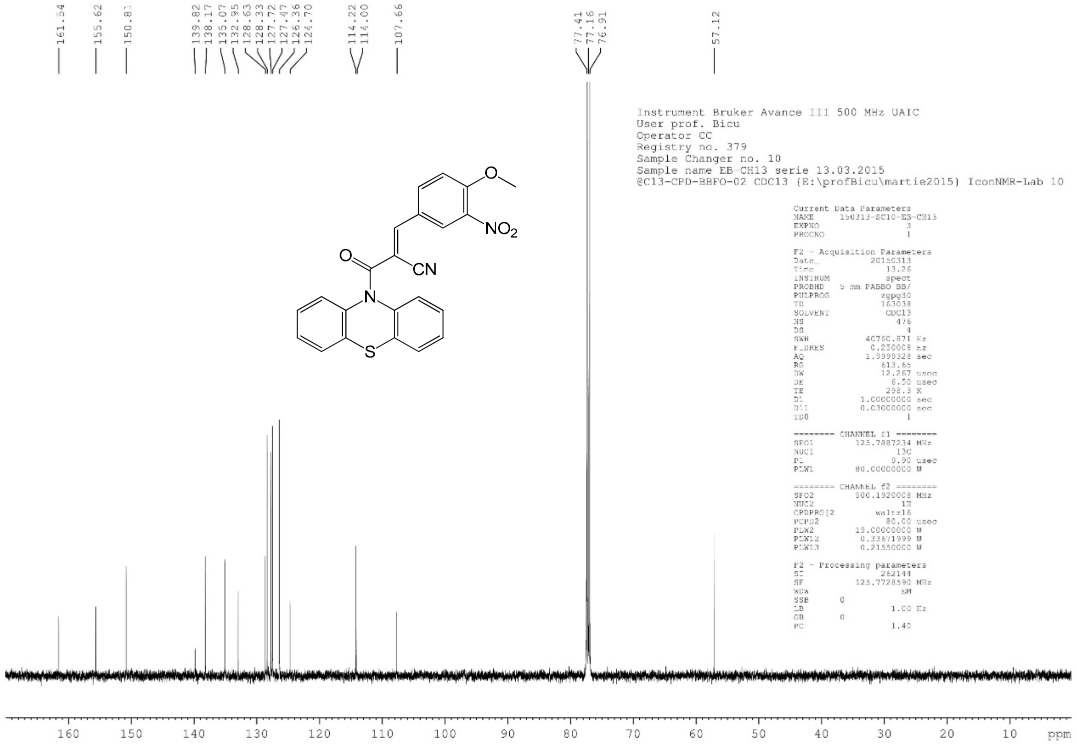

IR-1j

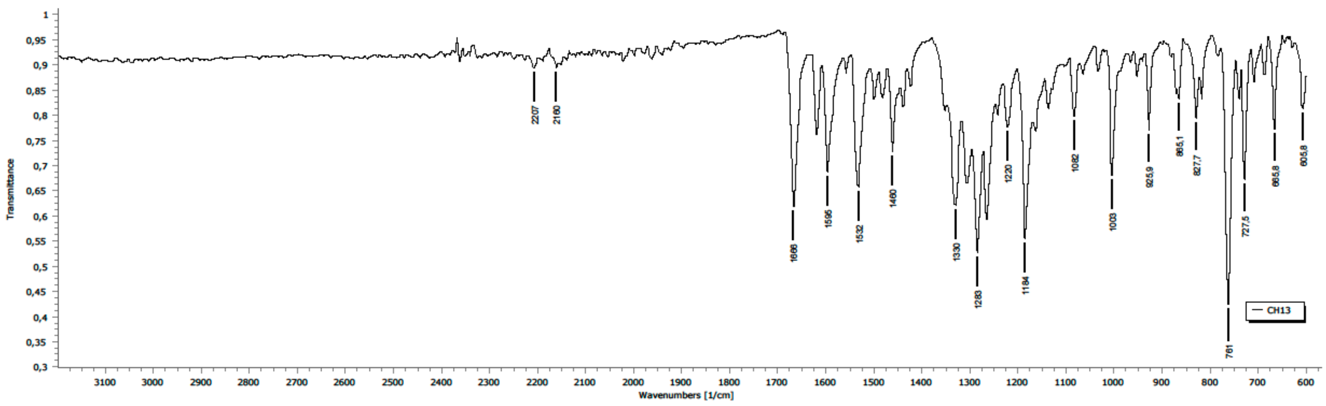

^{1}**H NMR** (500 MHz, $CDCl_3$)- **1k**

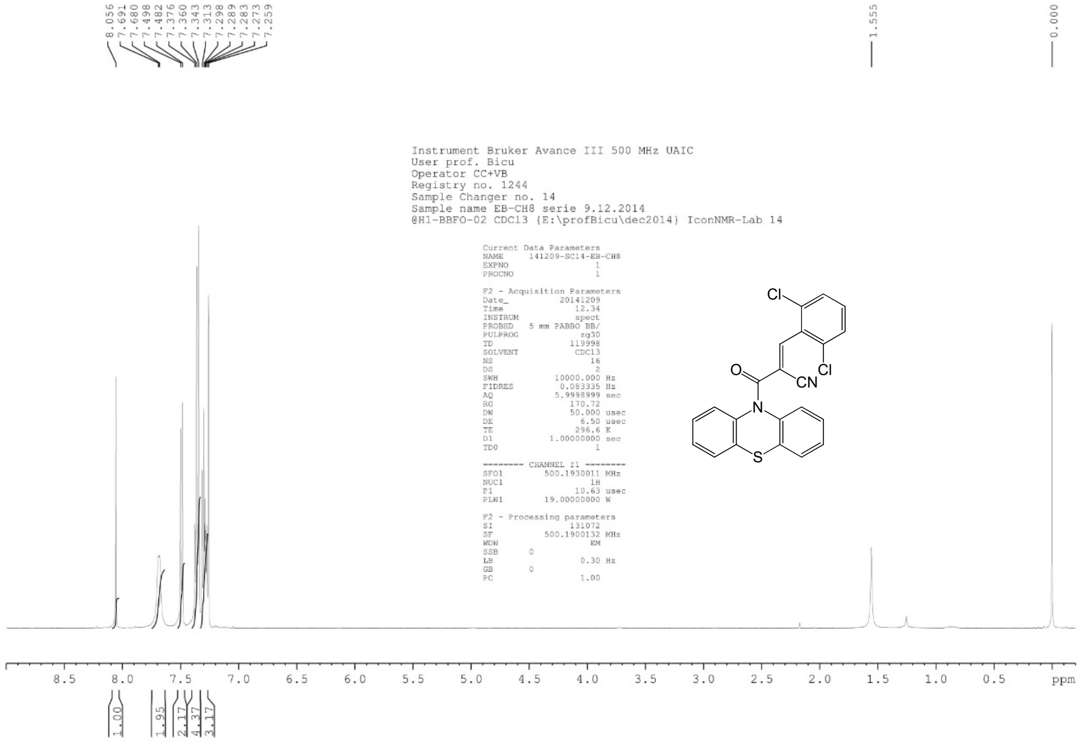

^{13}**C NMR** (125 MHz, $CDCl_3$)-**1k**

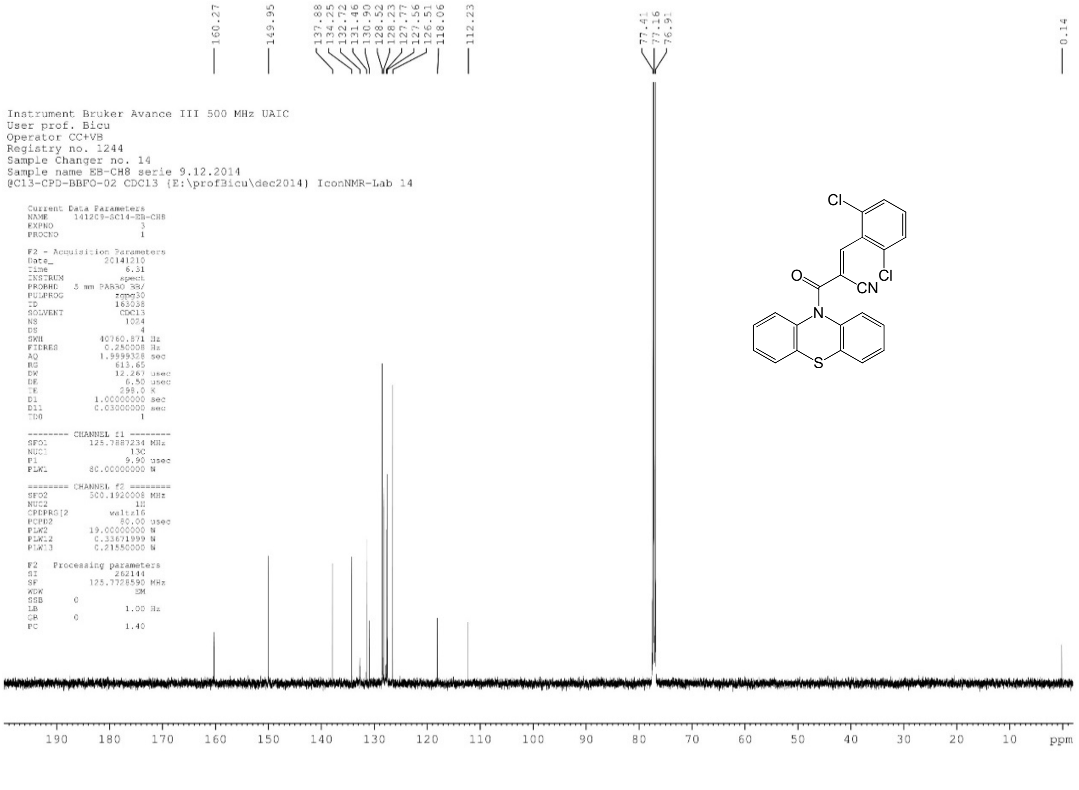

IR-1k

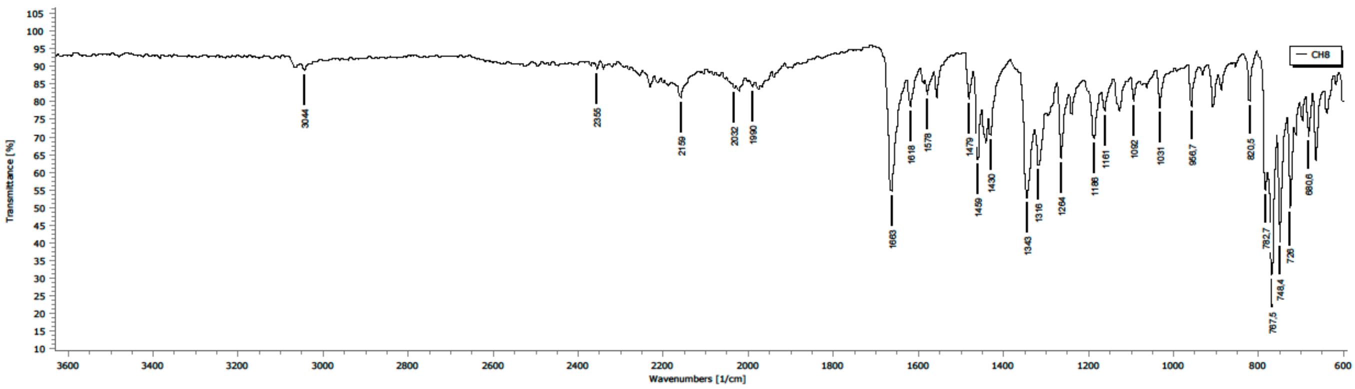

^{1}H NMR (400 MHz, $CDCl_3$)- **1l**

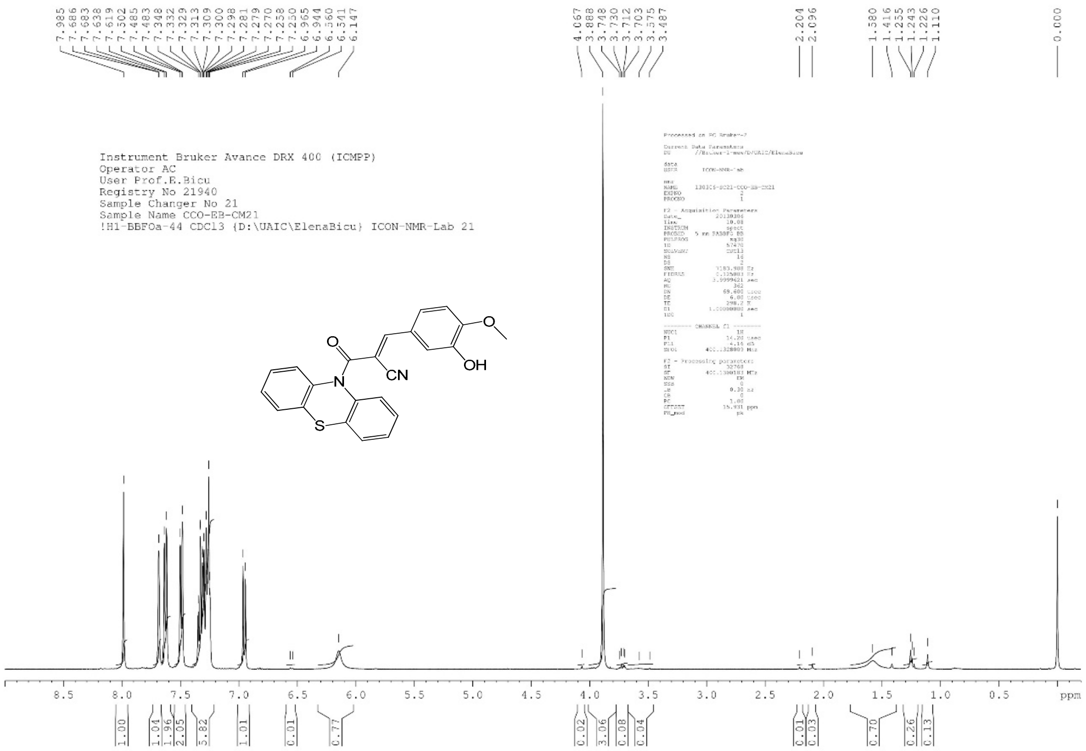

^{13}C NMR (100 MHz, $CDCl_3$)-**1l**

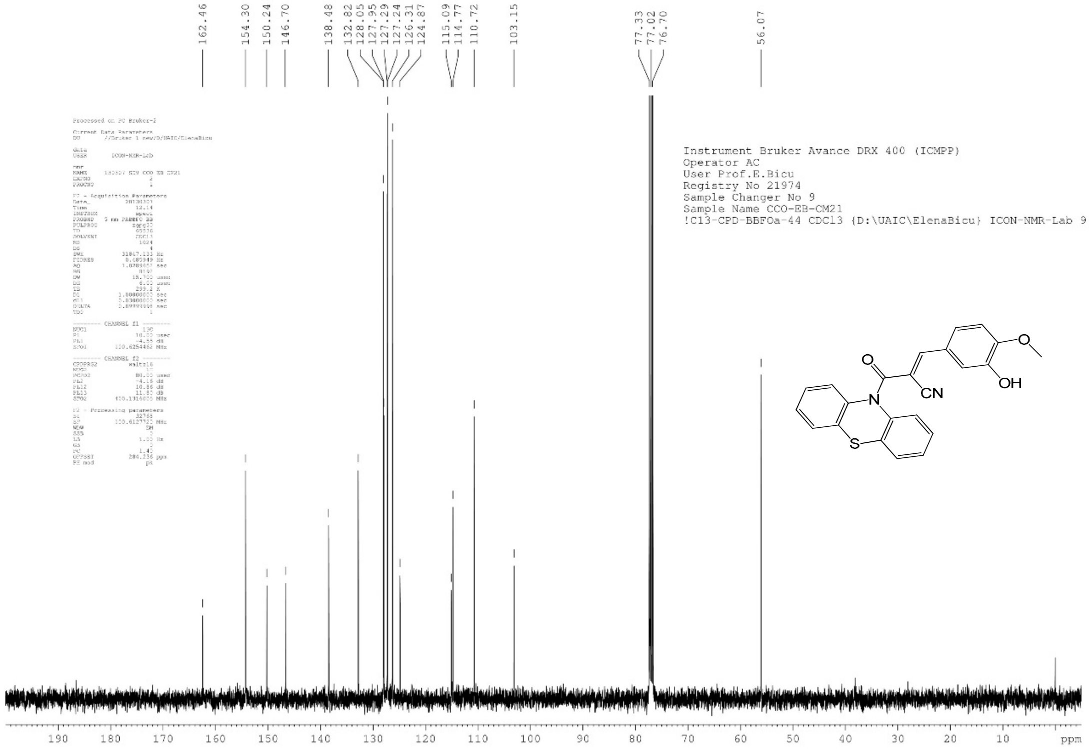

IR-1l

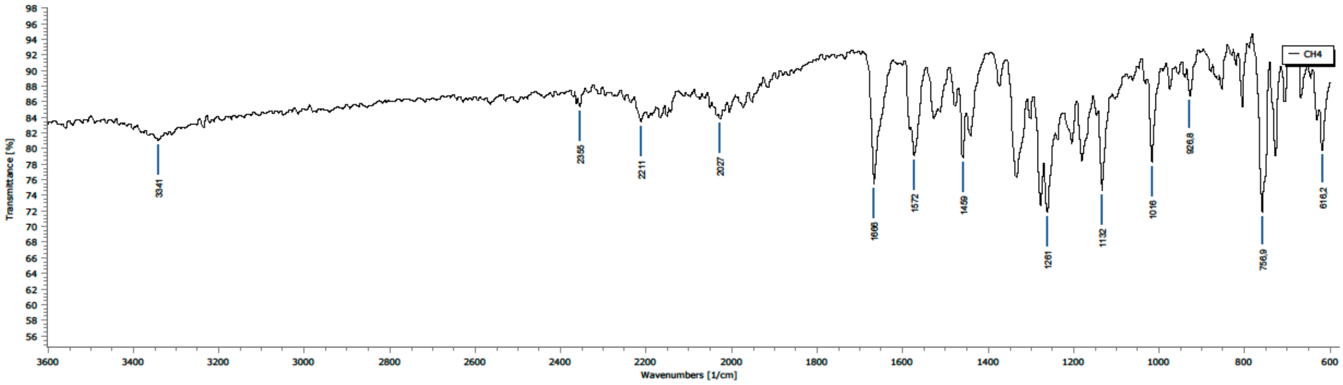

^{1}H NMR (400 MHz, $CDCl_3$)-**1m**

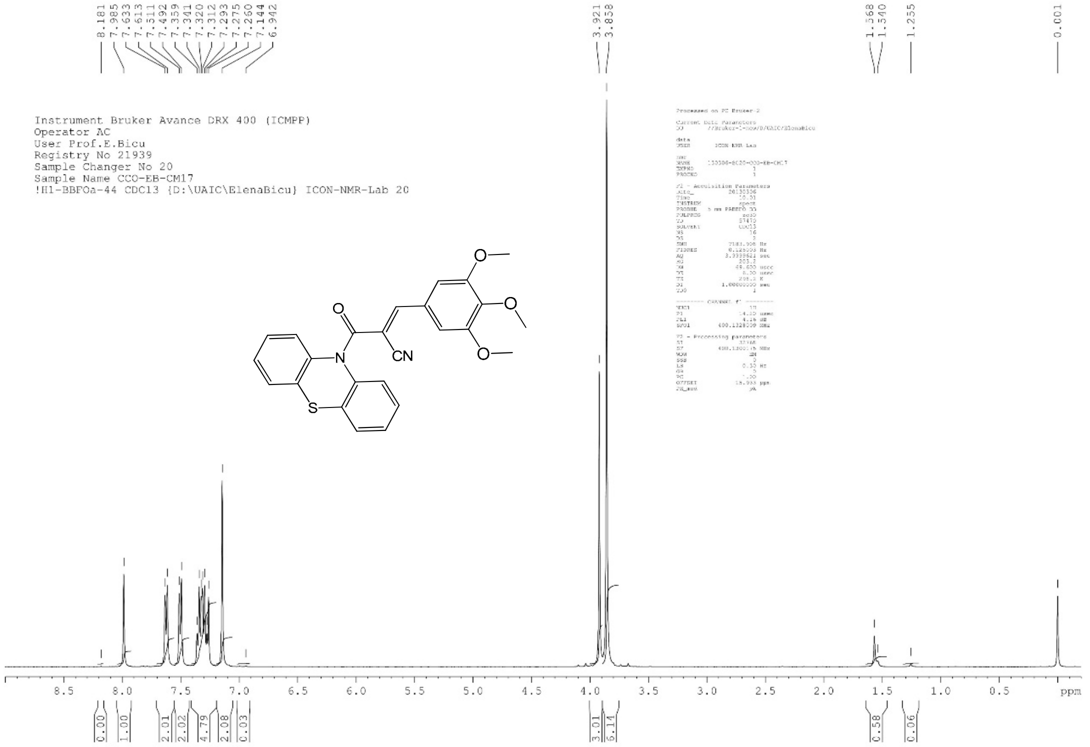

^{13}C NMR (100 MHz, $CDCl_3$)-**1m**

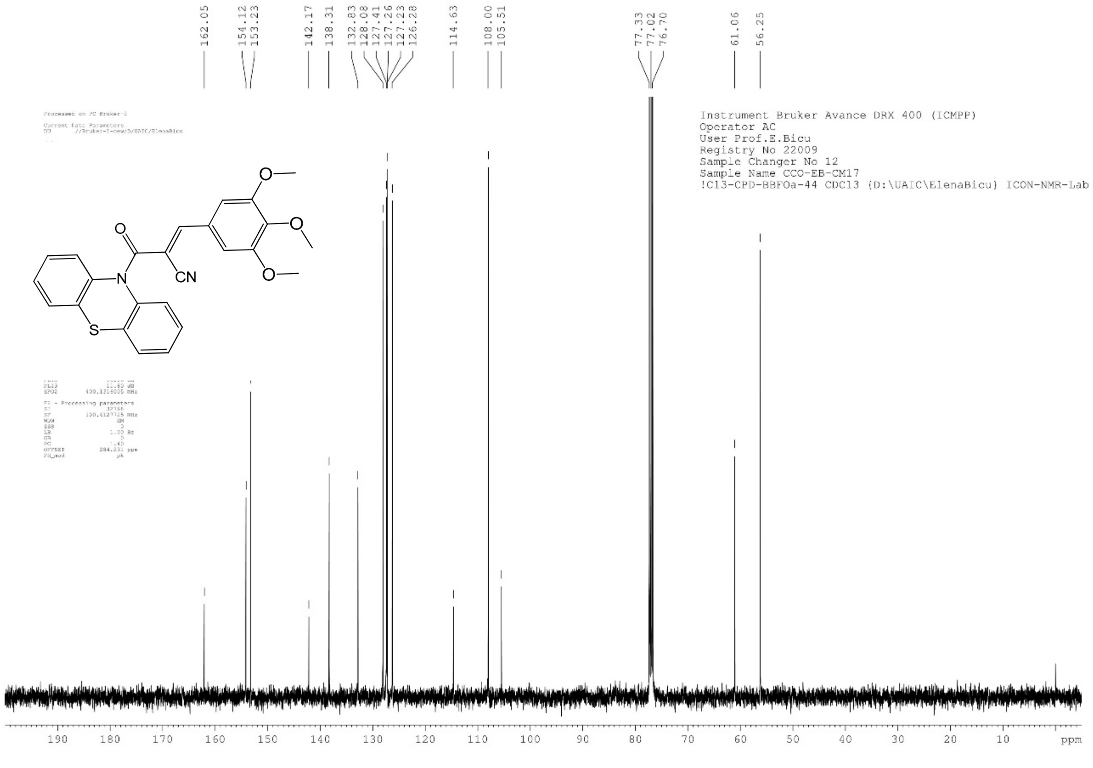

IR-1m

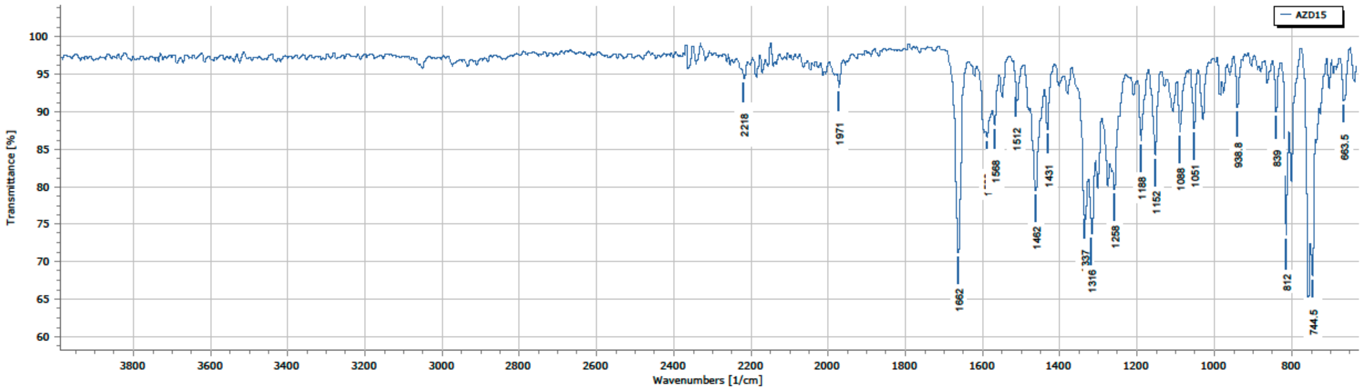

AZD15
Transmittance [%]
100
95
90
85
80
75
70
65
60
Wavenumbers [1/cm]
3800
3600
3400
3200
3000
2800
2600
2400
2200
2000
1800
1600
1400
1200
1000
800
2218
1971
1662
1568
1512
1462
1431
1316
1258
1188
1152
1088
1051
938.8
839
812
744.5
663.5

^{1}H NMR (400 MHz, $CDCl_3$)- **1n**

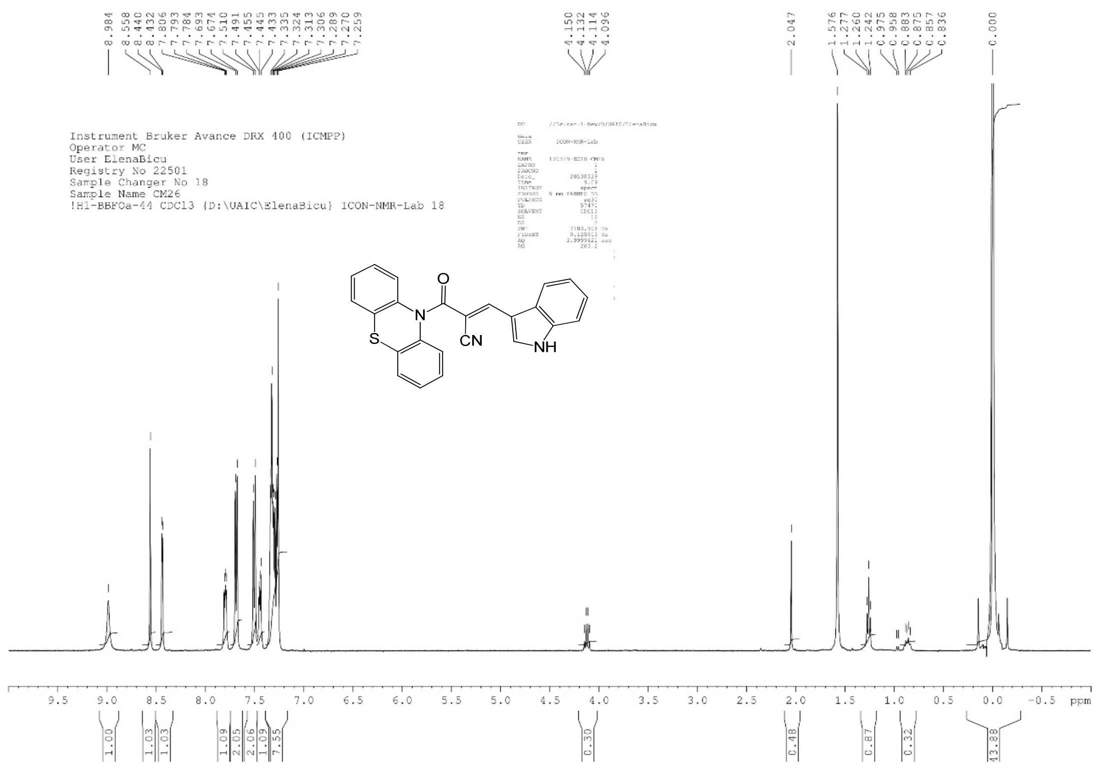

^{13}C NMR (100 MHz, $CDCl_3$)**-1n**

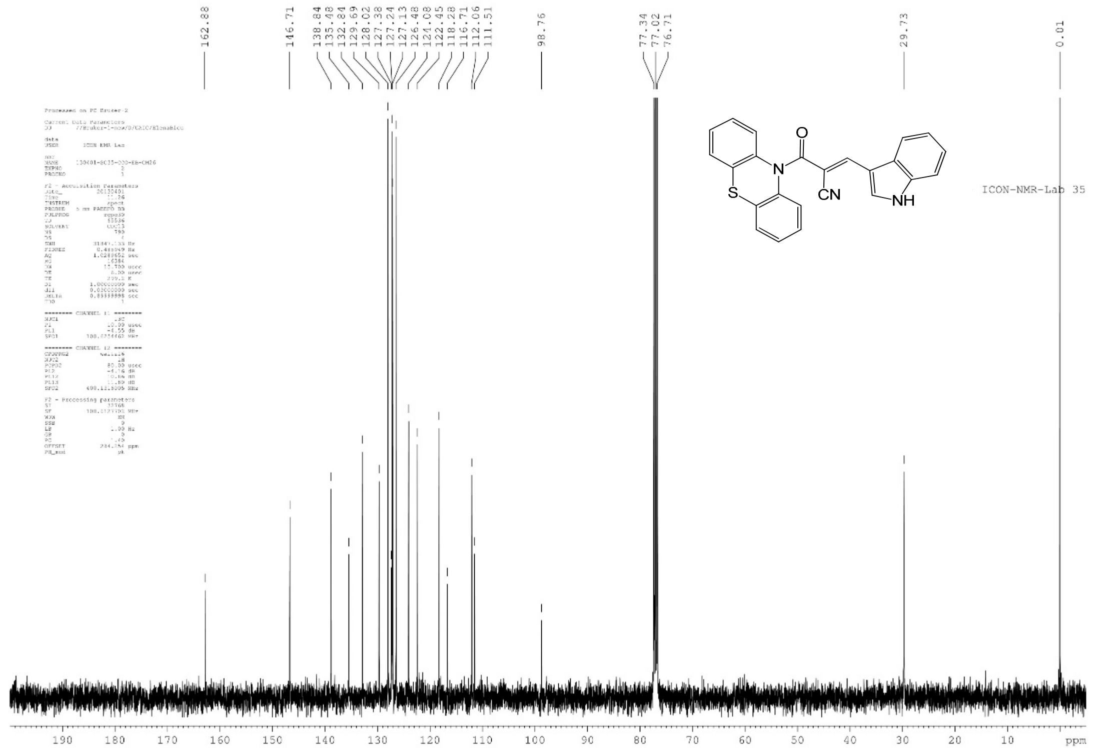

IR-1n

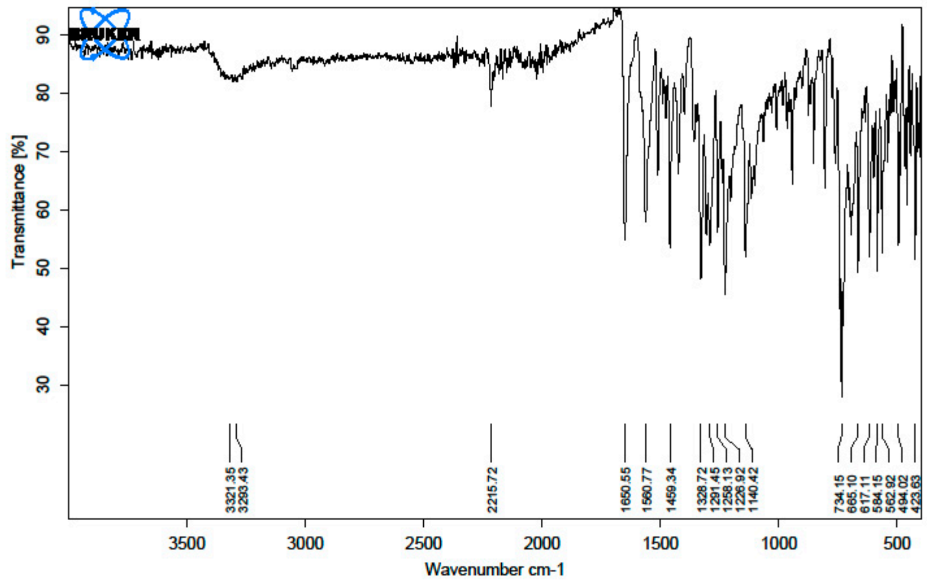

^{1}H NMR (500 MHz, CDCl3)- **1o**

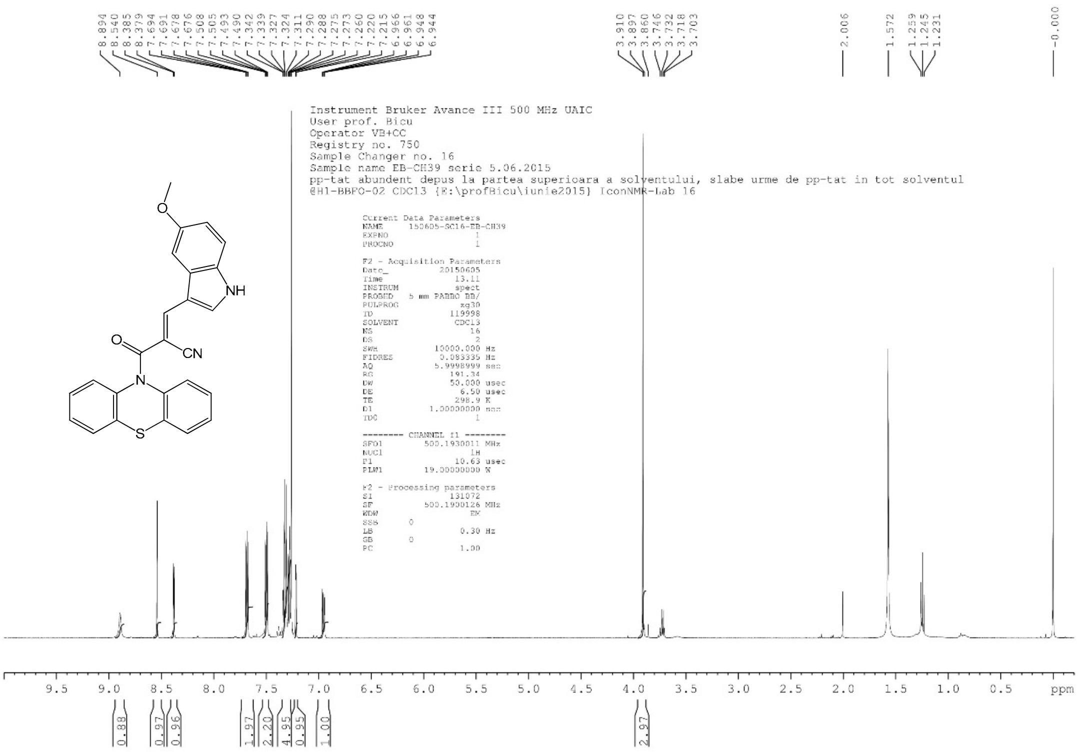

^{13}C NMR (125 MHz, $CDCl_3$)**-1o**

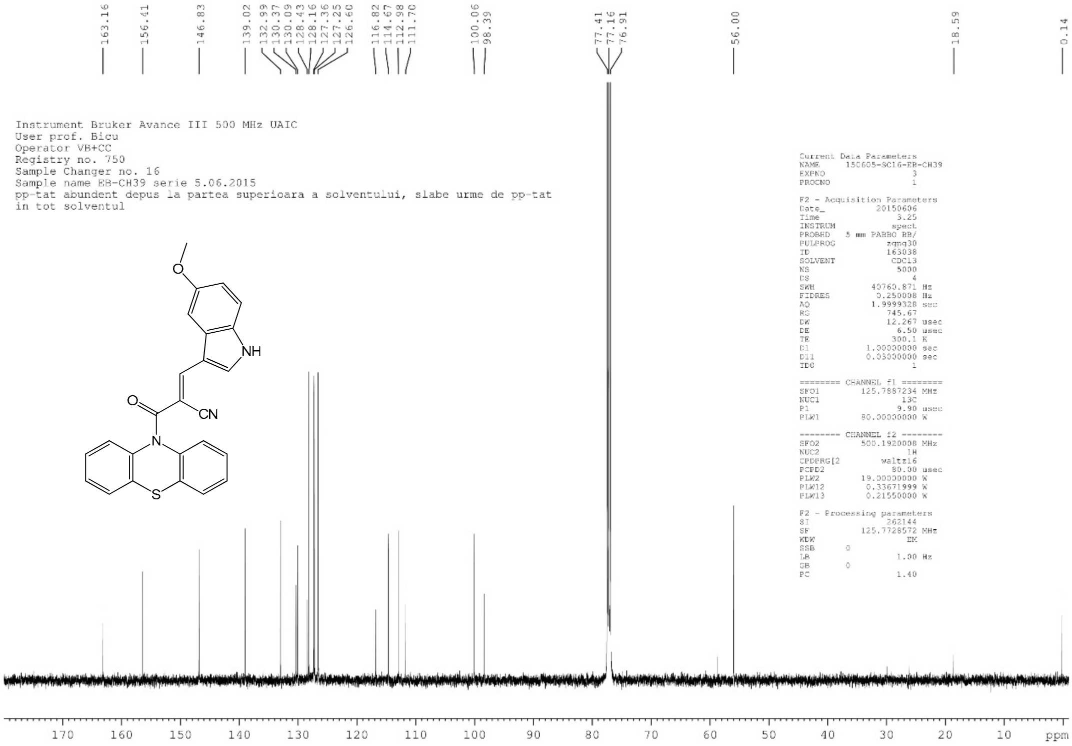

IR-1o

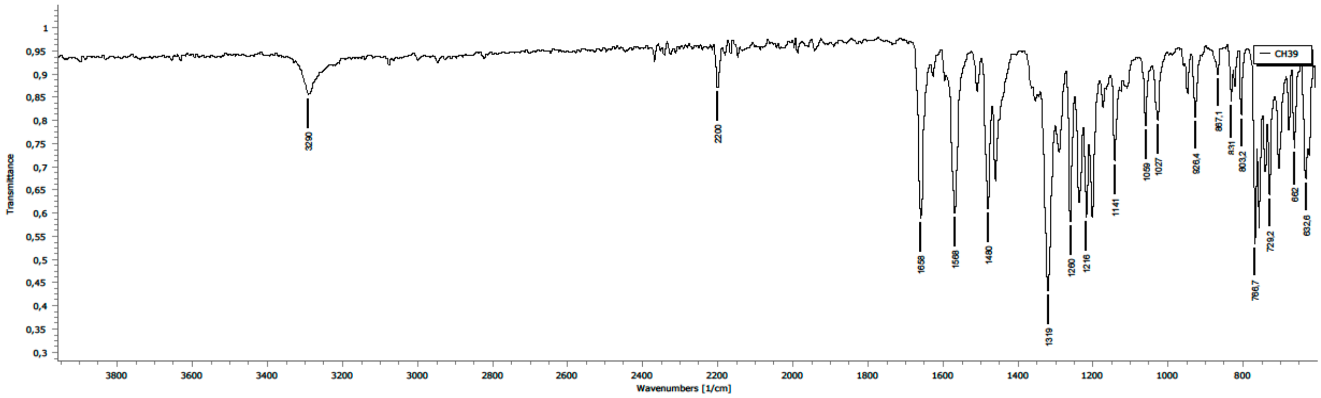

^{1}H NMR (500 MHz, $CDCl_3$)- **1p**

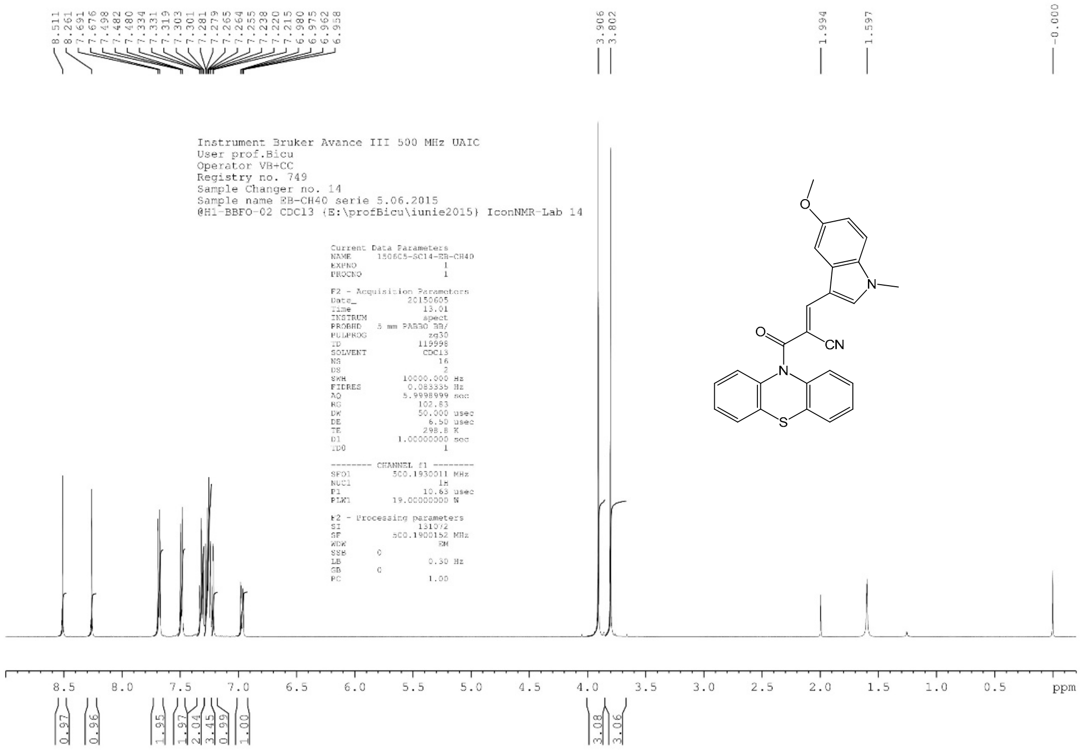

^{13}C NMR (125 MHz, $CDCl_3$)-**1p**

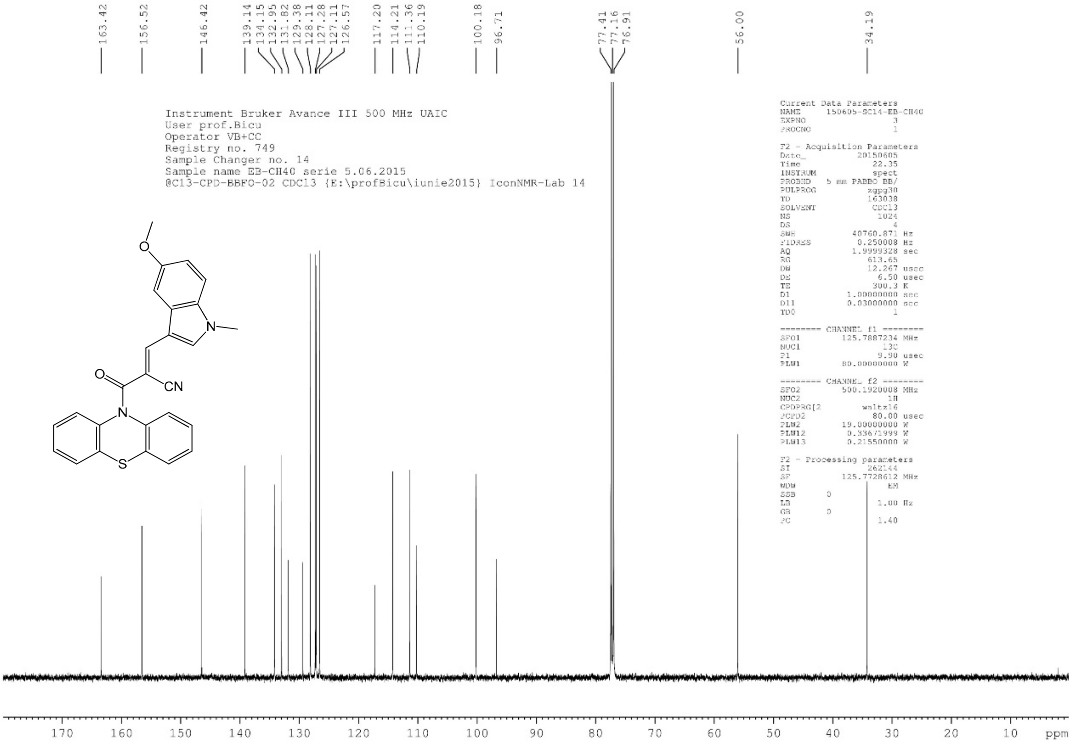

IR-1p

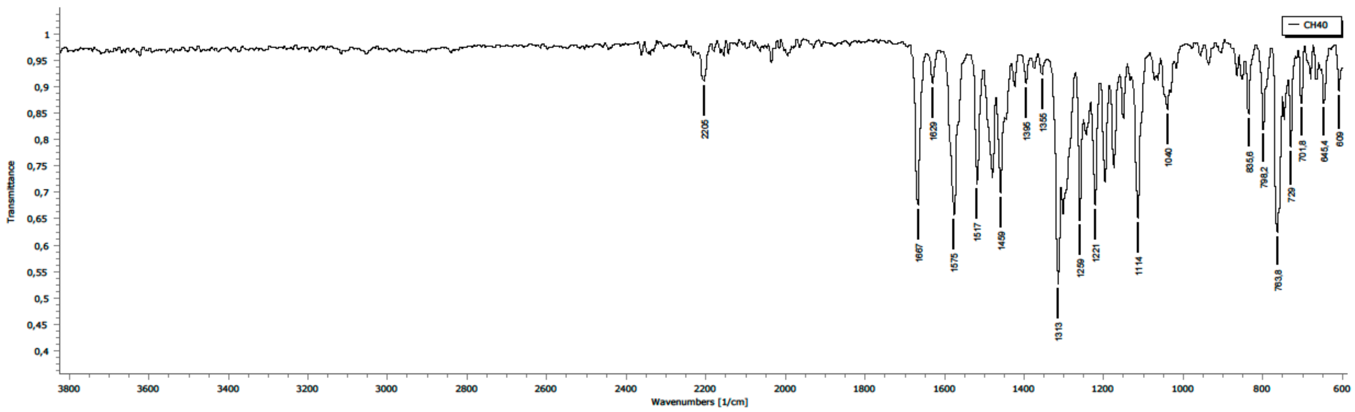

^{1}H NMR (400 MHz, $CDCl_3$)- **1q**

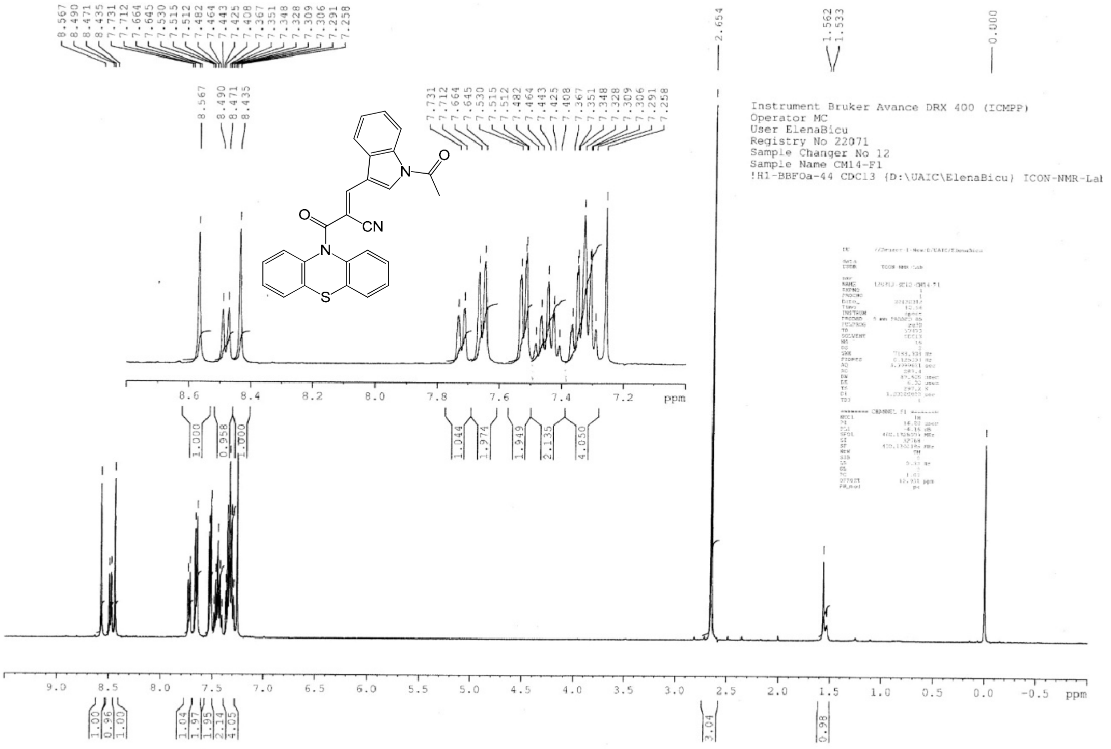

^{13}C NMR (100 MHz, CDCl$_3$)-**1q**

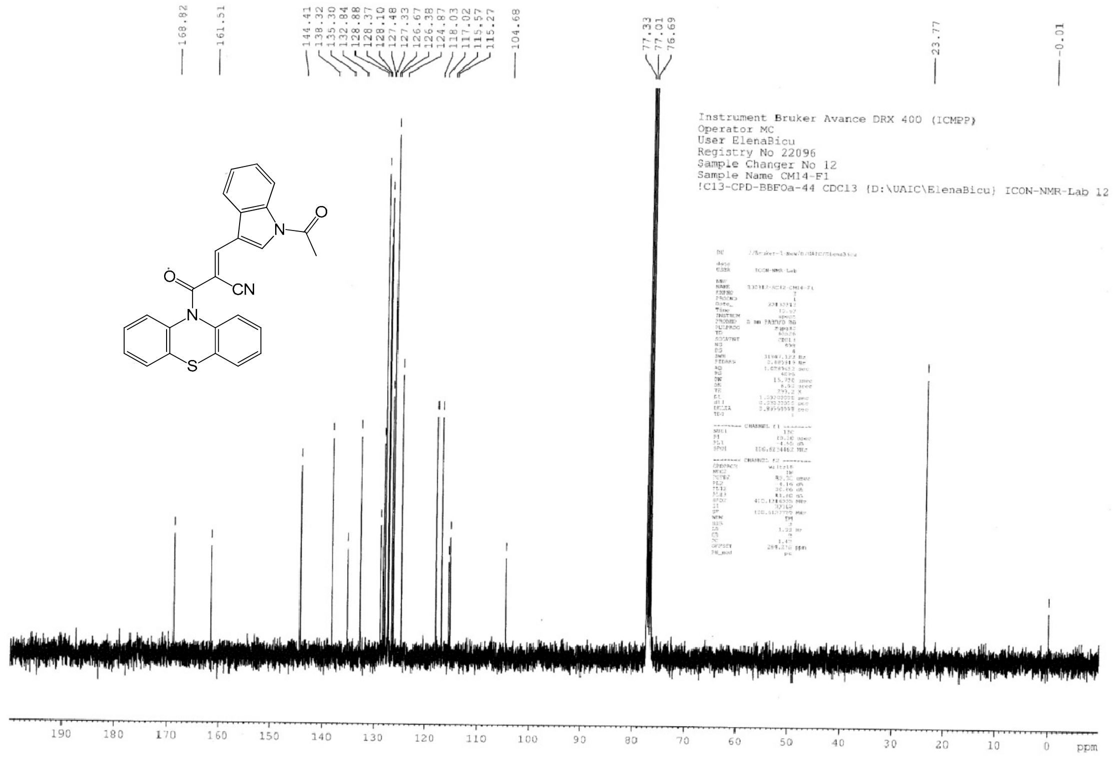

^{1}H NMR (500 MHz, CDCl$_3$)- **1r**

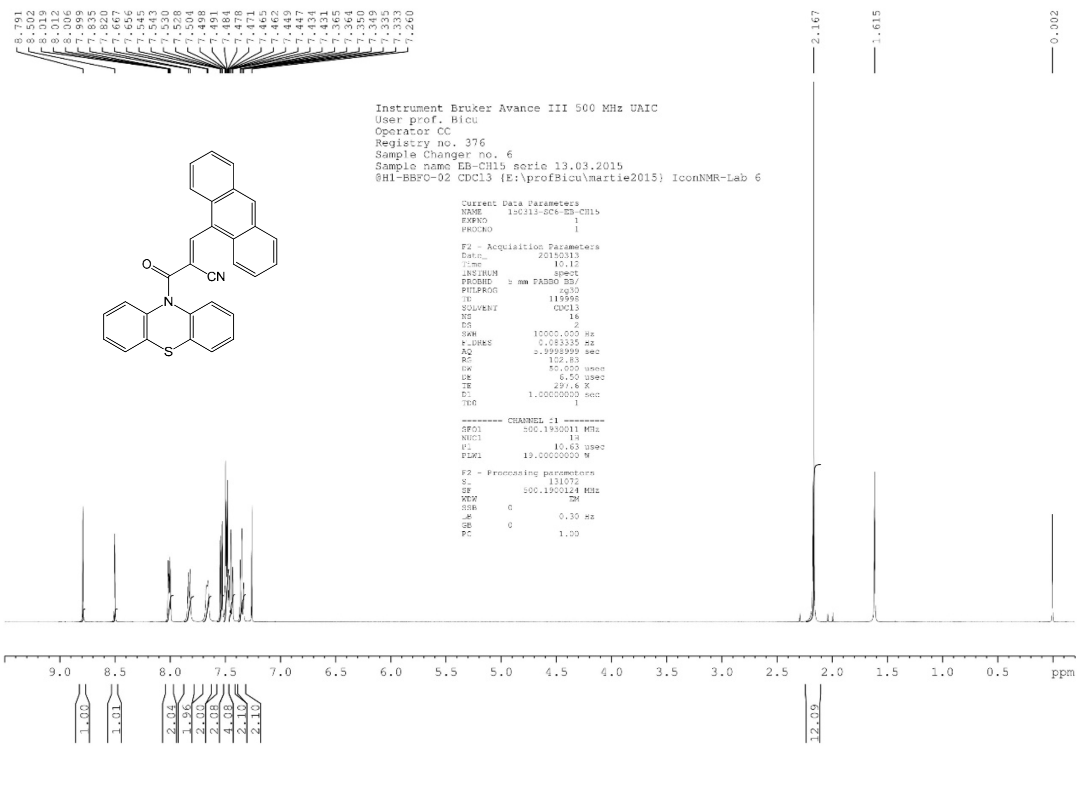

^{13}C NMR (125 MHz, $CDCl_3$)-**1r**

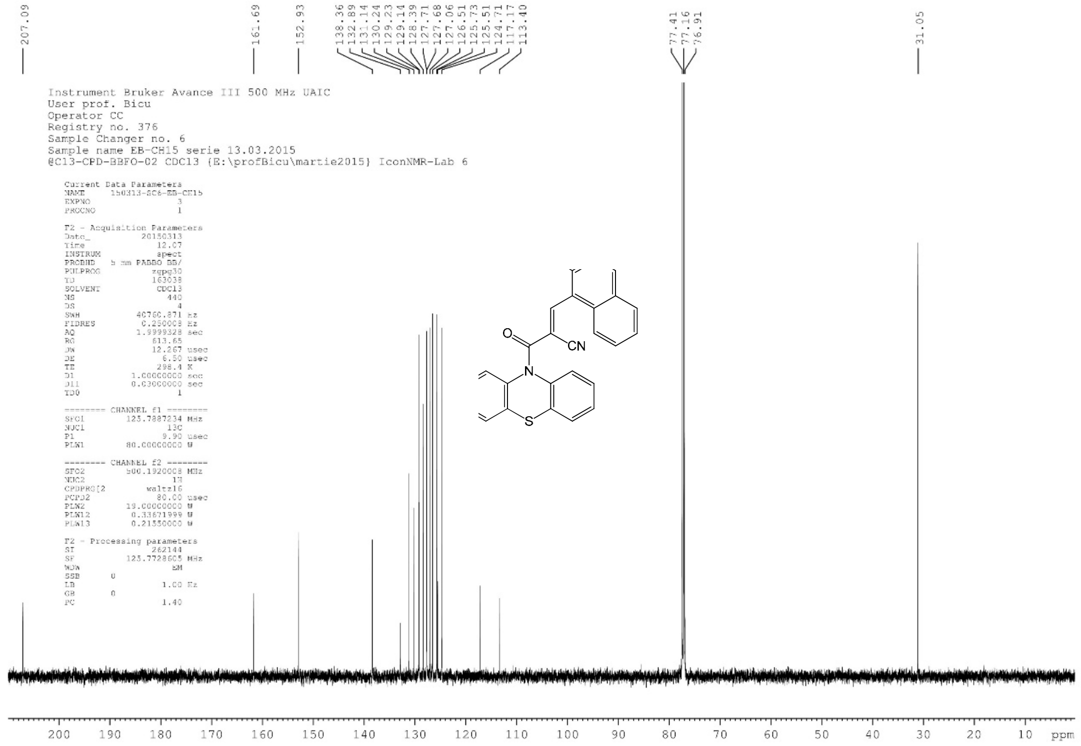

IR-1r

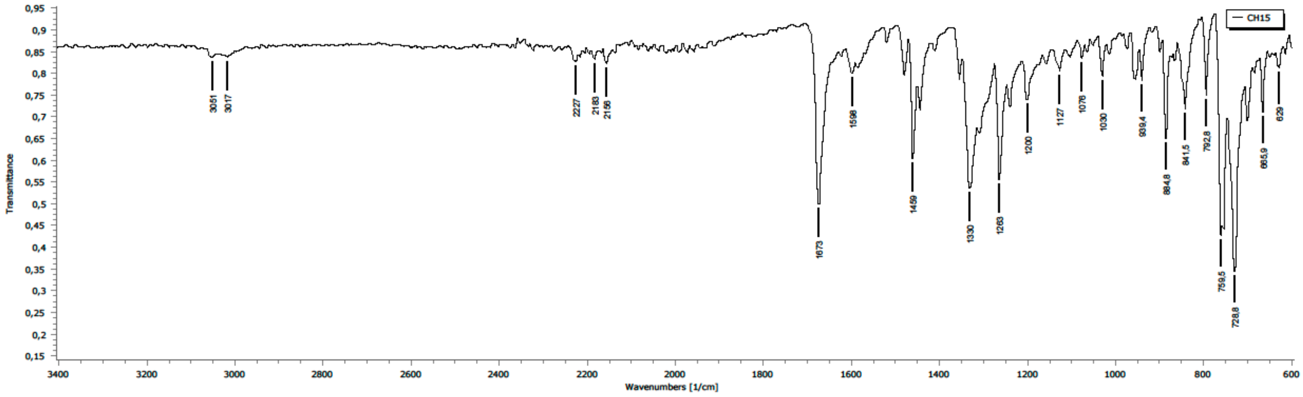

^{1}H NMR (400 MHz, $CDCl_3$)-**2a**

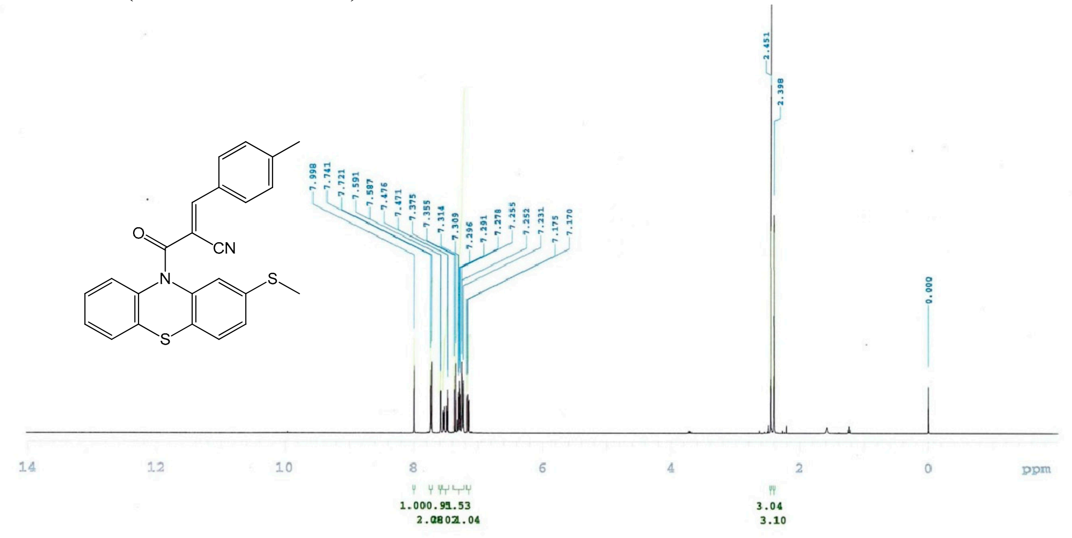

^{13}C NMR (100 MHz, $CDCl_3$)-**2a**

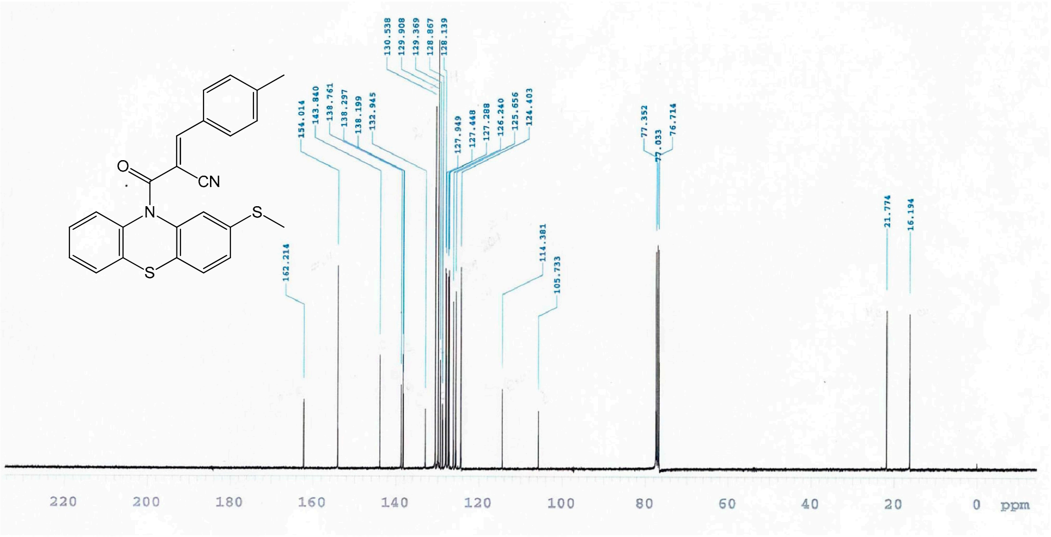

IR-2a

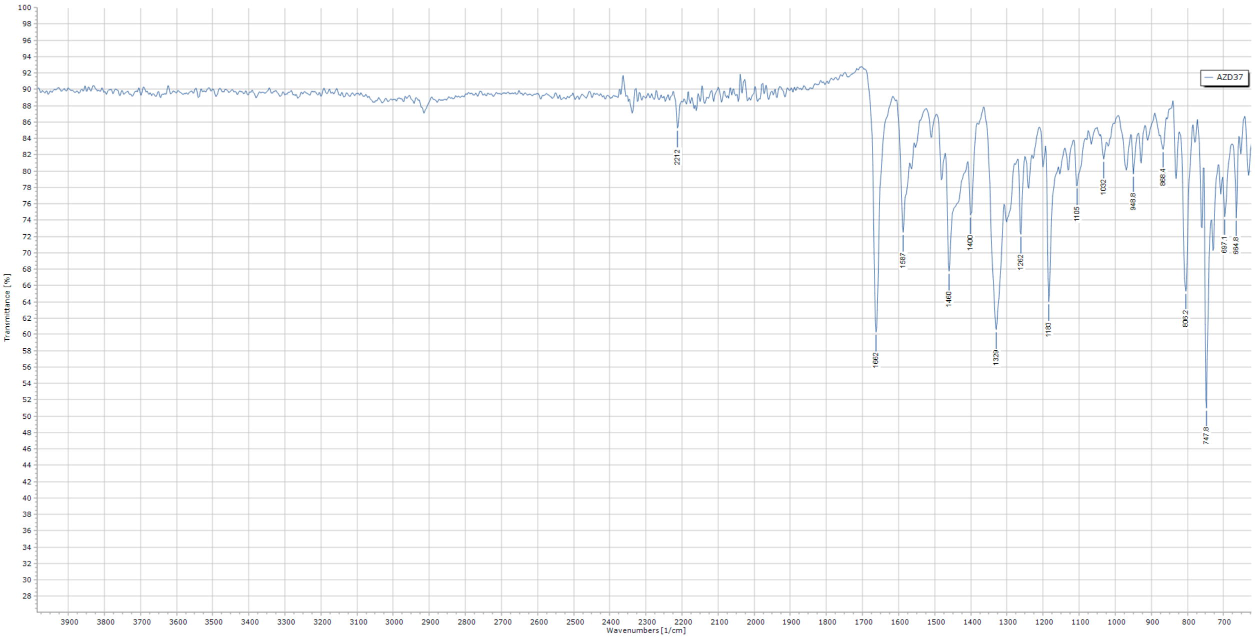
AZD37
Transmittance [%]
Wavenumbers [1/cm]
2212
1662
1587
1460
1400
1329
1262
1183
1105
1032
948.8
868.4
806.2
747.8
697.1
664.8

^{1}H NMR (500 MHz, $CDCl_3$)- **2b**

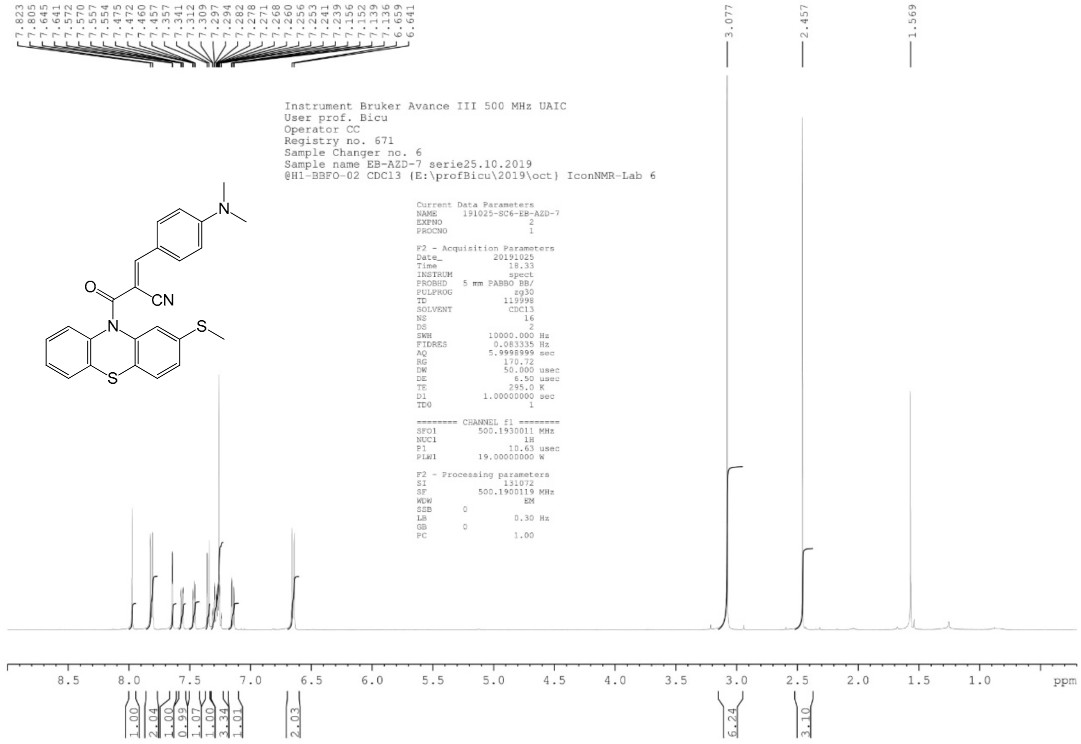

^{13}C NMR (125 MHz, $CDCl_3$)-**2b**

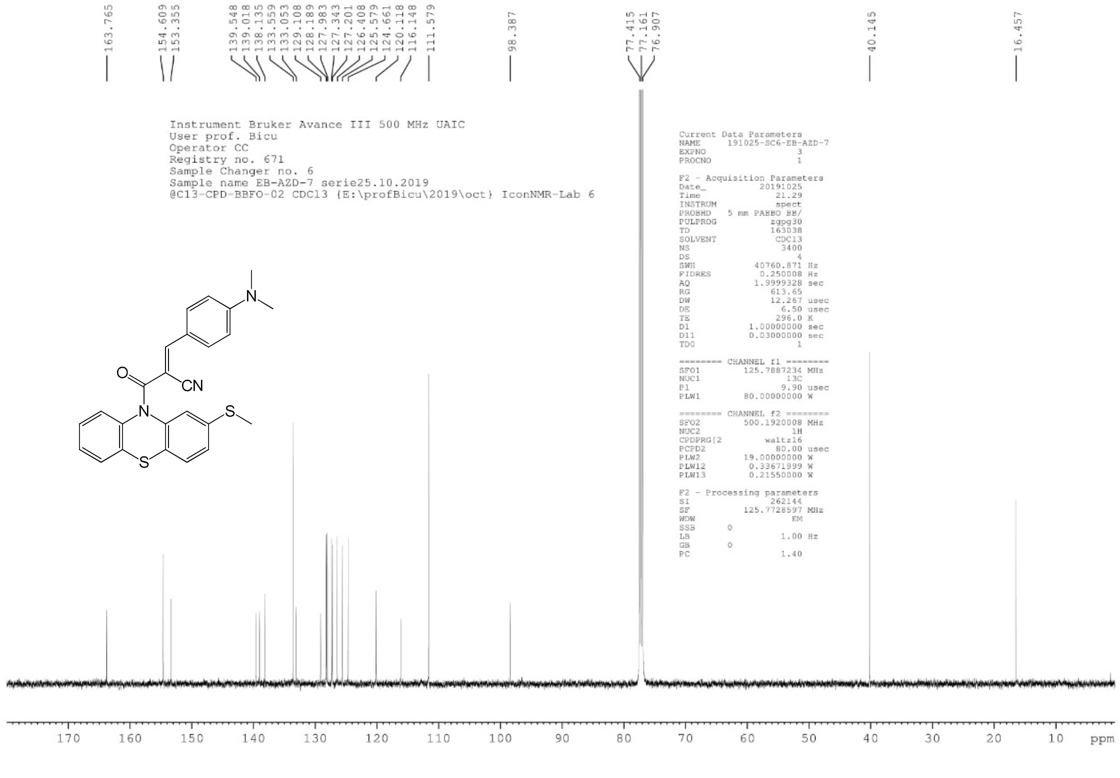

IR-2b

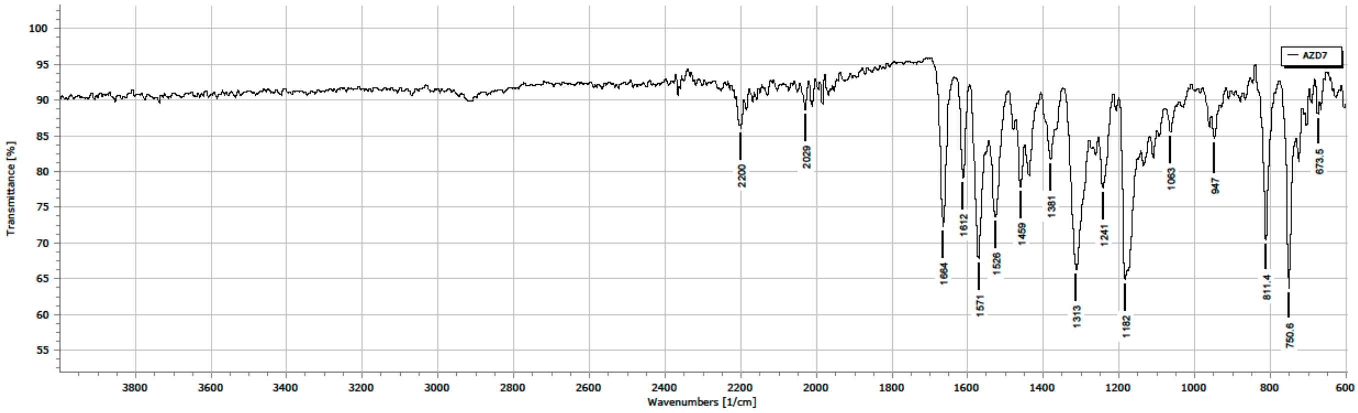

^{1}H NMR (400 MHz, $CDCl_3$)-**2c**

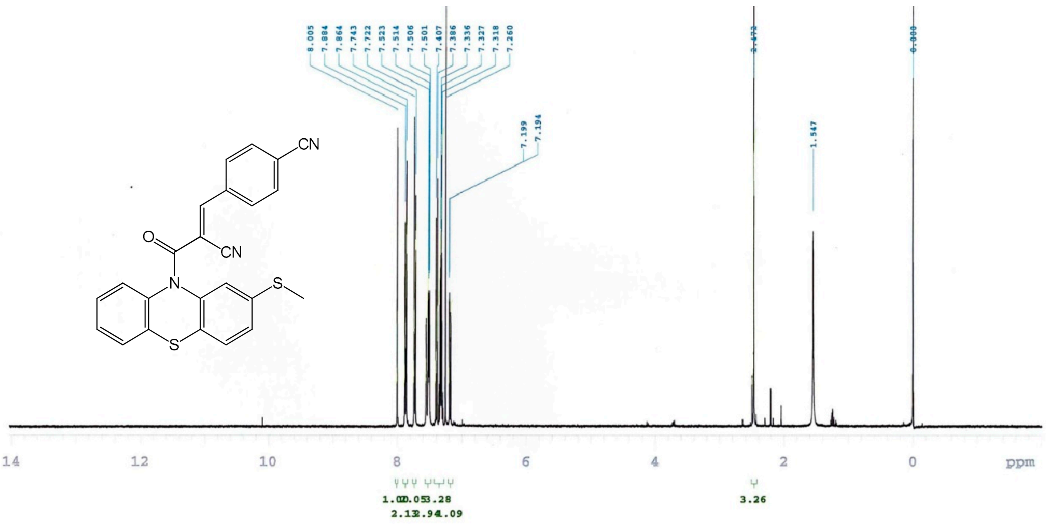

^{13}**C NMR** (100 MHz, $CDCl_3$)**-2c**

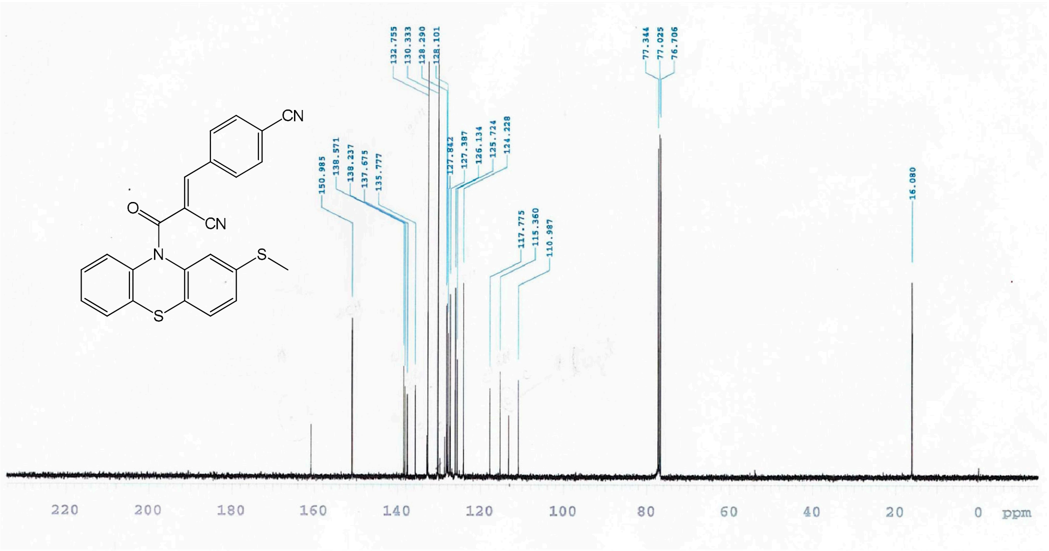

IR-2c

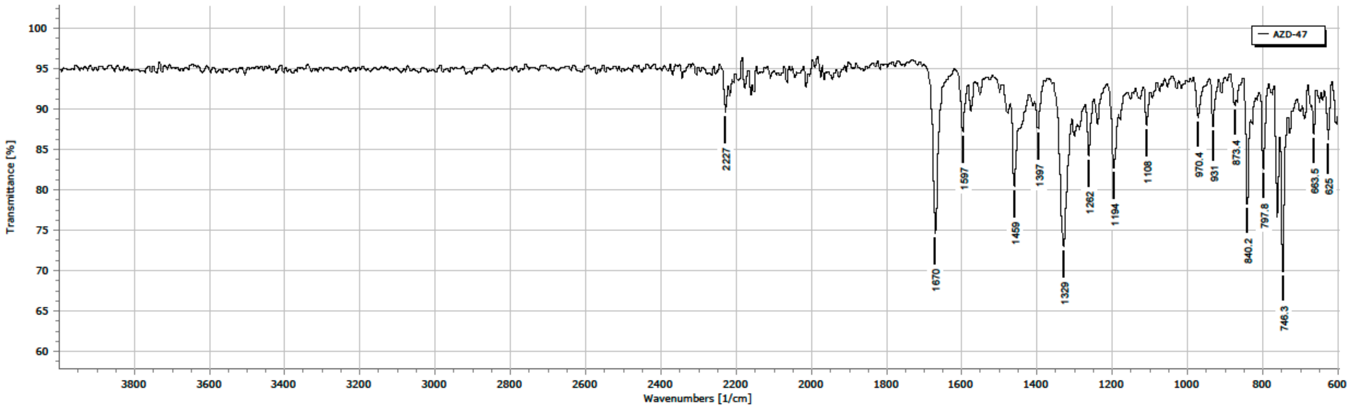

^{1}H NMR (400 MHz, $CDCl_3$)-**2d**

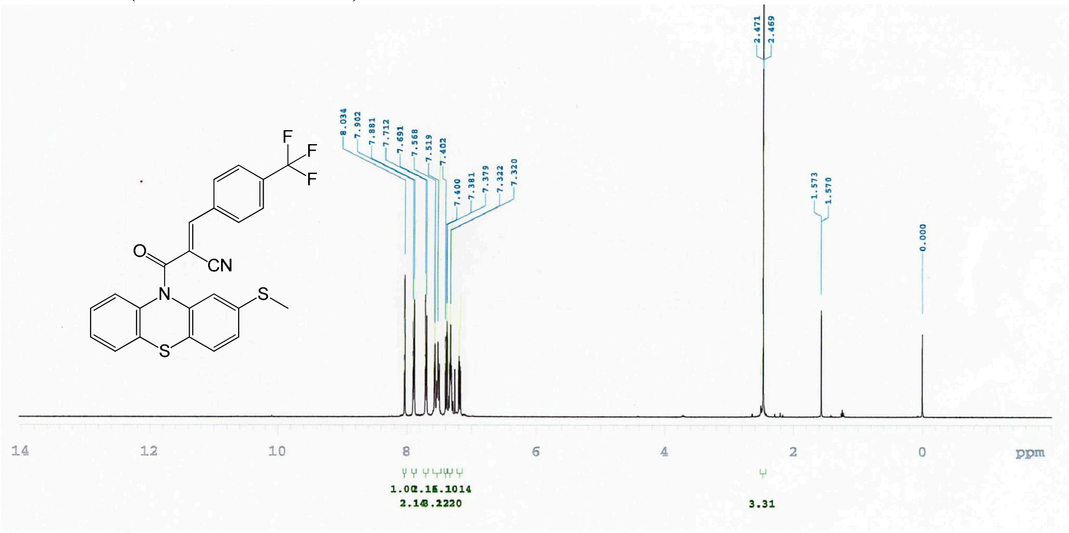

^{13}C NMR (100 MHz, $CDCl_3$)-**2d**

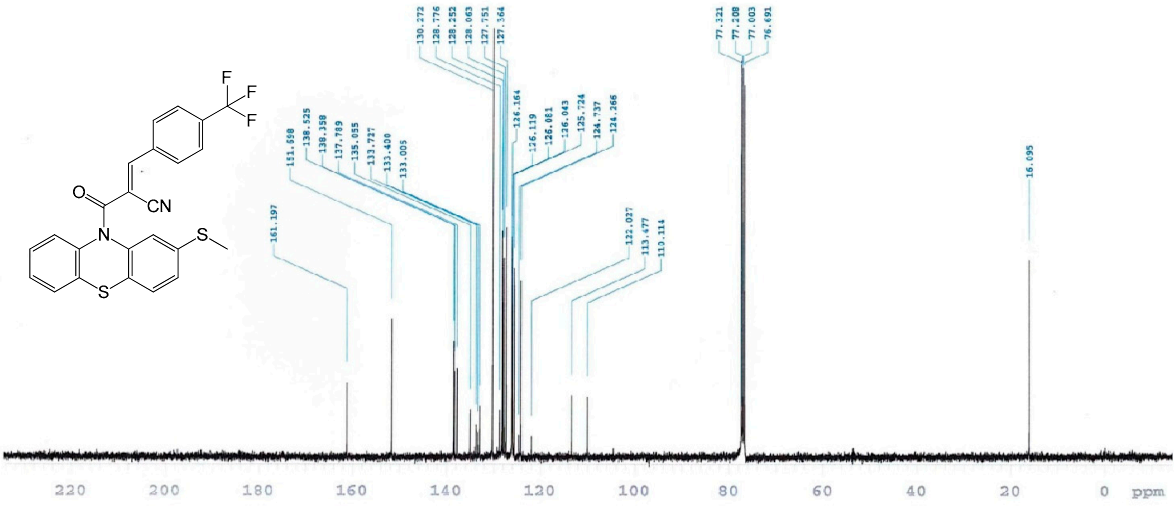

IR-2d

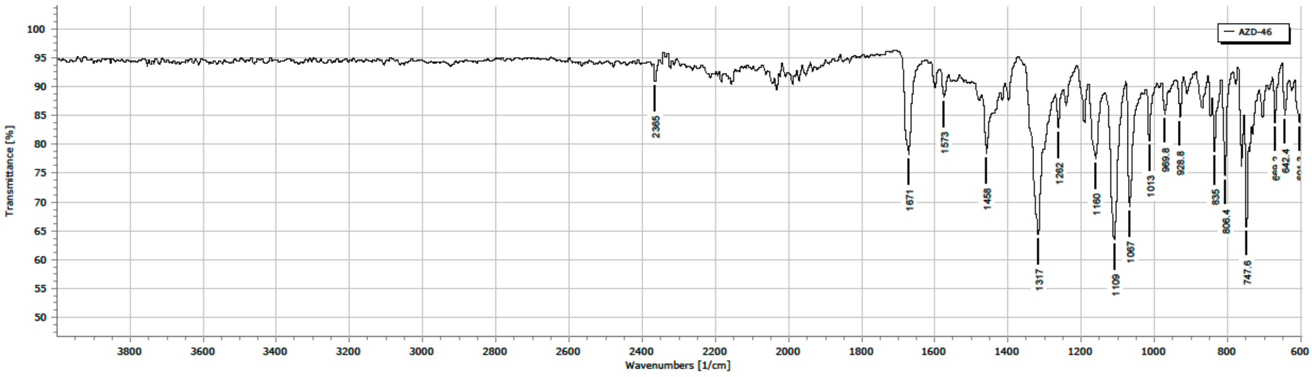

^{1}H NMR (400 MHz, $CDCl_3$)-**2e**

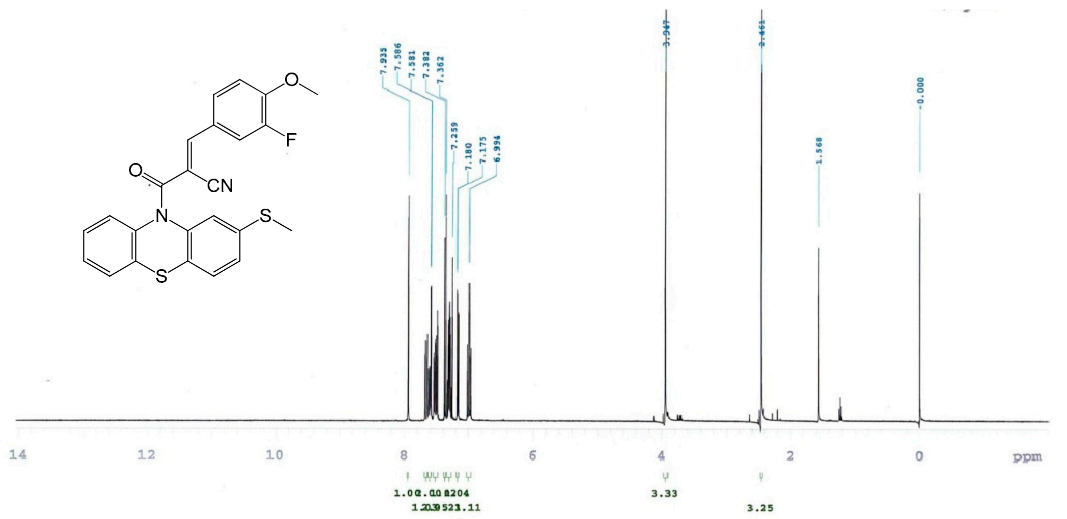

^{13}C NMR (100 MHz, $CDCl_3$)-**2e**

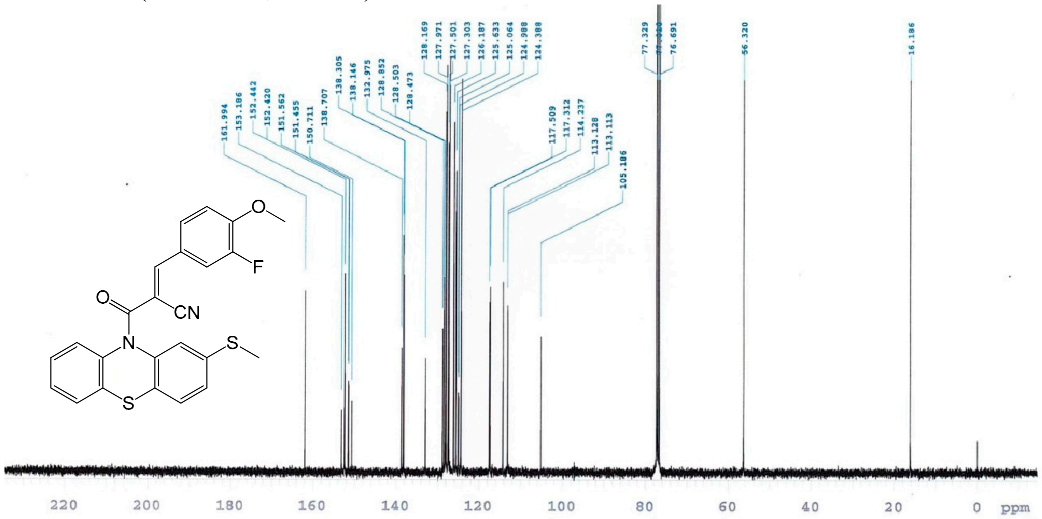

IR-2e

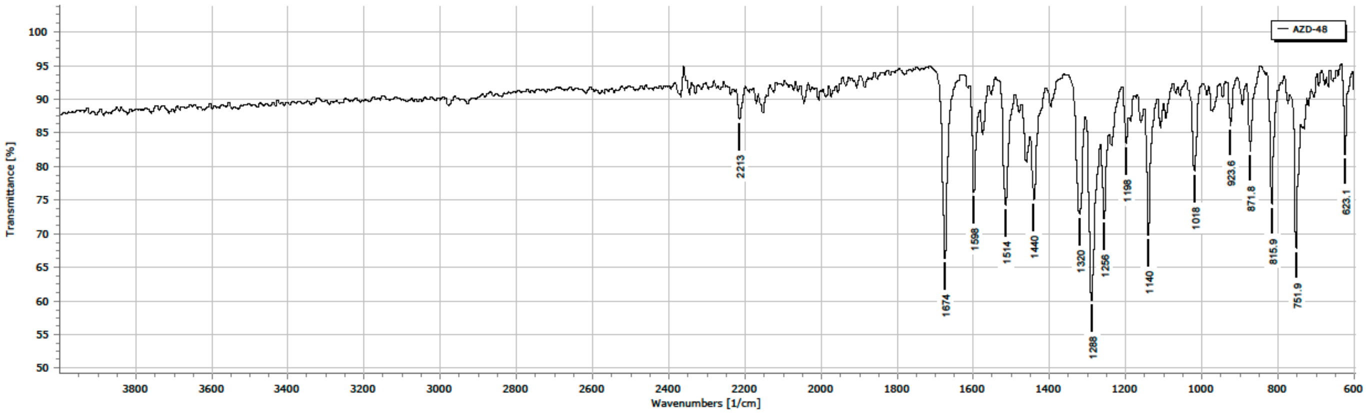

AZD-48
Transmittance [%]
Wavenumbers [1/cm]
2213
1674
1598
1514
1440
1320
1288
1256
1198
1140
1018
923.6
871.8
815.9
751.9
623.1

^{1}H NMR (400 MHz, $CDCl_3$)-**2f**

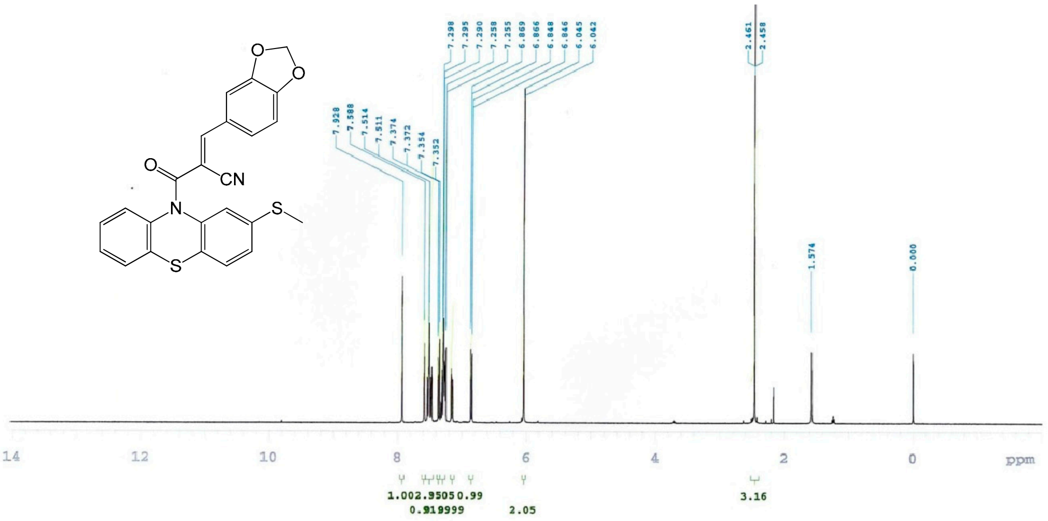

^{13}C NMR (100 MHz, $CDCl_3$)-**2f**

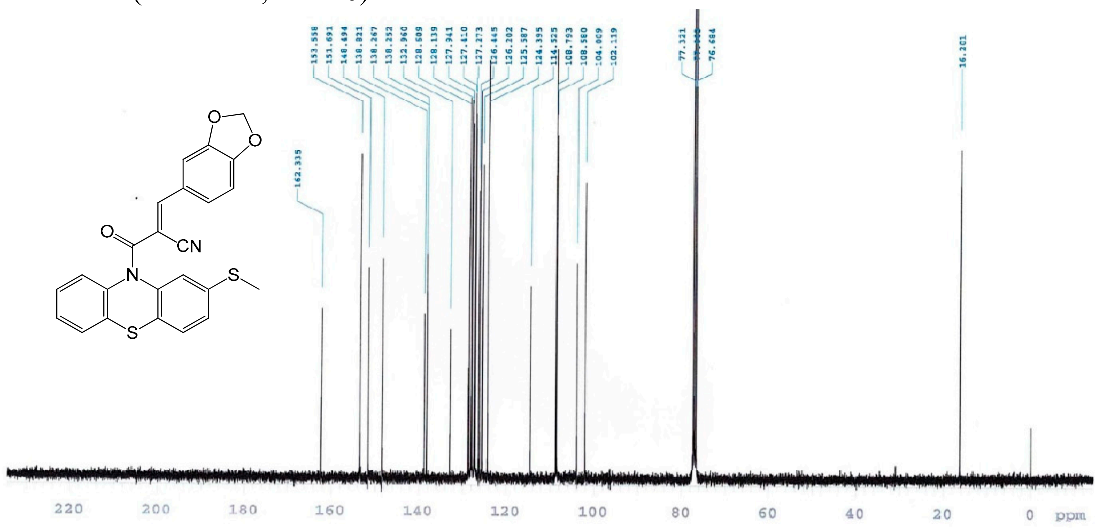

IR-2f

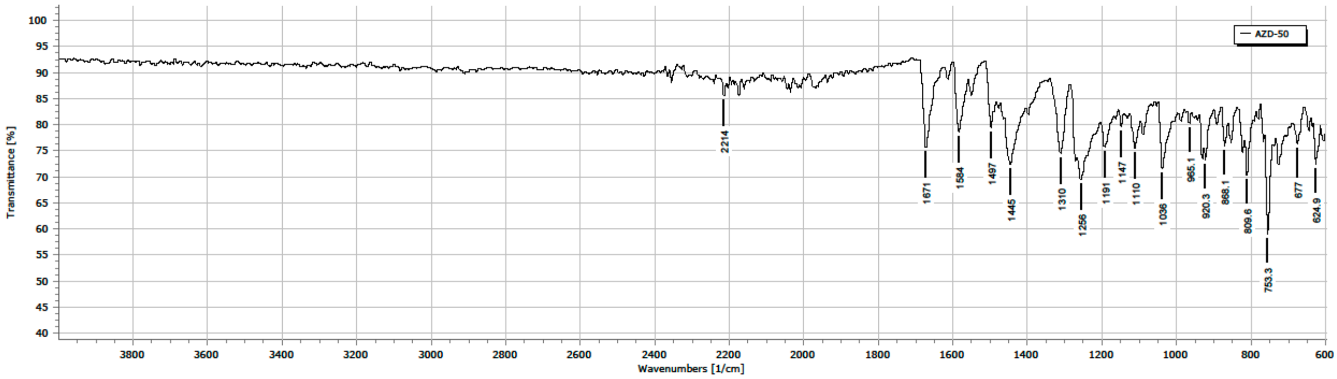

^{1}**H NMR** (500 MHz, $CDCl_3$)- **2g**

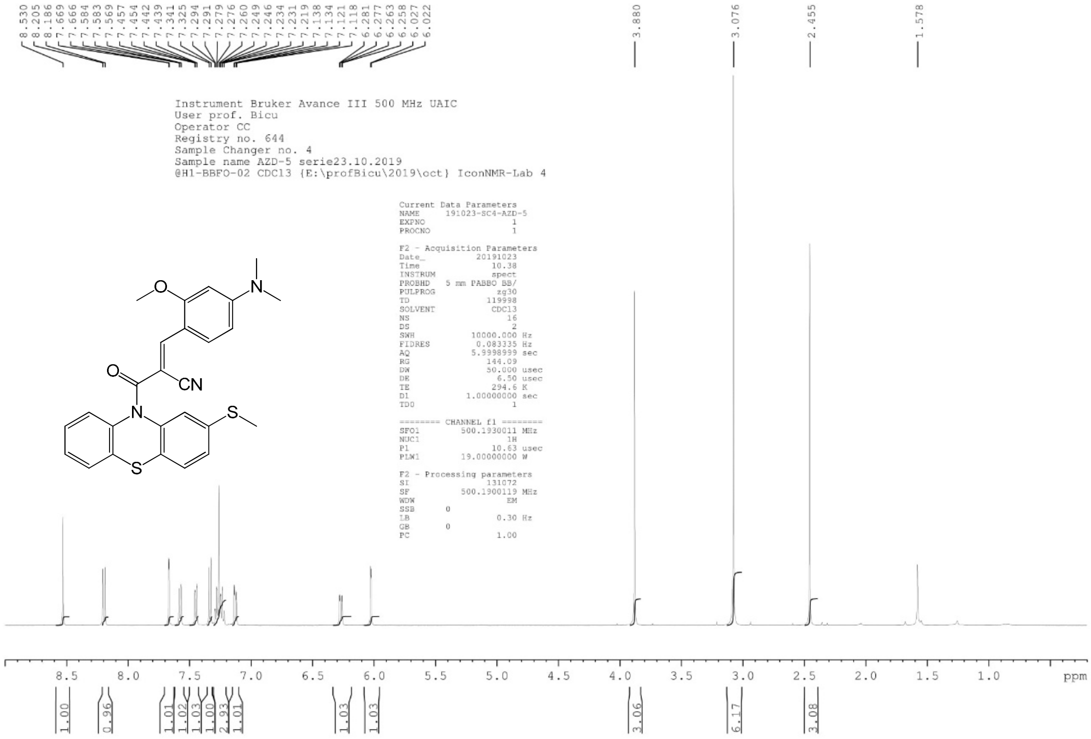

^{13}C NMR (125 MHz, $CDCl_3$)-**2g**

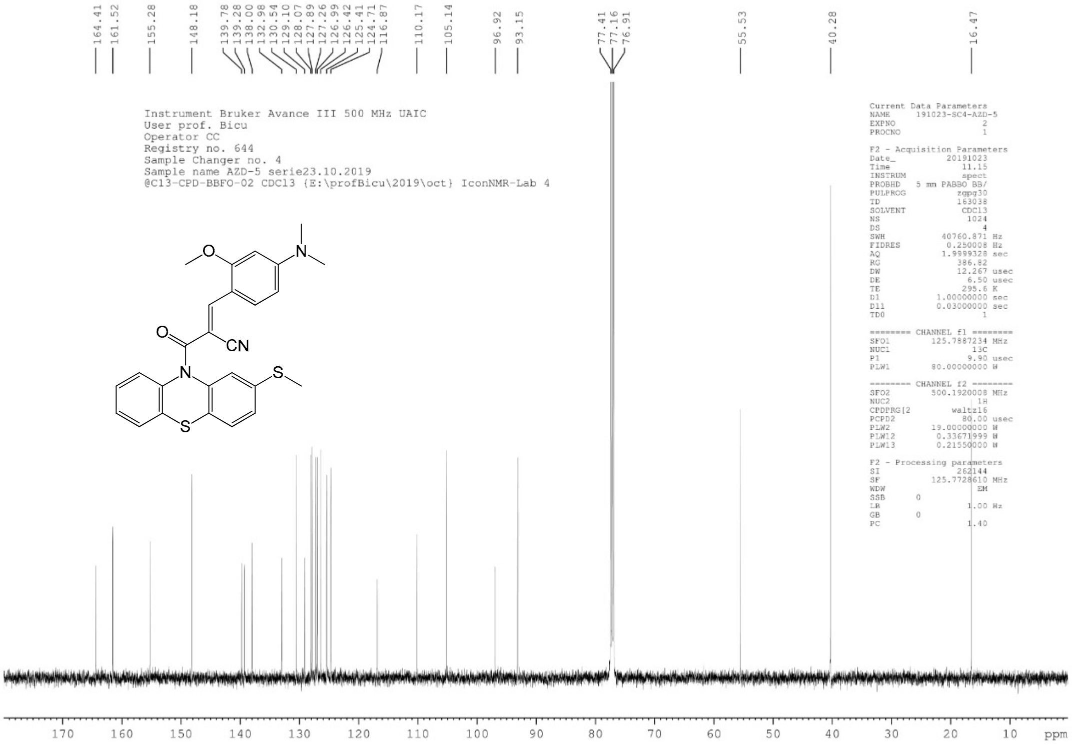

IR-2g

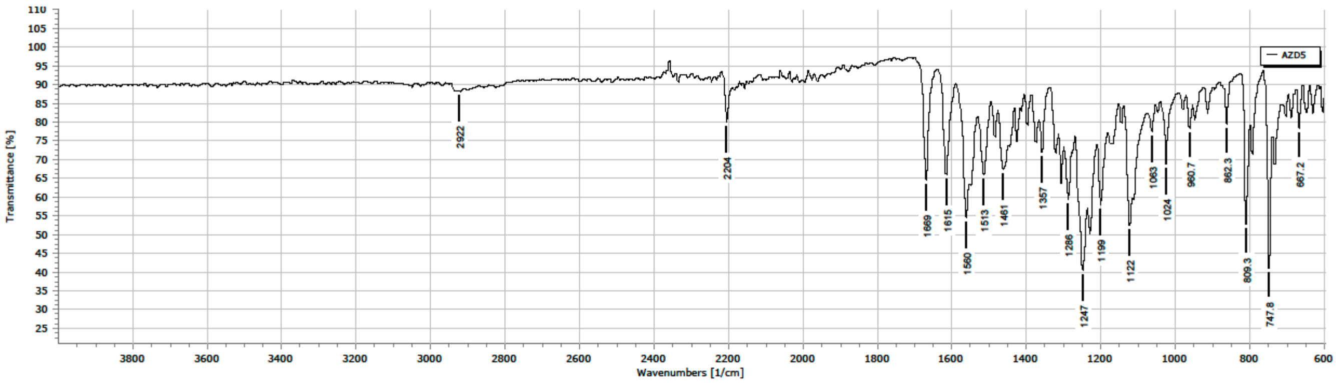

^{1}H NMR (500 MHz, $CDCl_3$)-**2h**

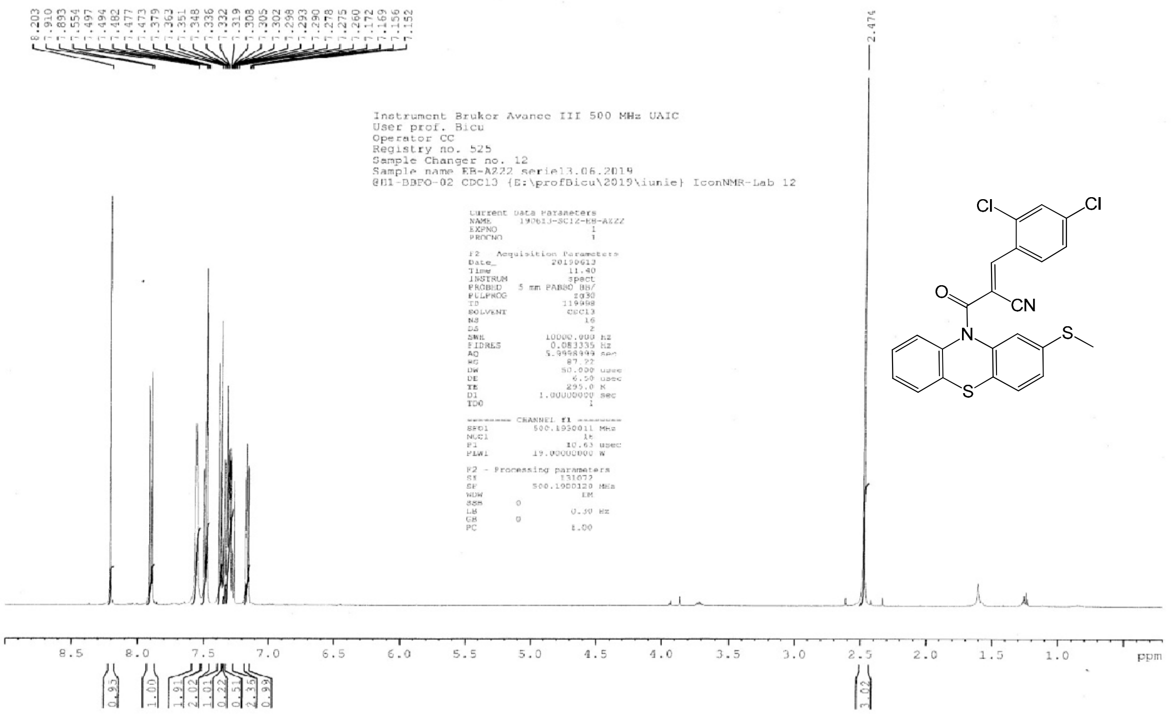

^{13}C NMR (125 MHz, $CDCl_3$)-**2h**

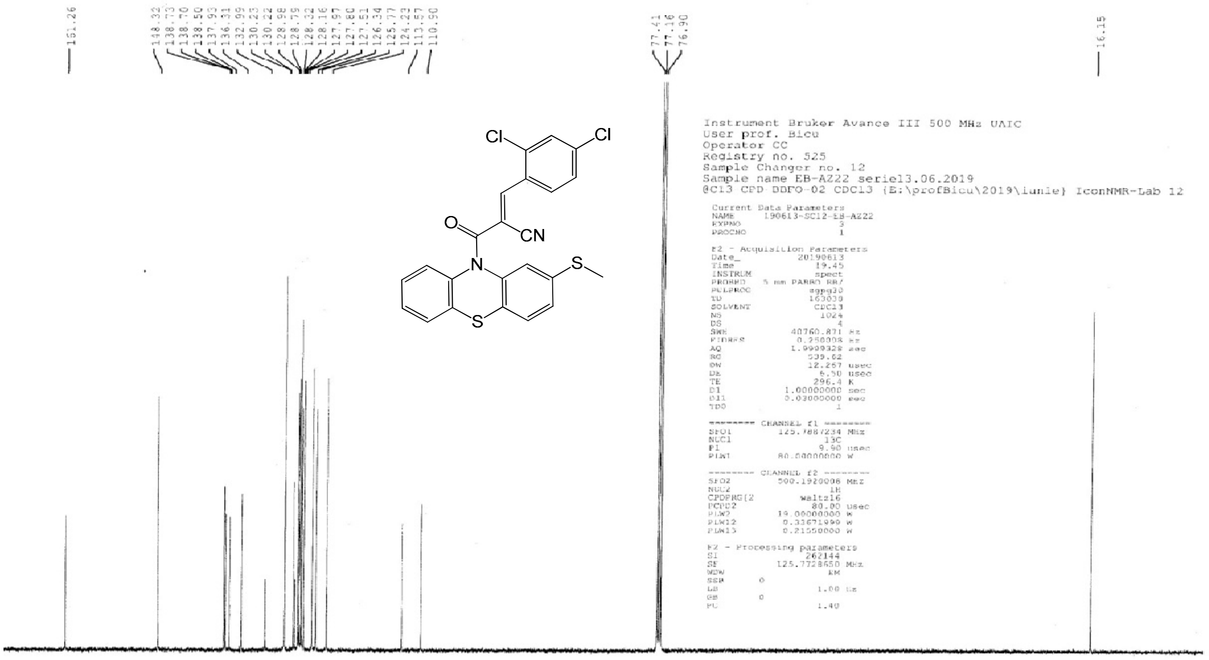

IR-2h

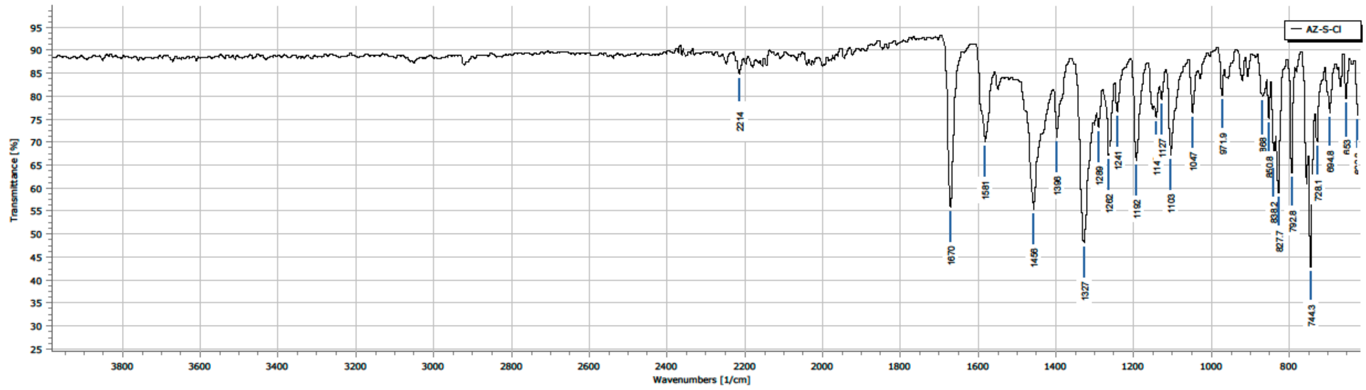

^{1}**H NMR** (500 MHz, $CDCl_3$)-**2i**

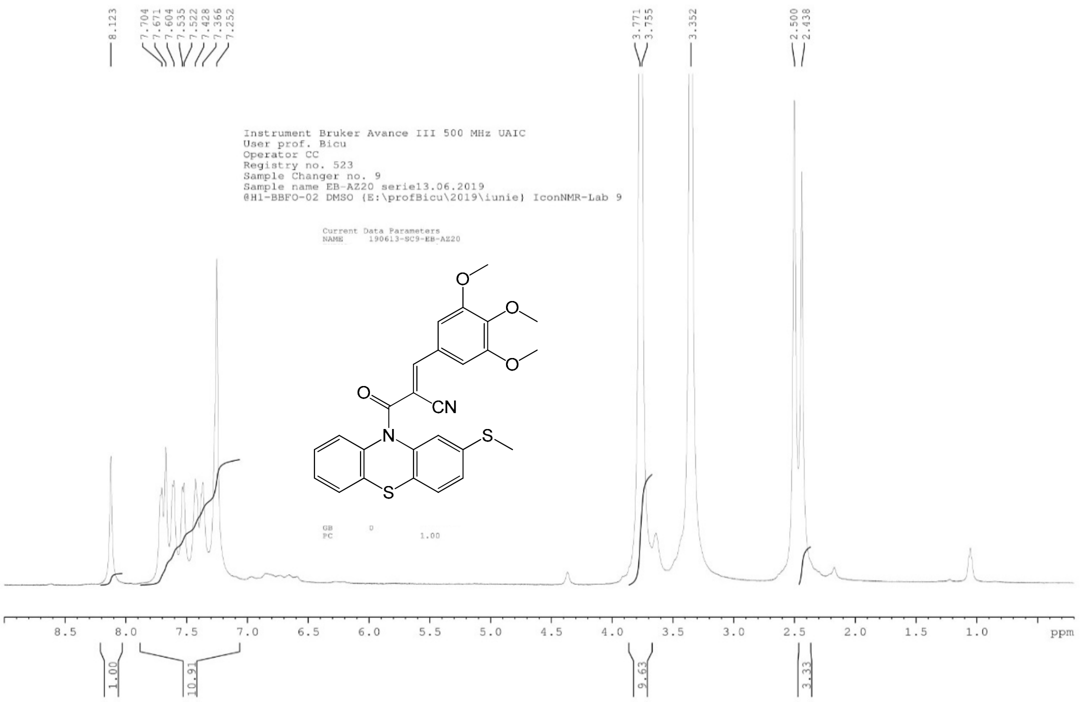

^{13}C NMR (125 MHz, $CDCl_3$)-**2i**

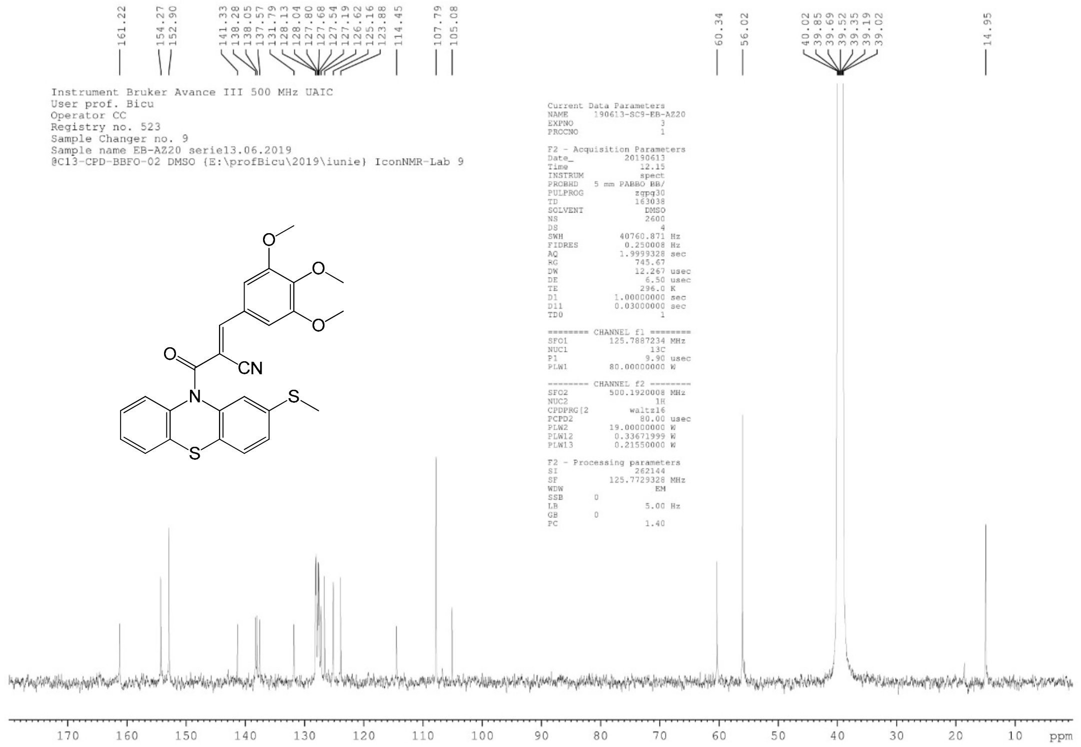

IR-2i

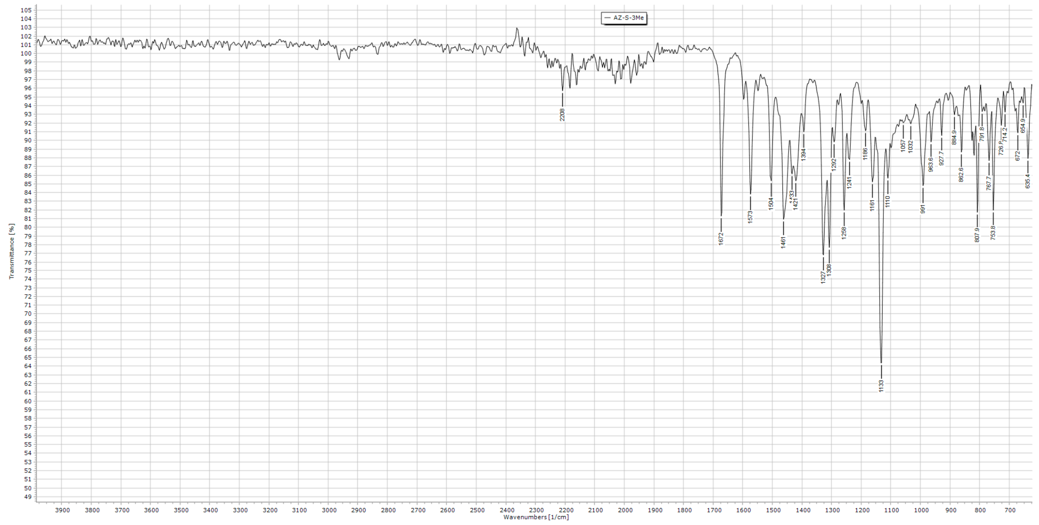

^{1}H NMR (400 MHz, $CDCl_3$)-**2j**

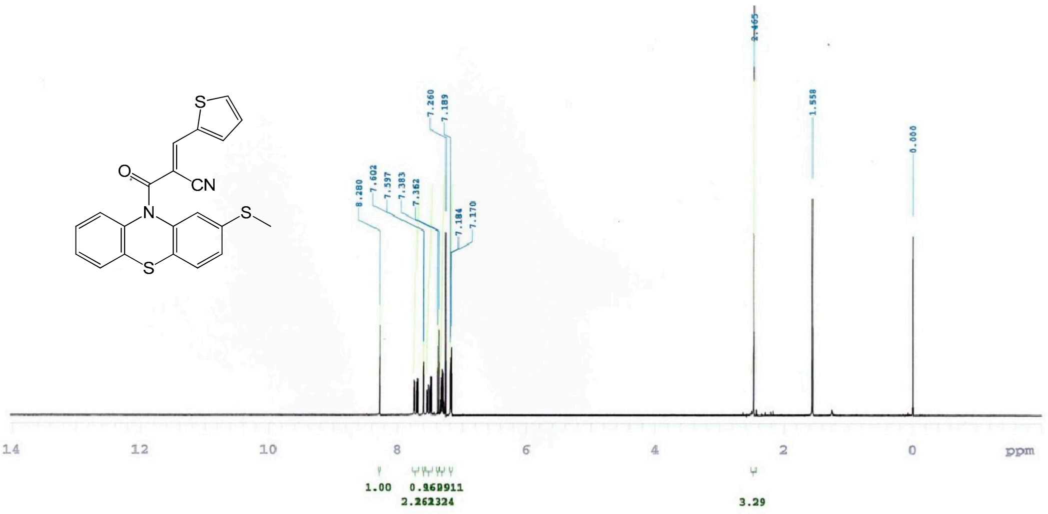

^{13}C NMR (100 MHz, $CDCl_3$)-**2j**

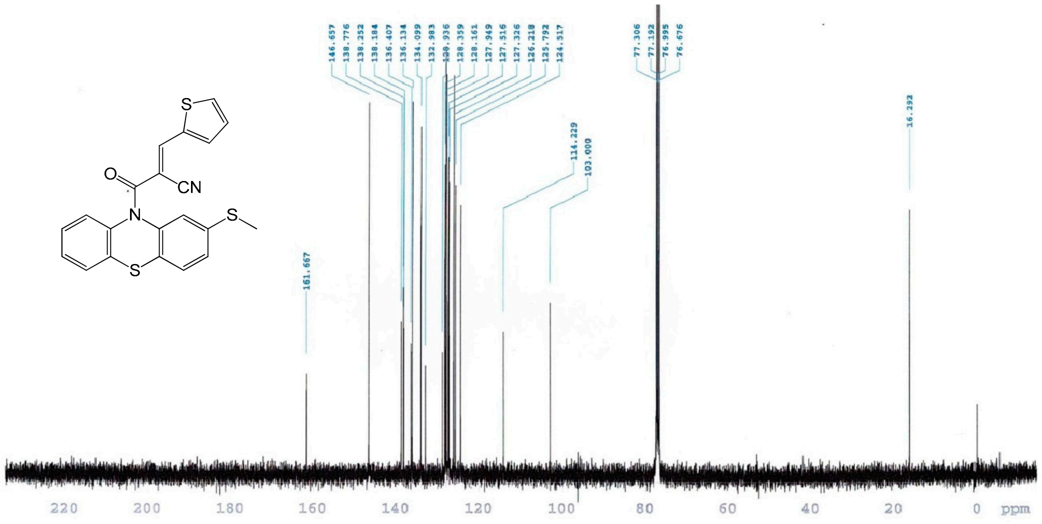

IR-2j

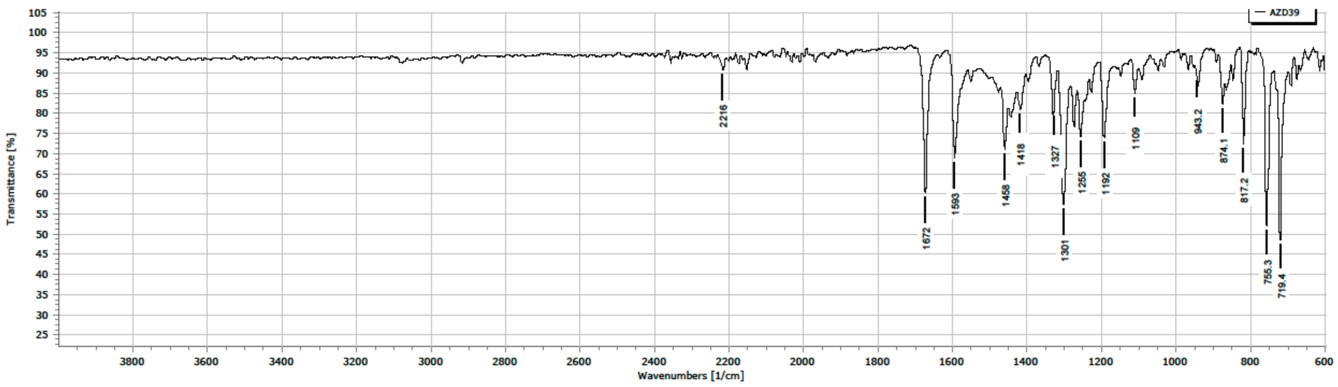

^{1}H NMR (500 MHz, DMSO-d_6)- **2k**

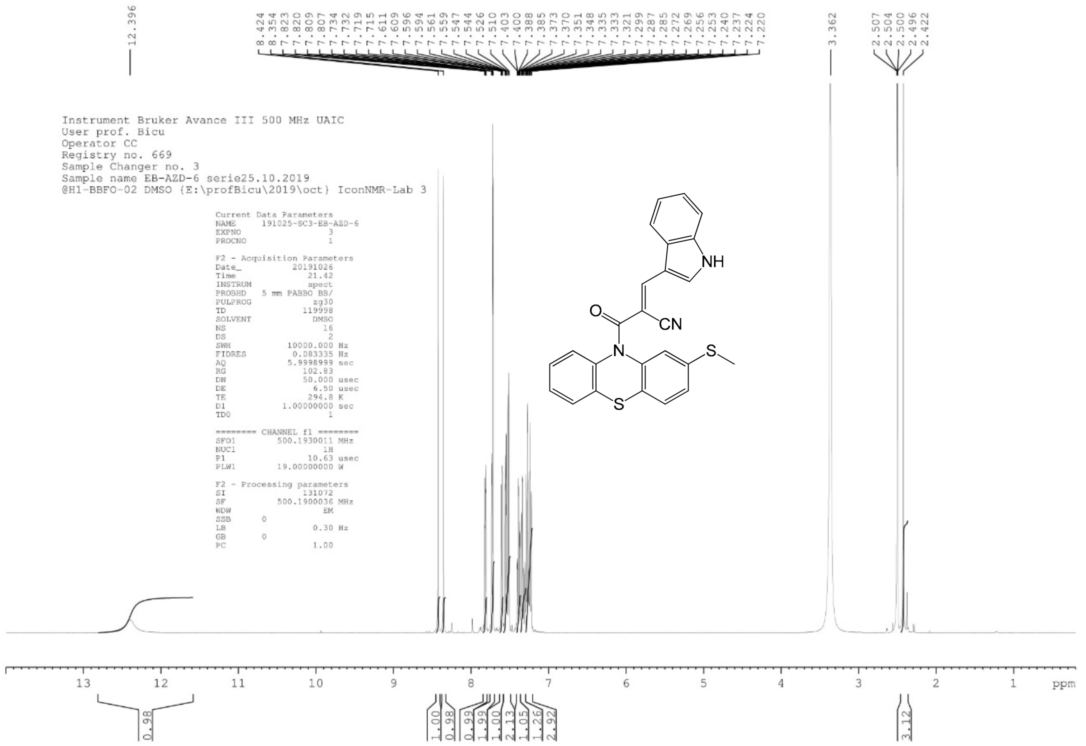

^{13}C NMR (125 MHz, DMSO-d_6)-**2k**

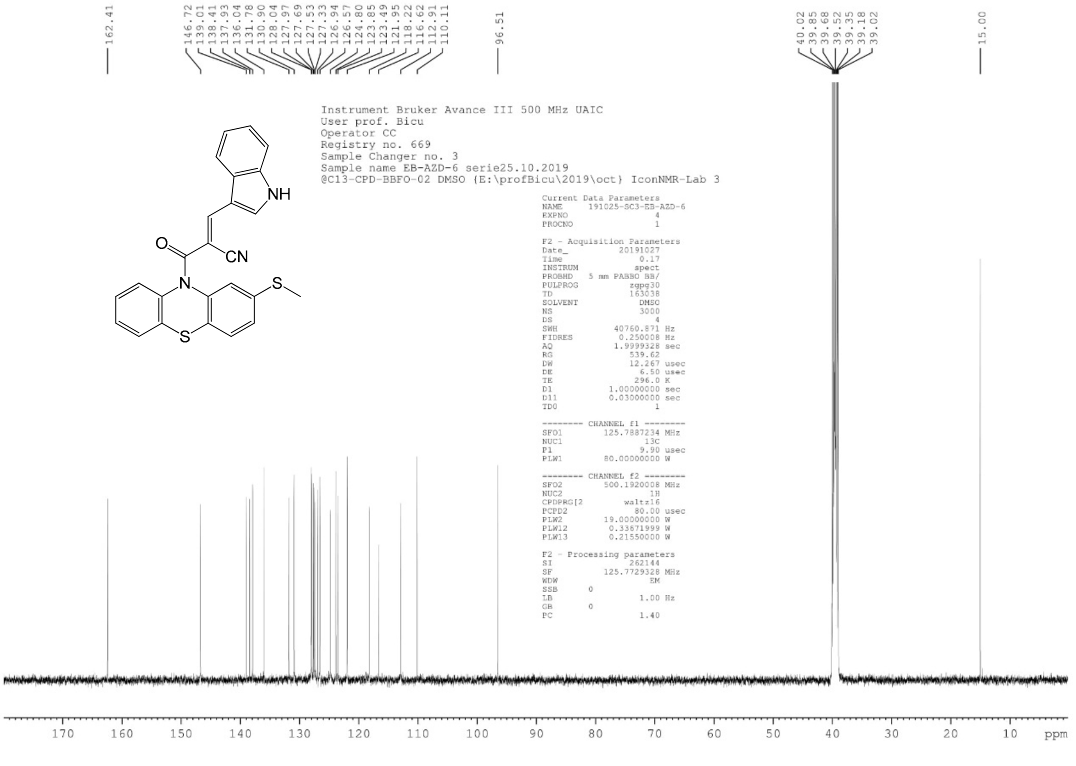

IR-2k

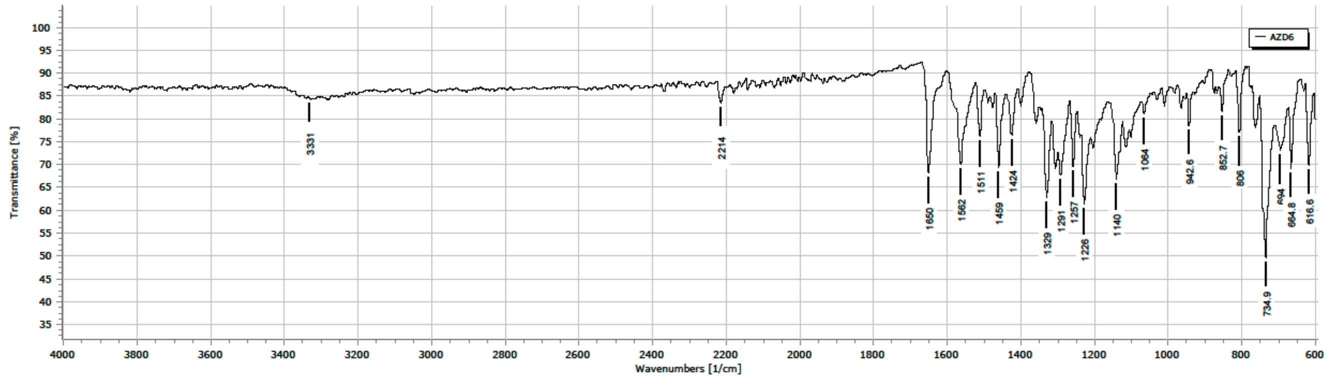

^{1}**H NMR** (500 MHz, DMSO-d_6)- **2l**

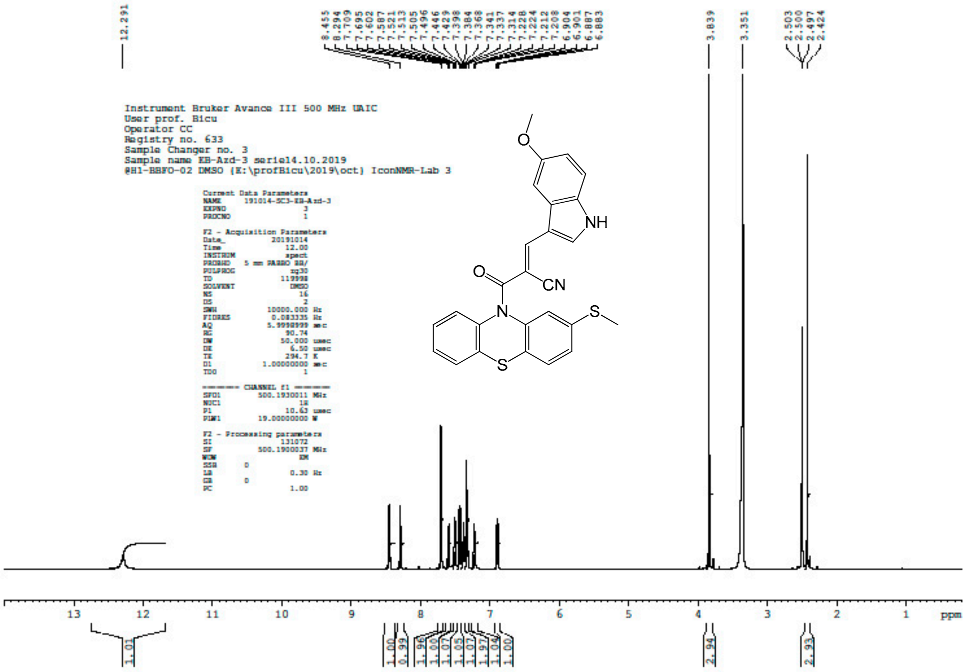

^{13}**C NMR** (125 MHz, DMSO-*d*$_6$)-**2l**

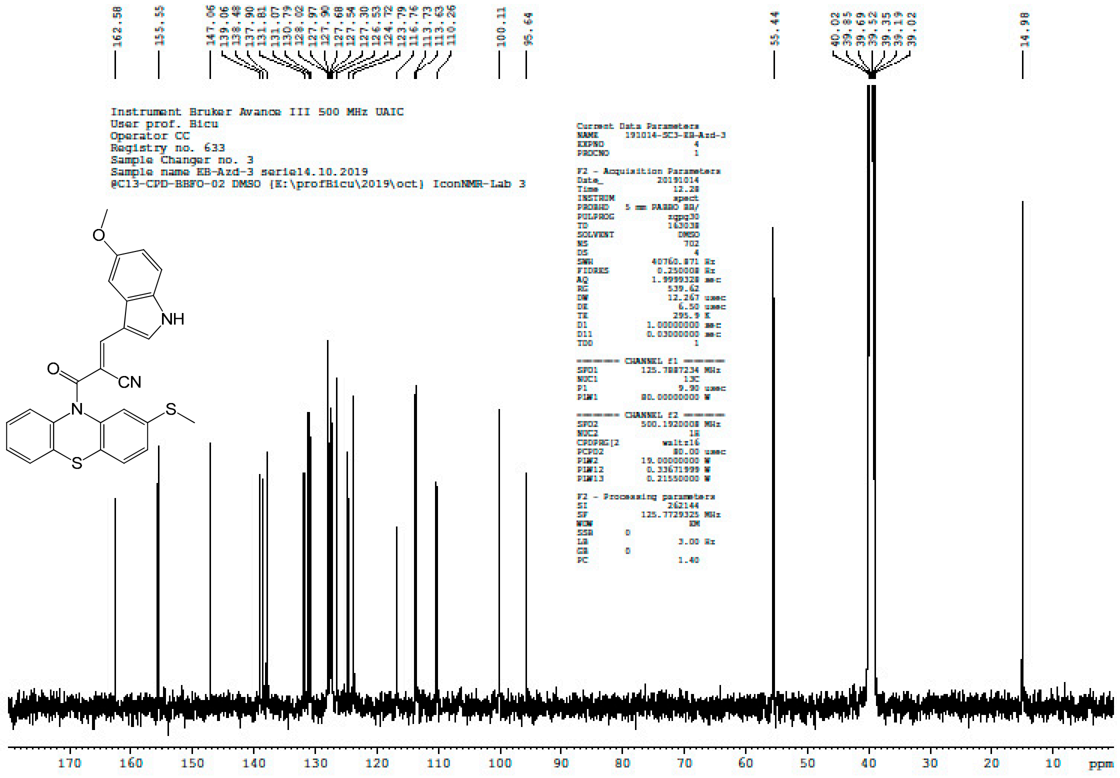

^{13}C-DEPT NMR (125 MHz, DMSO-*d*$_6$)-**2l**

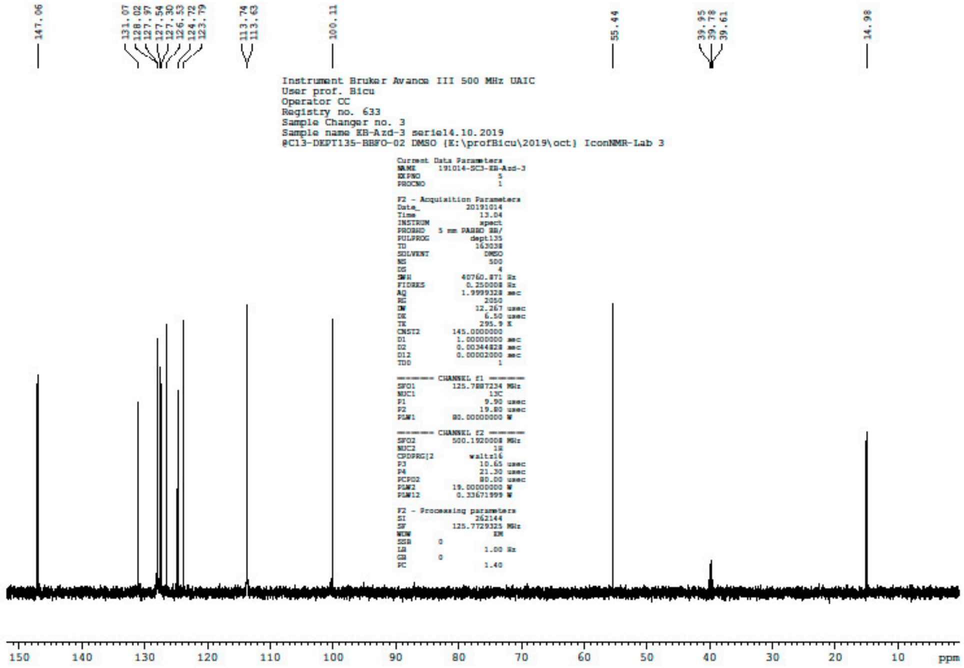

HMQC NMR (500 MHz, DMSO-*d*$_6$)-**2l**

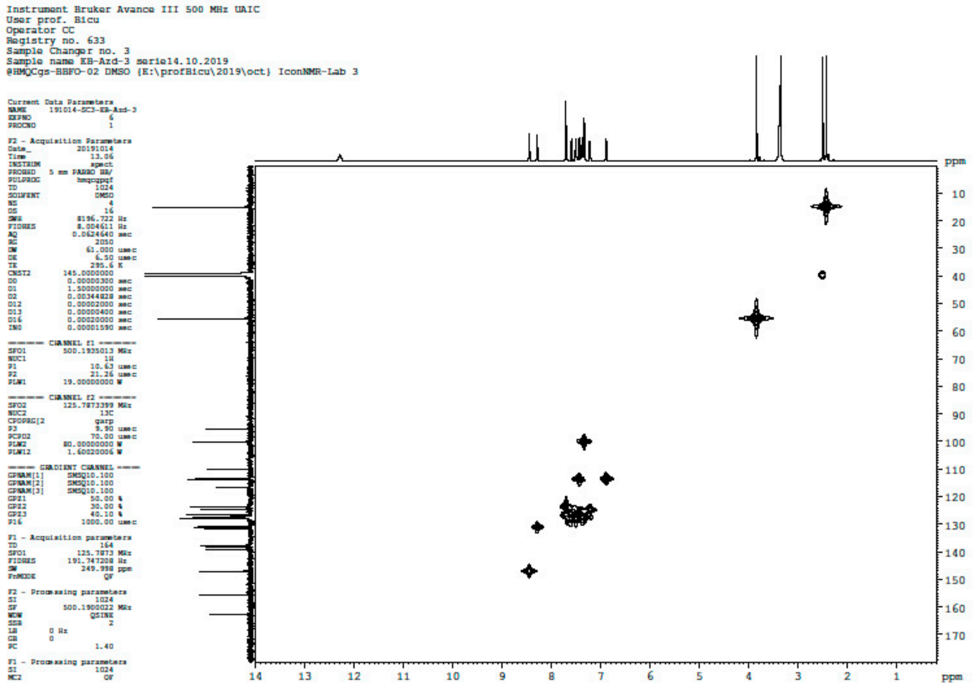

IR-2l

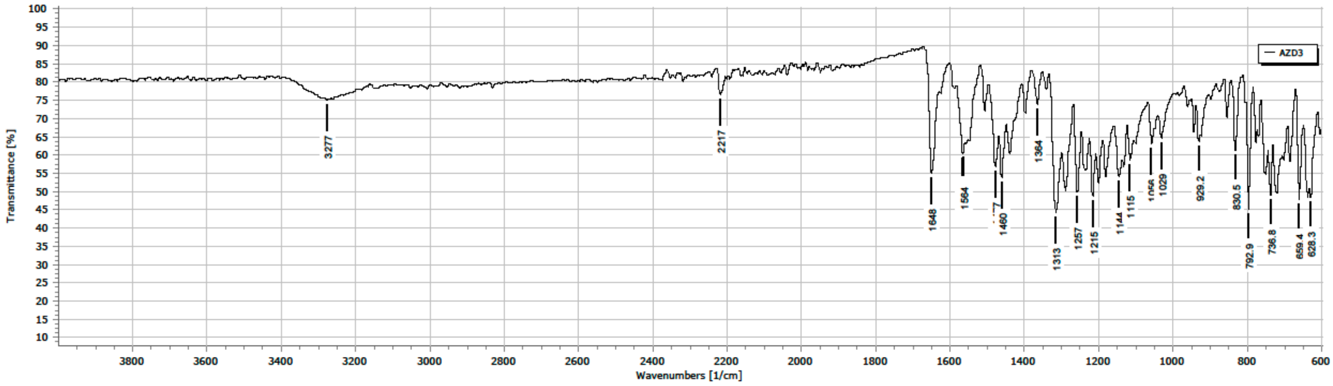

^{1}**H NMR** (500 MHz, DMSO-d_6)- **2m**

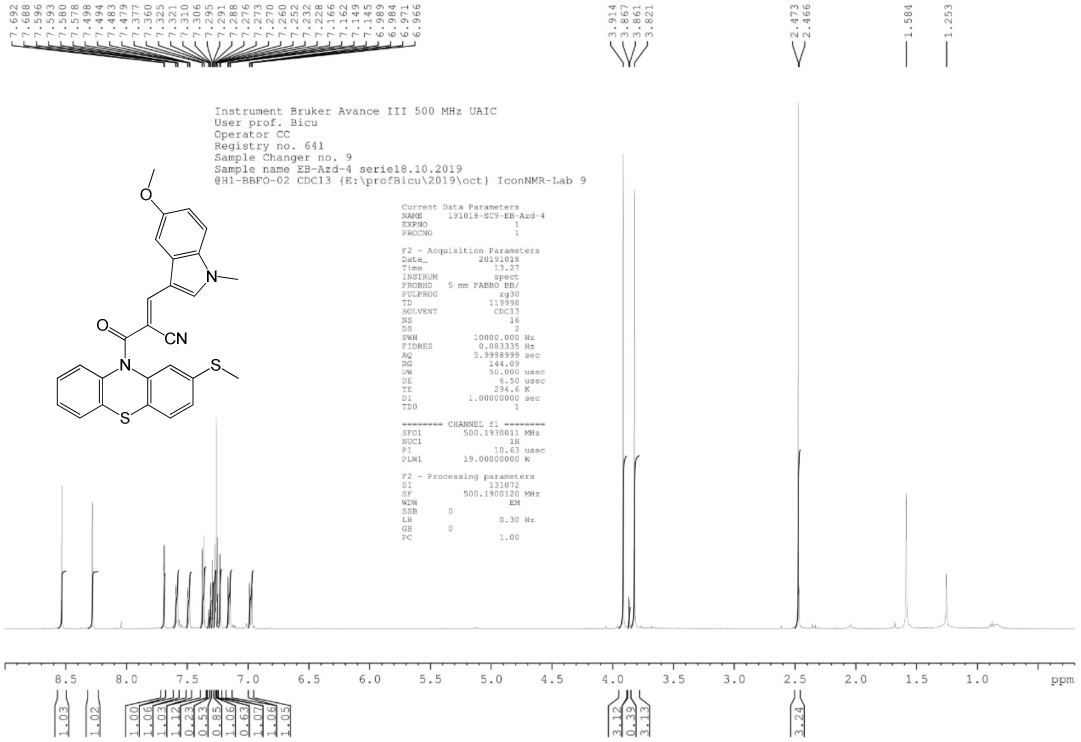

^{13}C NMR (125 MHz, DMSO-*d*$_6$)-**2m**

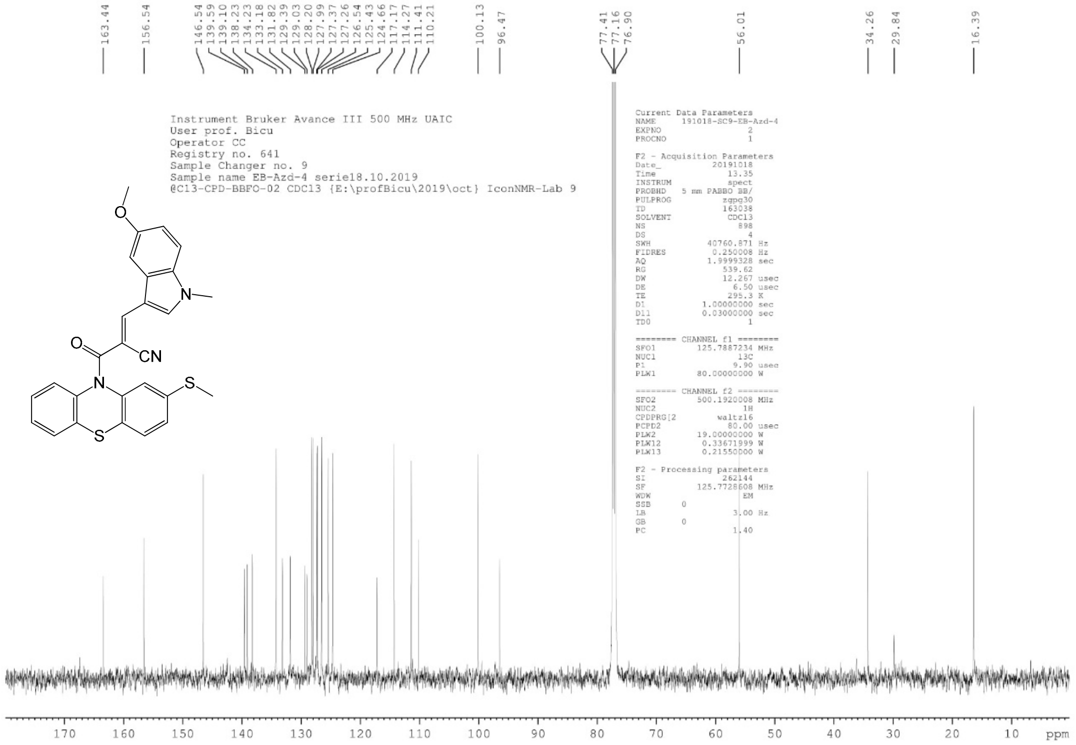

IR-2m

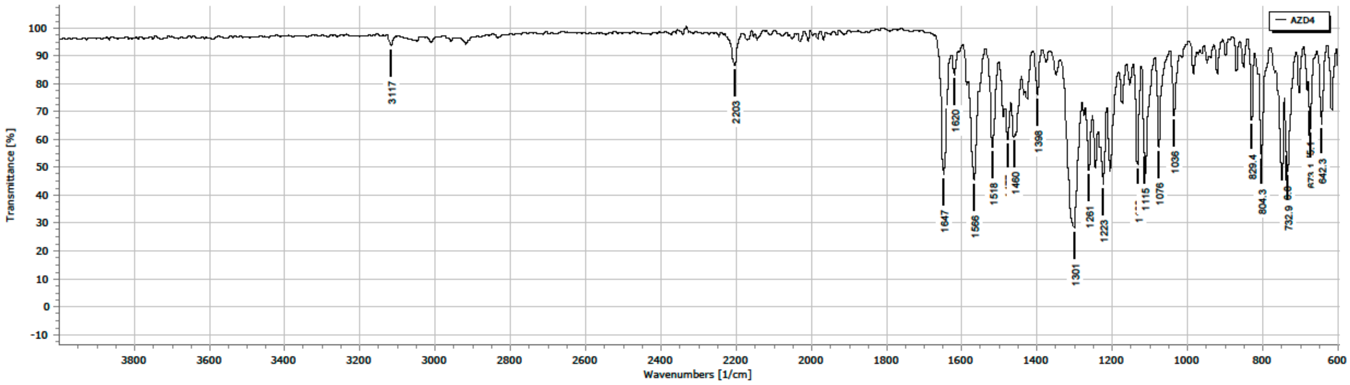

^{1}H NMR (500 MHz, $CDCl_3$)- **2n**

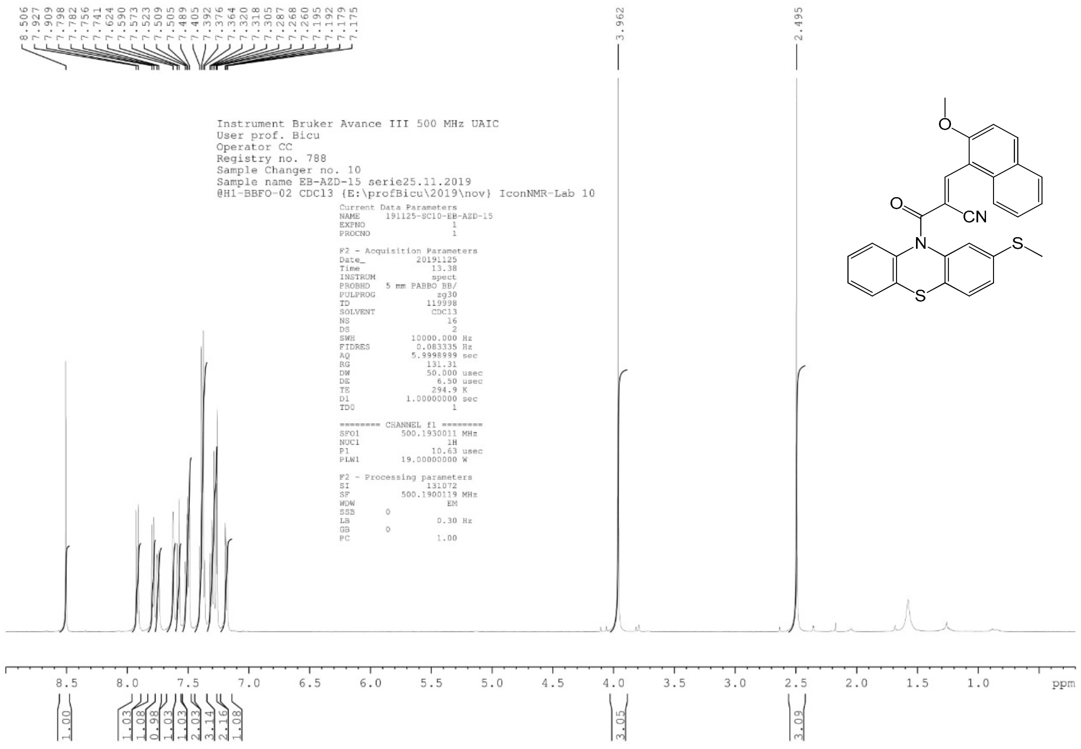

^{13}C NMR (125 MHz, $CDCl_3$)-**2n**

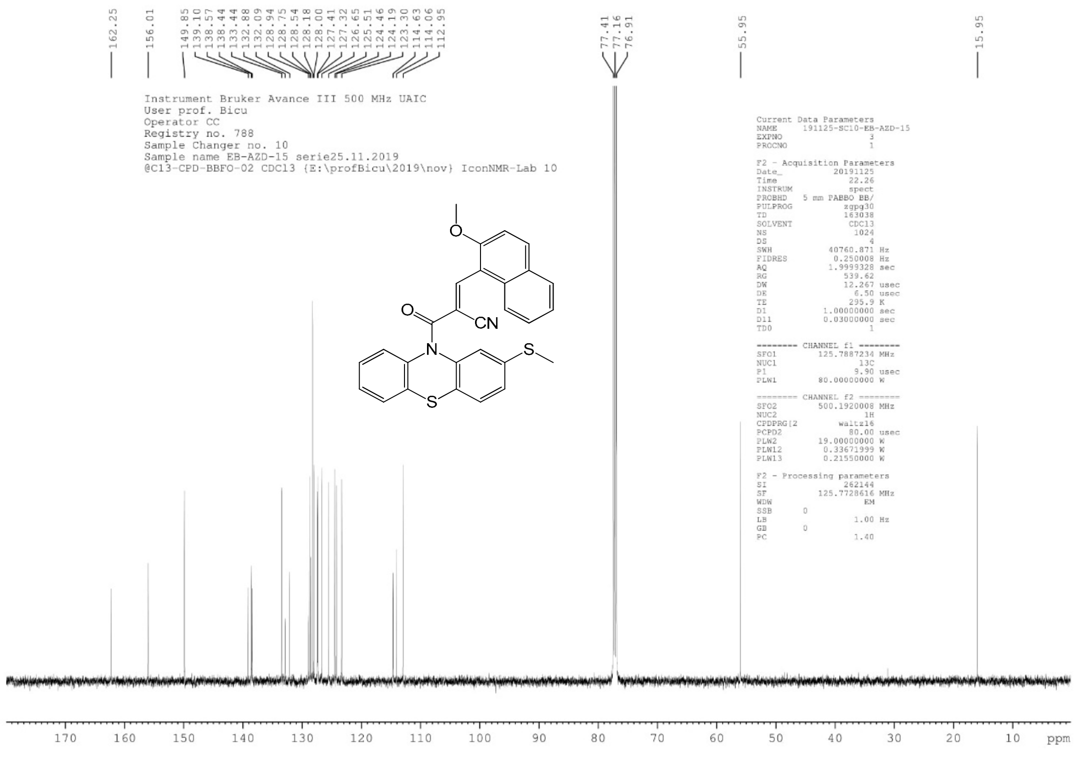

IR-2n

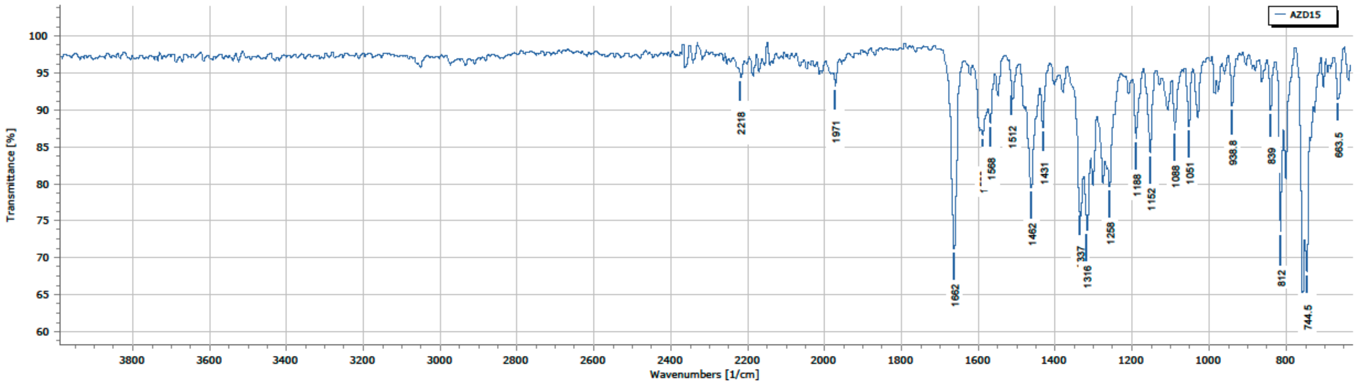

AZD15
Transmittance [%]
Wavenumbers [1/cm]
2218
1971
1662
1568
1512
1462
1431
1316
1258
1188
1152
1088
1051
938.8
839
812
744.5
663.5

^{1}H NMR (400 MHz, $CDCl_3$)-**2o**

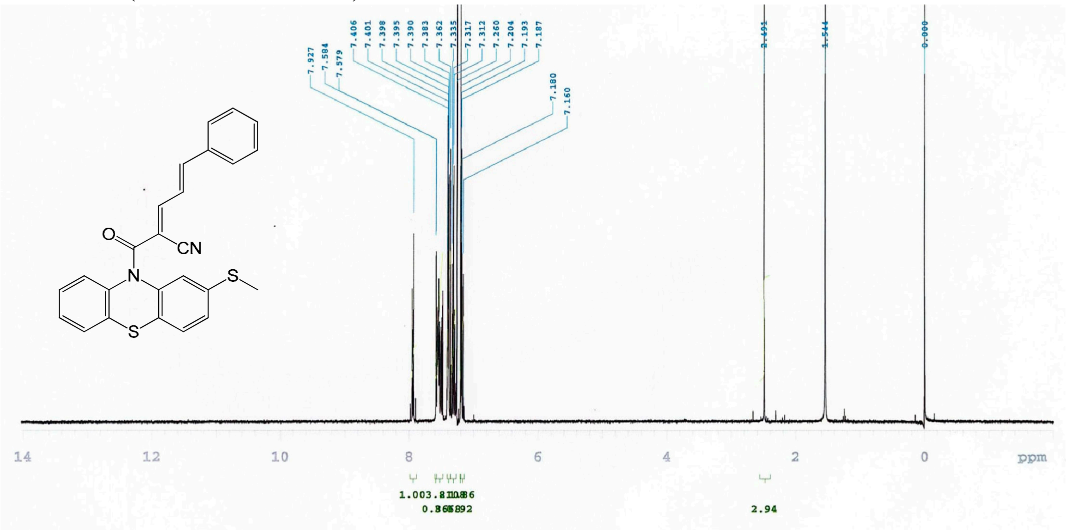

^{13}C NMR (100 MHz, $CDCl_3$)-**2o**

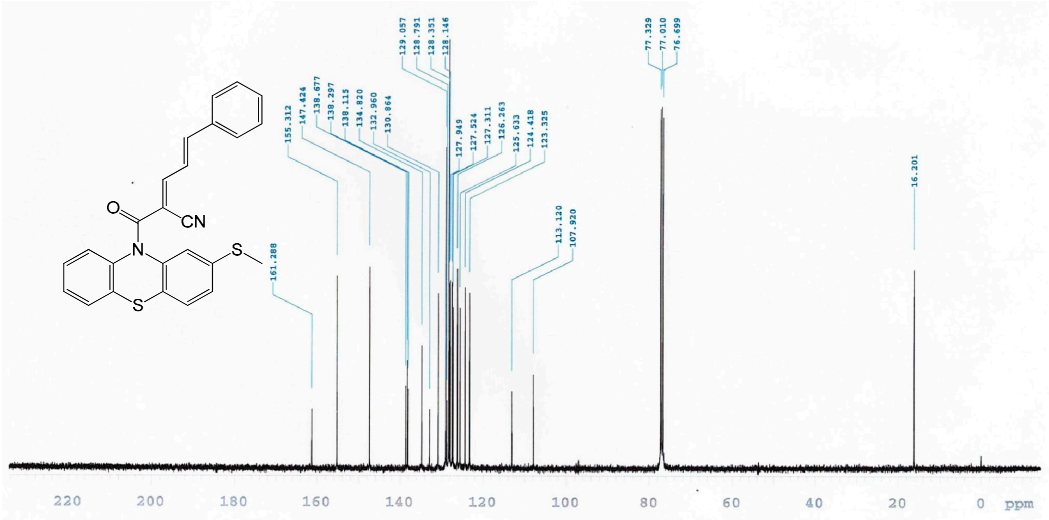

IR-2o

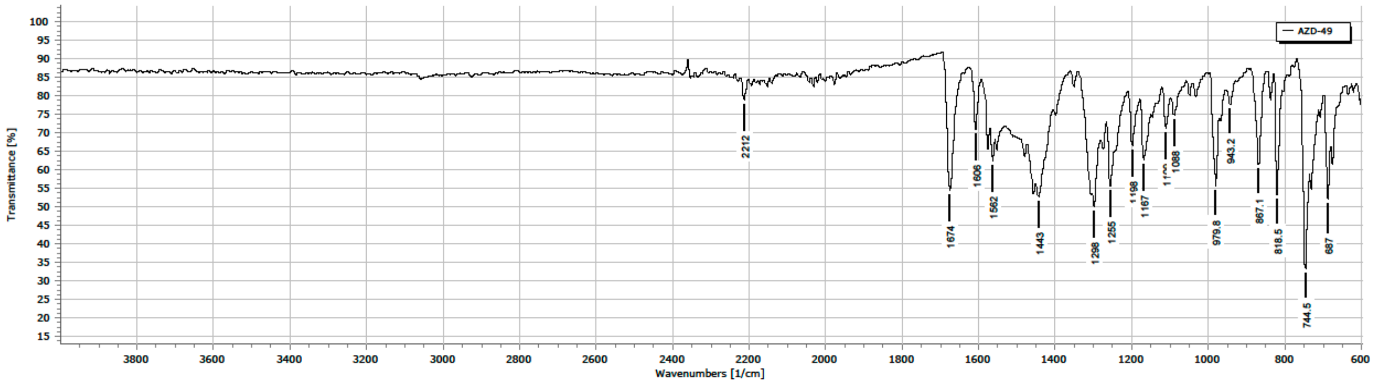

^{1}H NMR (500 MHz, $CDCl_3$)- **3a**

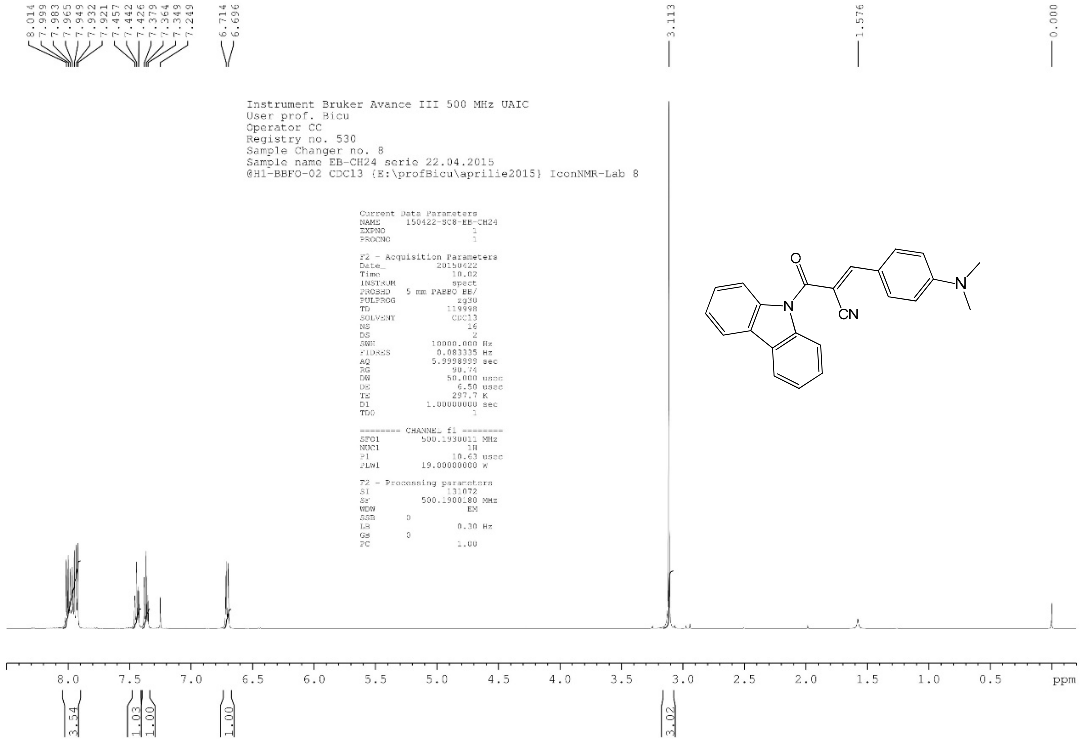

^{13}C NMR (125 MHz, $CDCl_3$)-**3a**

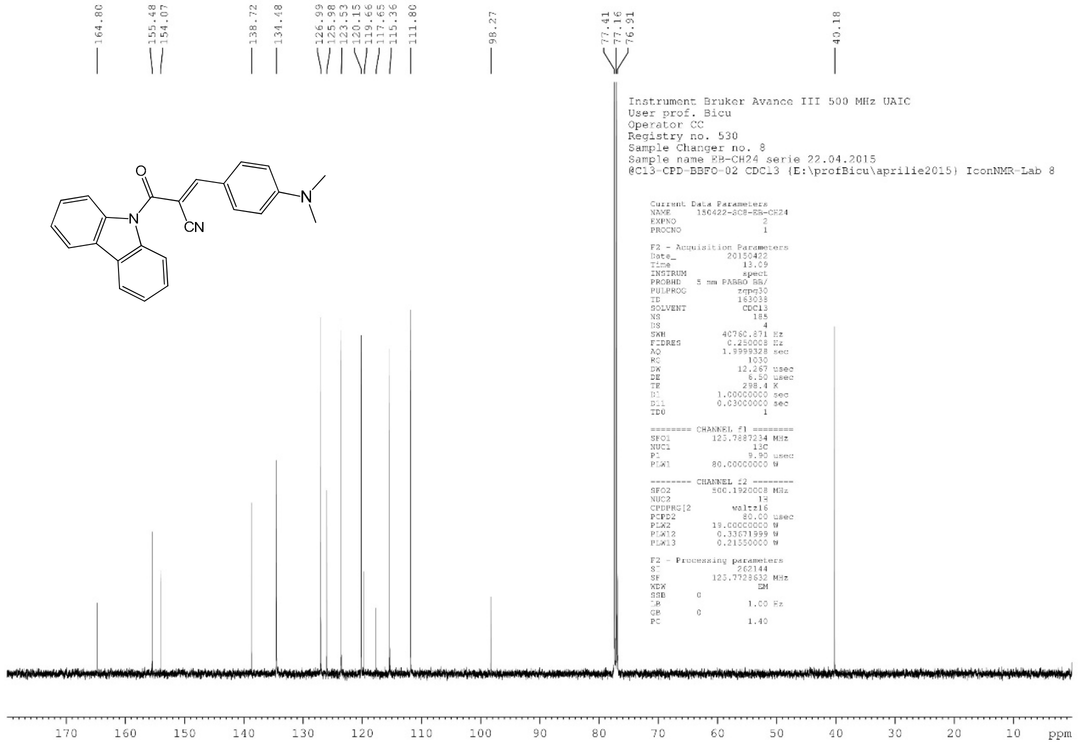

IR-3a

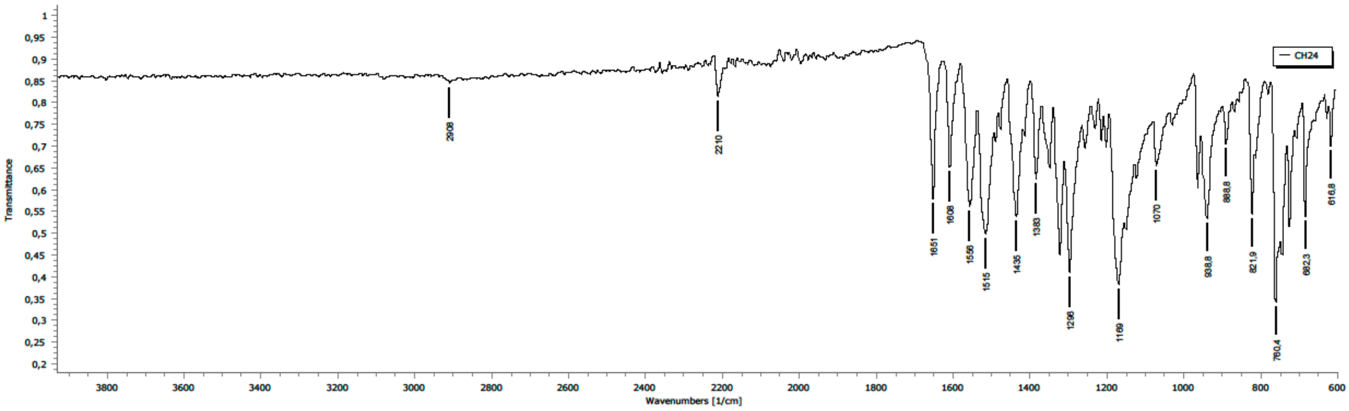

^{1}H NMR (500 MHz, $CDCl_3$)-**3b**

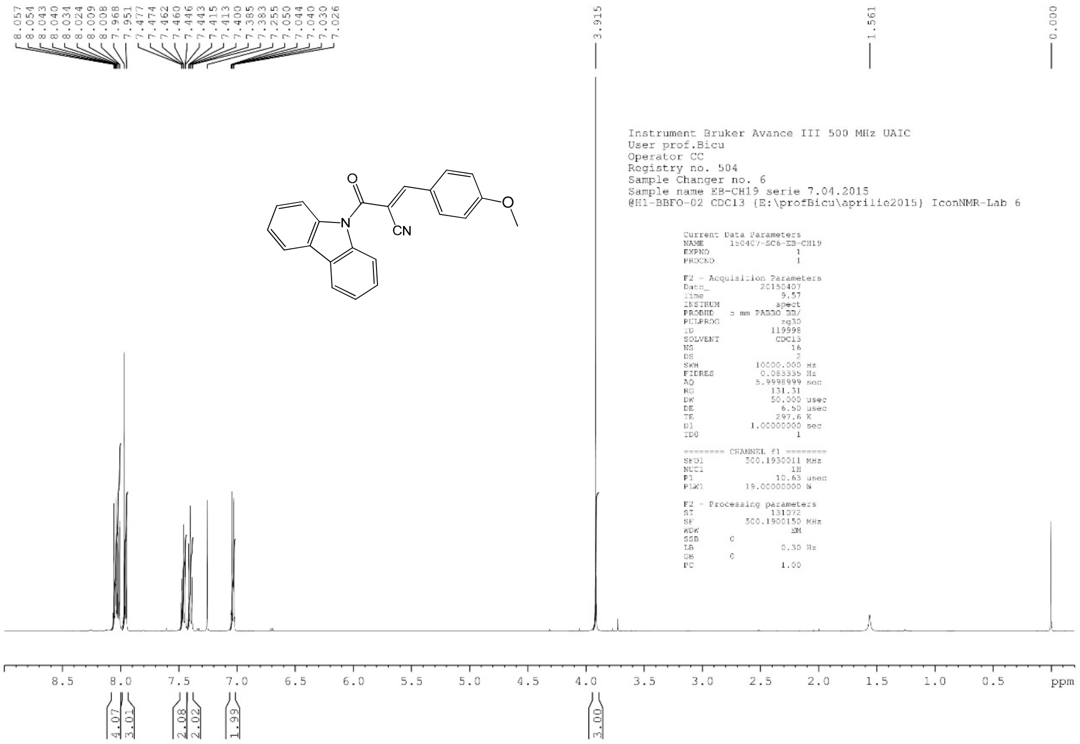

^{13}C NMR (125 MHz, $CDCl_3$)-**3b**

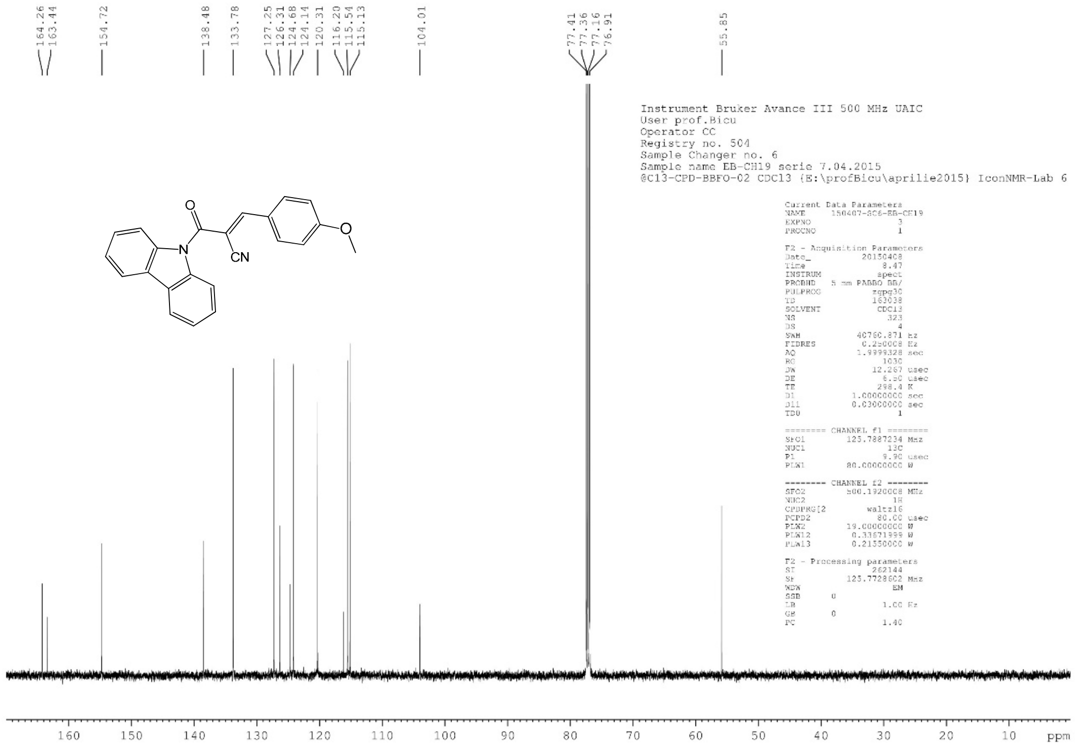

IR-3b

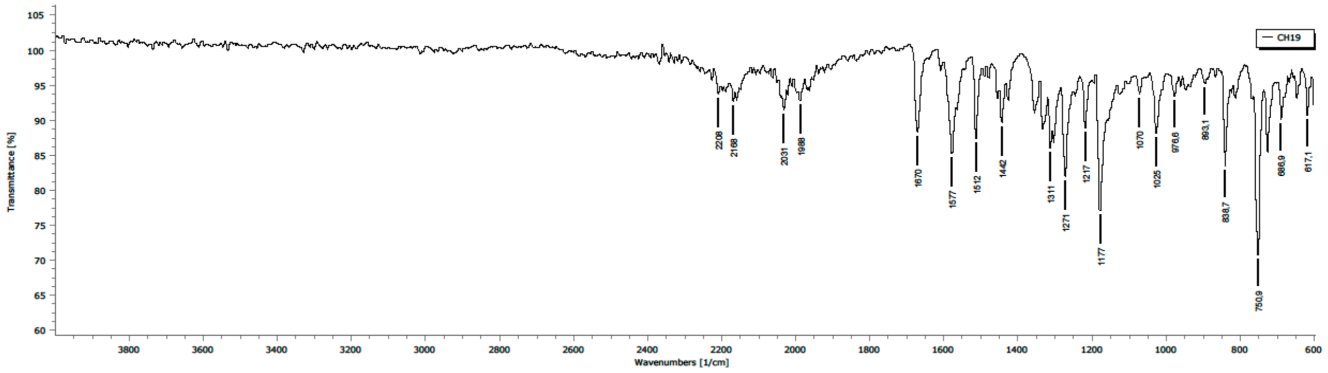

^{1}H NMR (500 MHz, $CDCl_3$)-**3c**

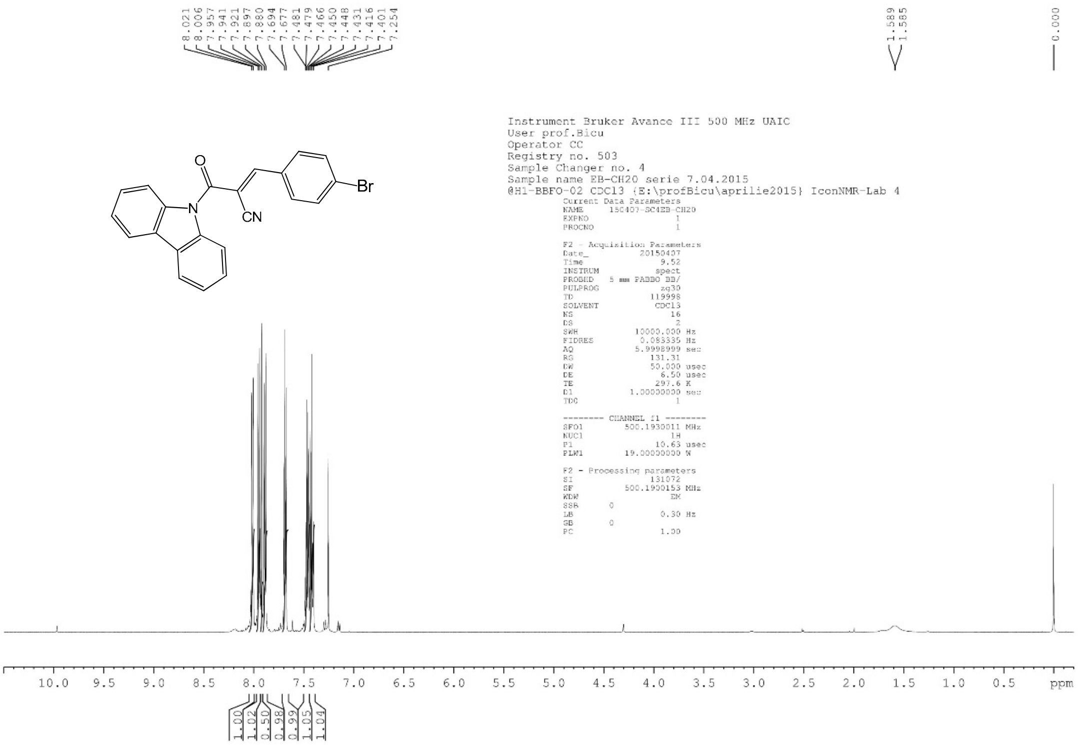

^{13}C NMR (125 MHz, $CDCl_3$)-**3c**

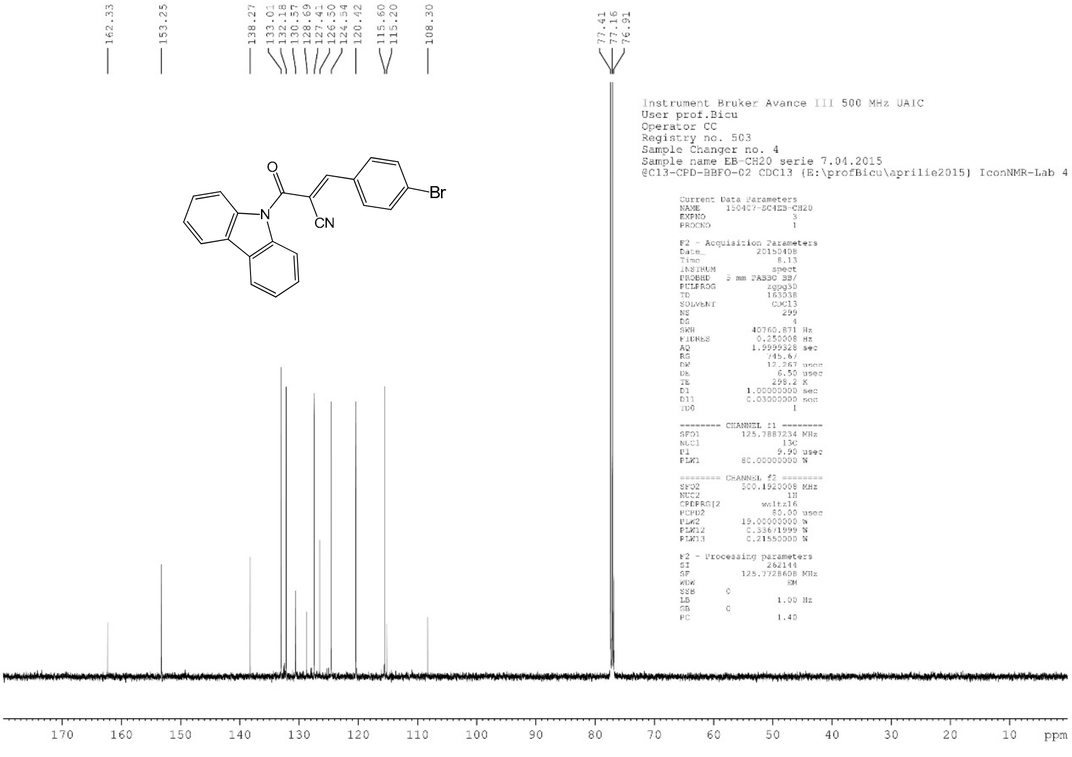

IR-3c

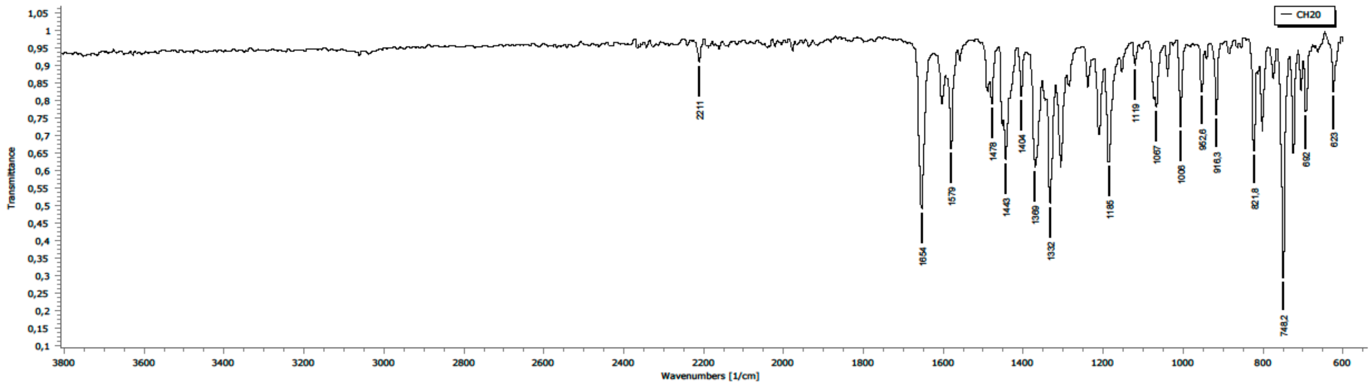

^{1}H NMR (500 MHz, DMSO-d_6)-**3d**

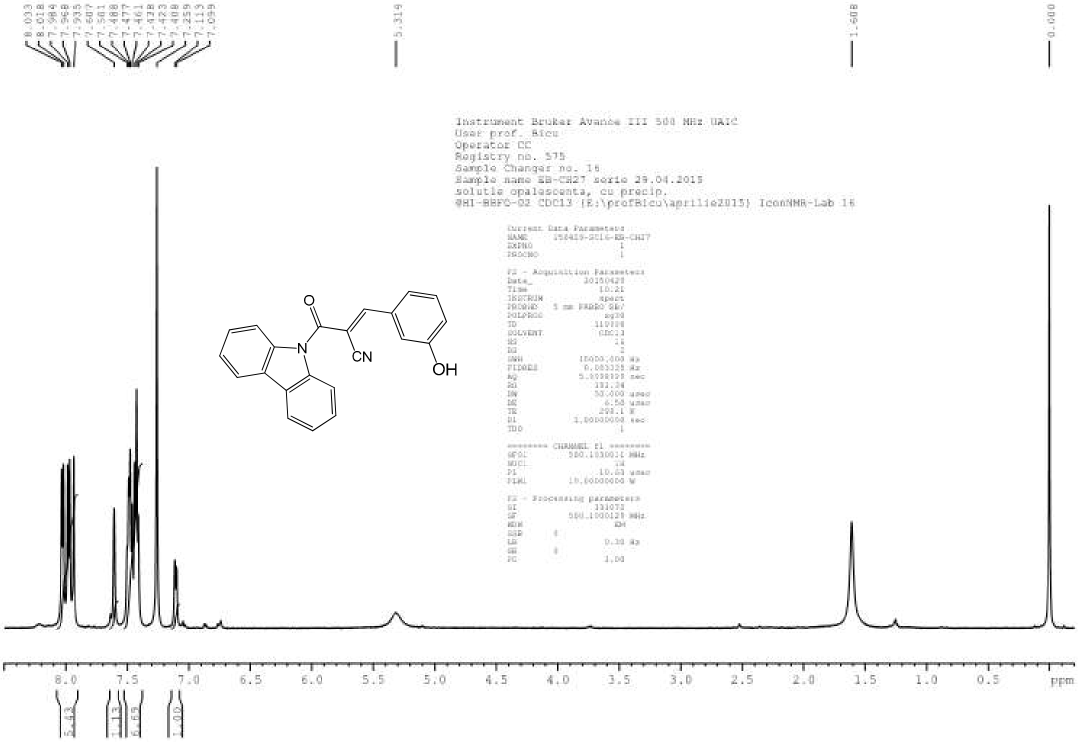

^{13}C NMR (125 MHz, DMSO-d_6)-**3d**

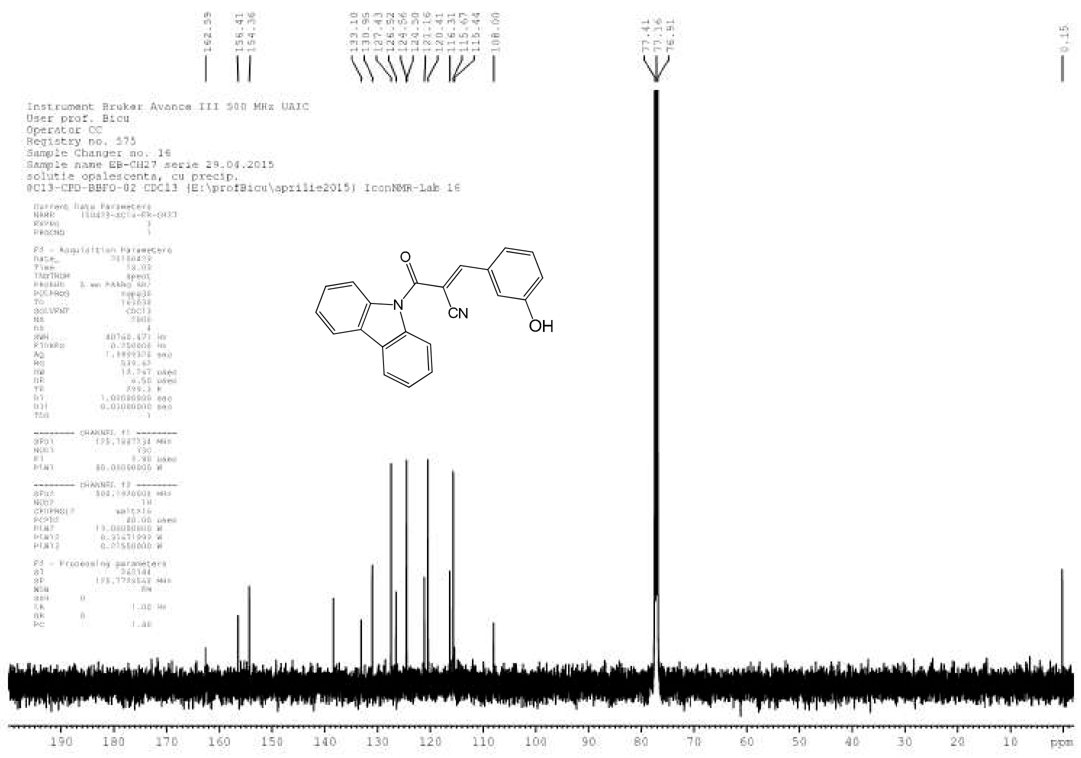

IR-3d

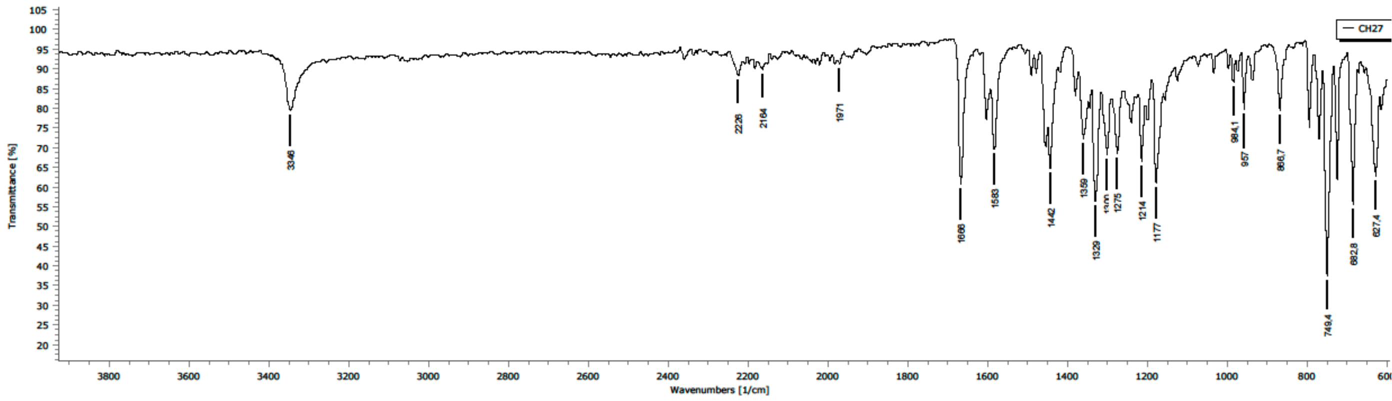

^{1}H NMR (500 MHz, $CDCl_3$)-**3e**

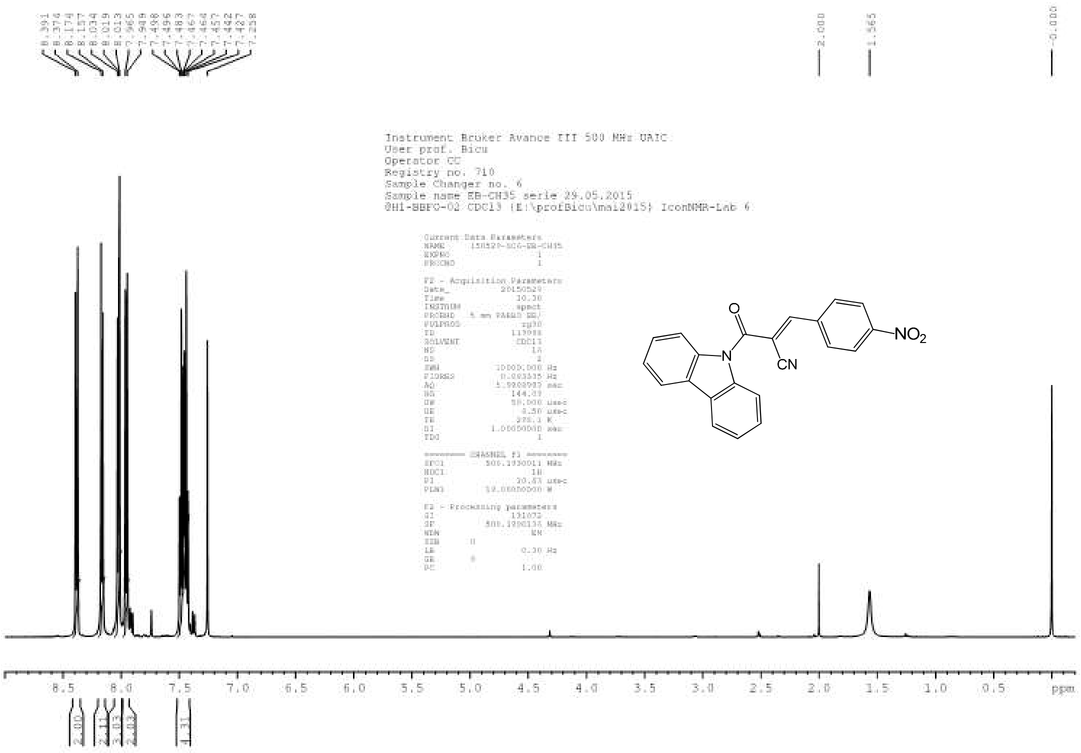

^{13}C NMR (125 MHz, $CDCl_3$)-**3e**

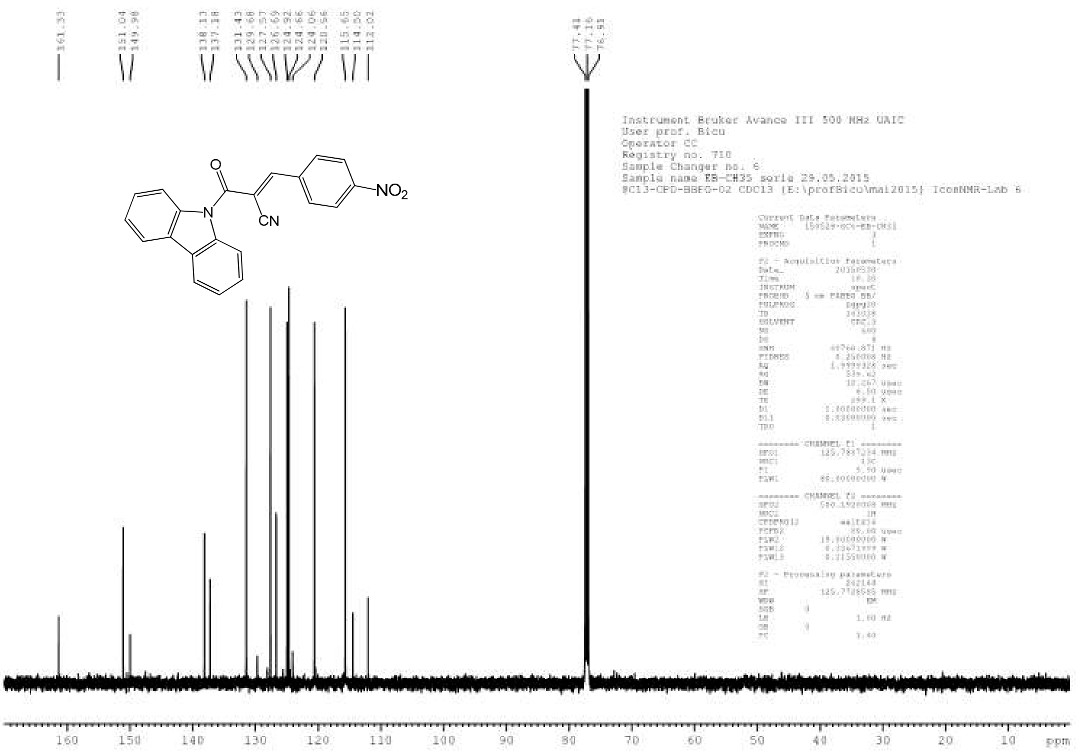

IR-3e

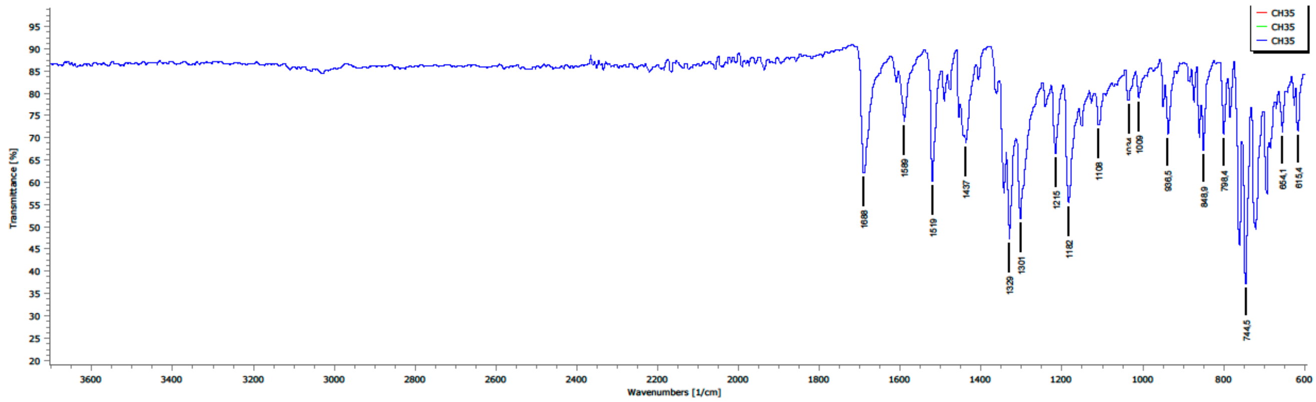

^{1}H NMR (500 MHz, $CDCl_3$)-**3f**

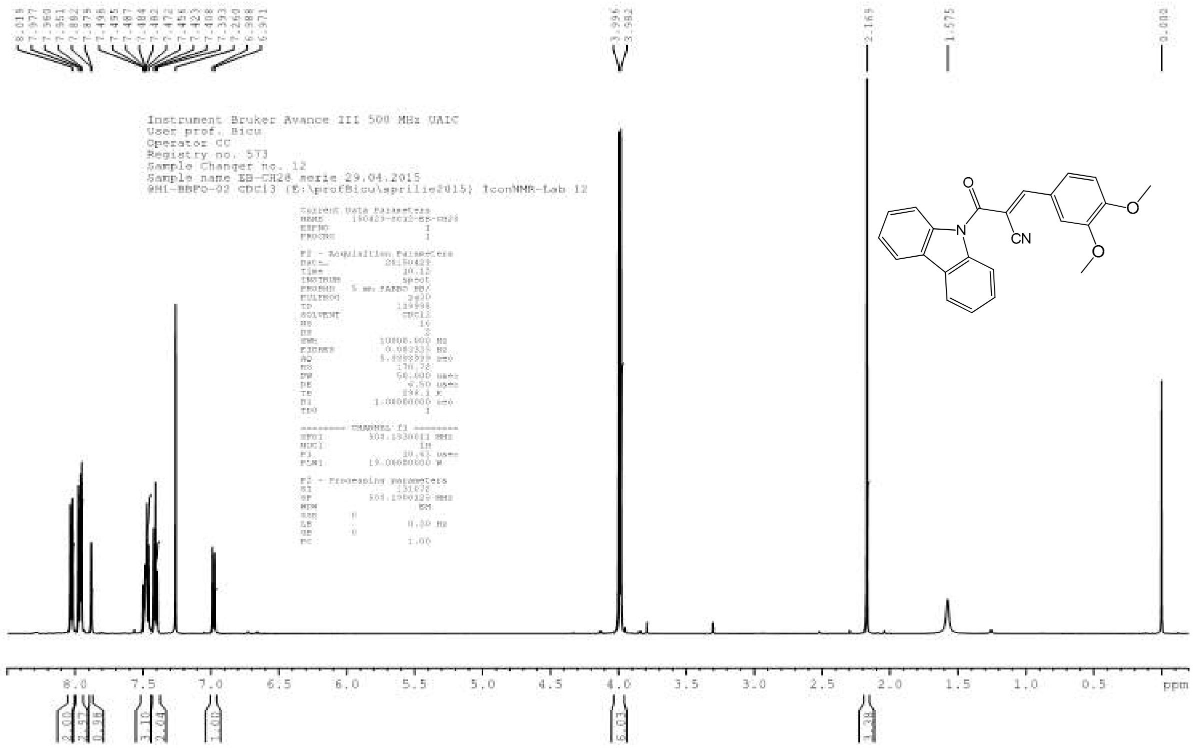

^{13}C NMR (125 MHz, $CDCl_3$)-**3f**

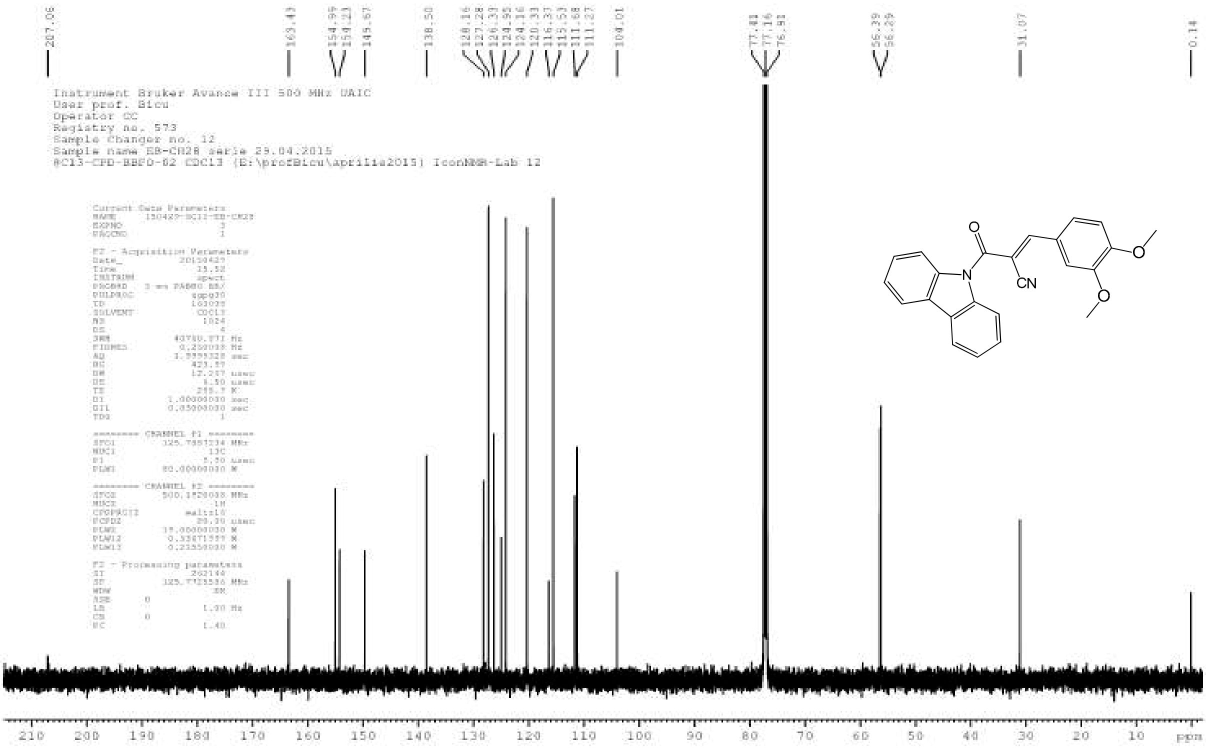

IR-3f

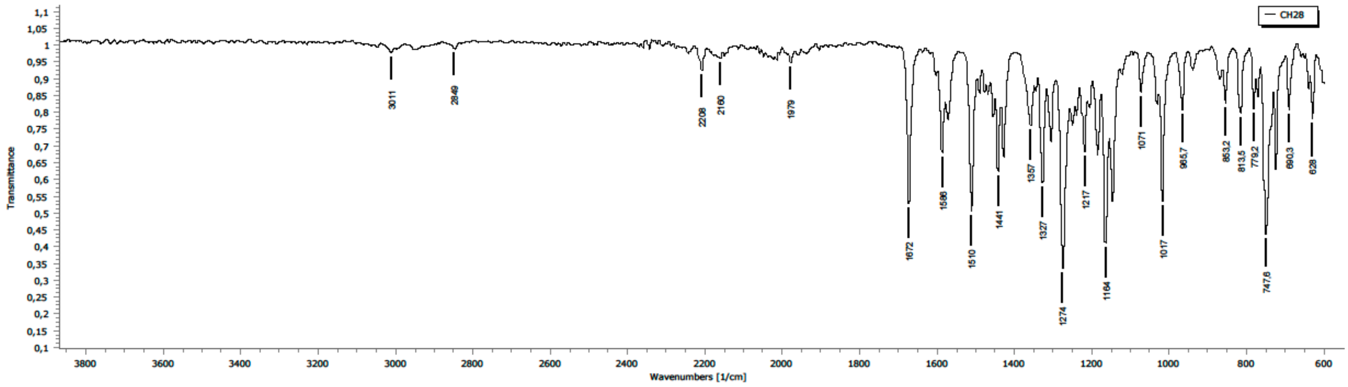

^{1}H NMR (500 MHz, $CDCl_3$)-**3g**

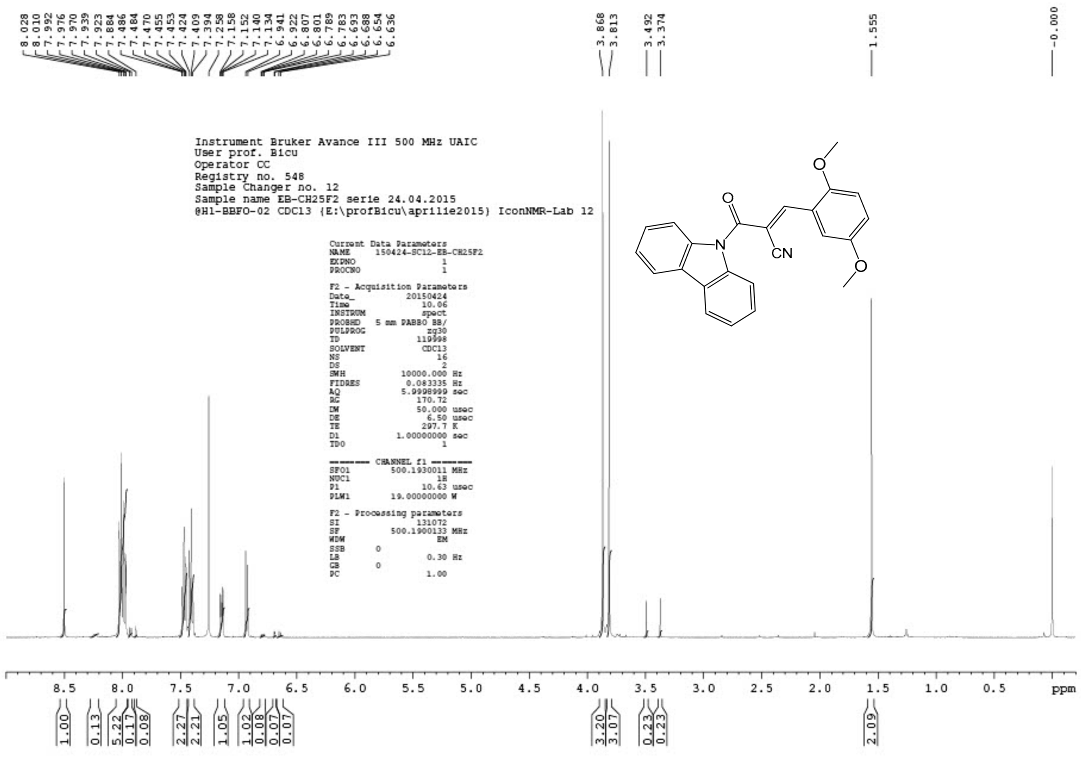

^13^C NMR (125 MHz, $CDCl_3$)**-3g**

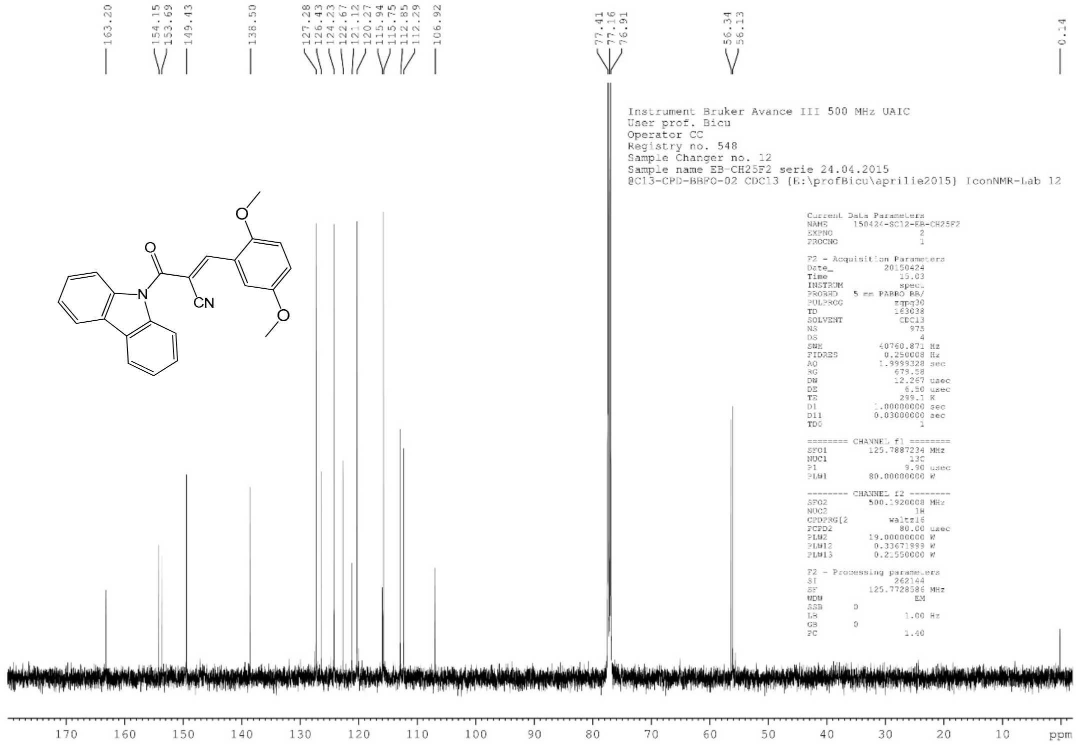

IR-3g

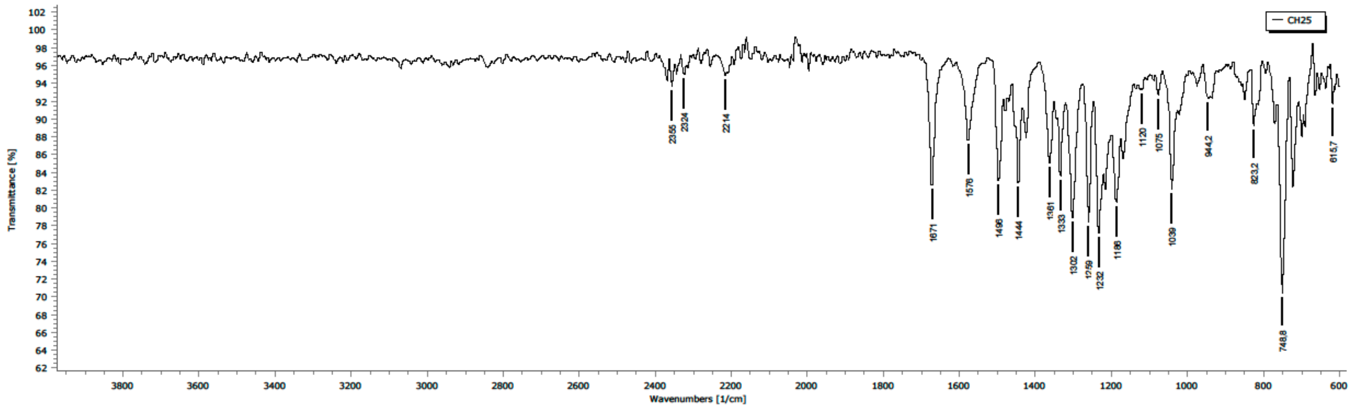

^{1}H NMR (500 MHz, $CDCl_3$)-**3h**

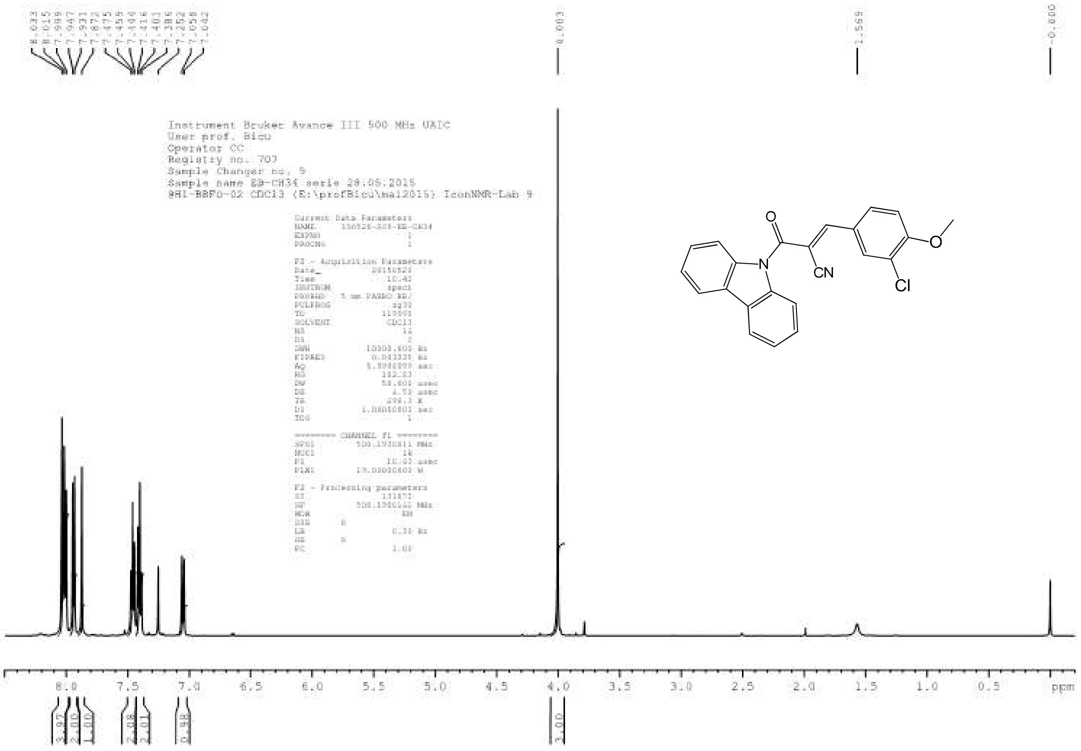

^{13}C NMR (125 MHz, $CDCl_3$)-**3h**

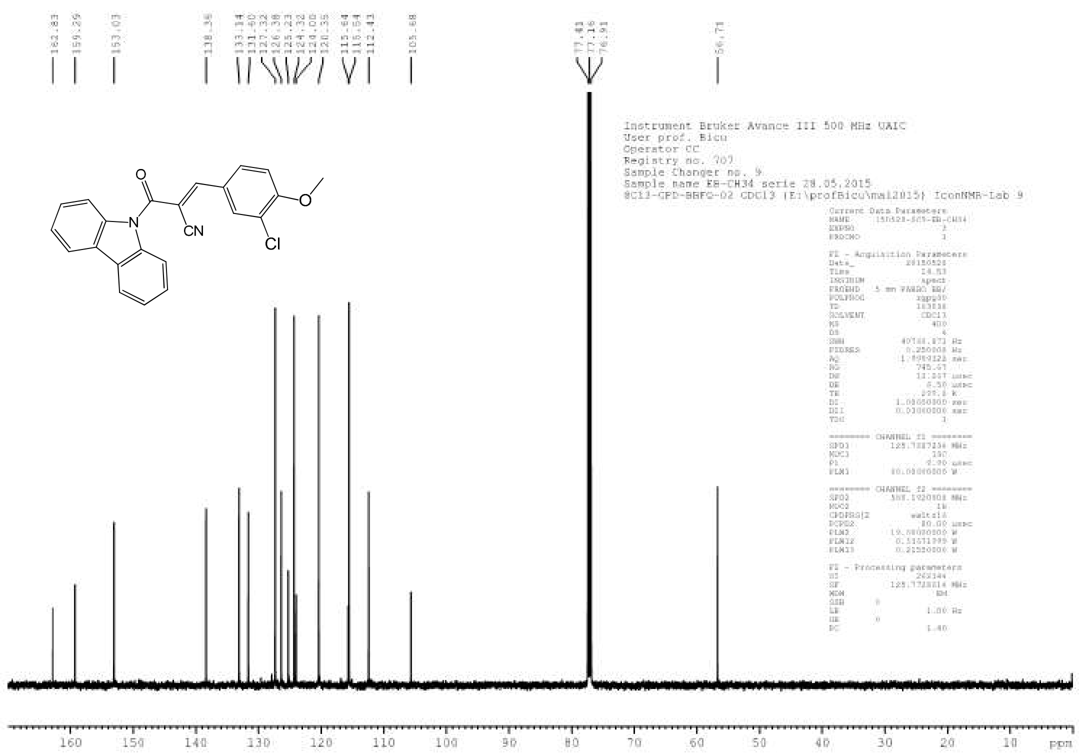
Instrument Bruker Avance III 500 MHz UAIC
User prof. Bicu
Operator CC
Registry no. 707
Sample Changer no. 9
Sample name EB-CH34 serie 28.05.2015
ppm

IR-3h

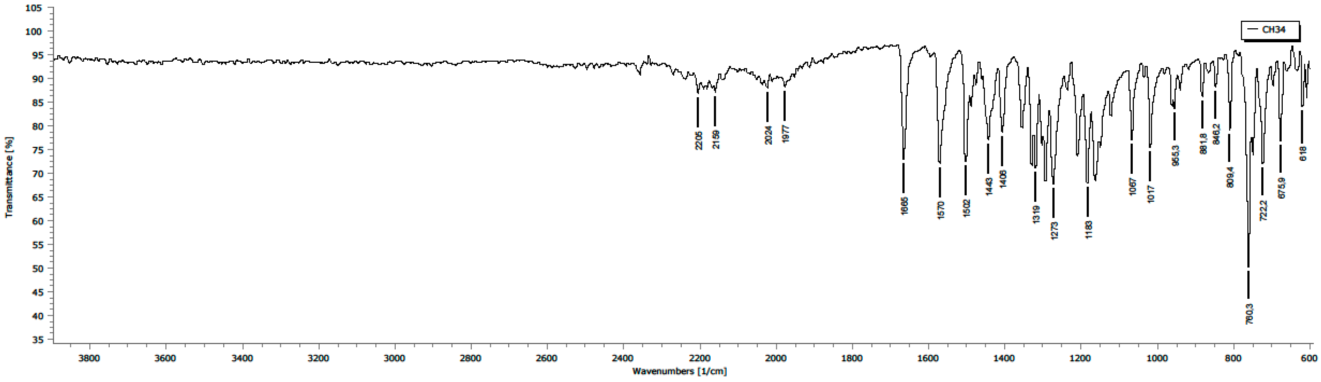
CH34
Transmittance [%]
Wavenumbers [1/cm]

^{1}H NMR (500 MHz, $CDCl_3$)-**3i**

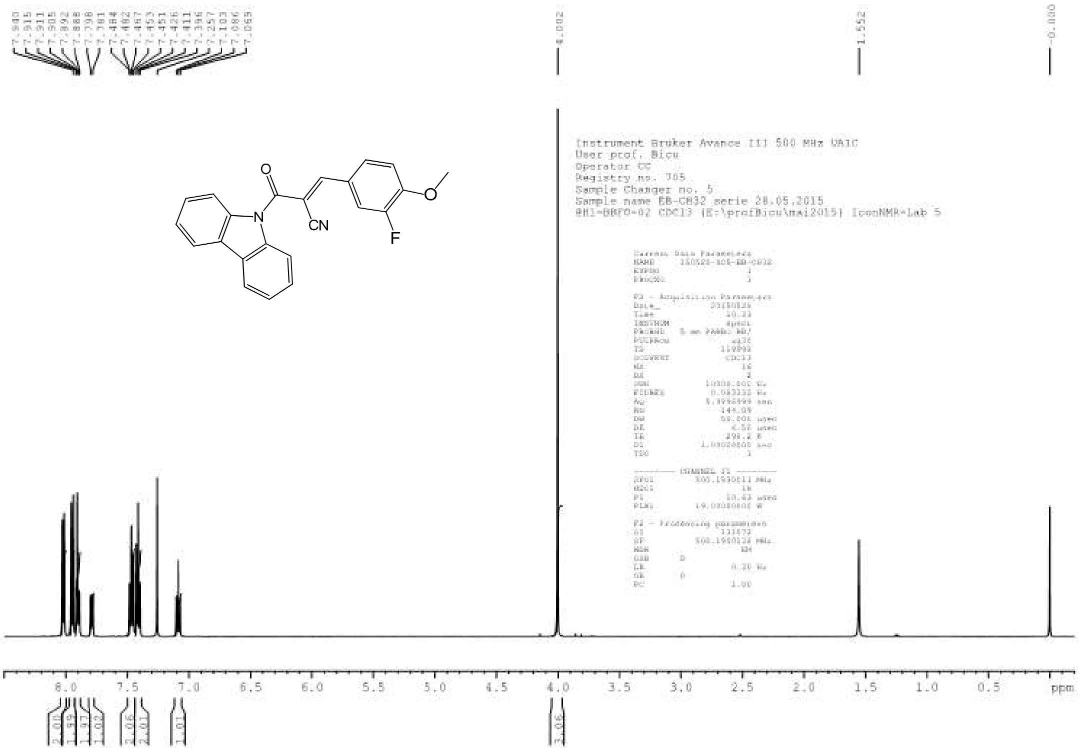

^{13}C NMR (125 MHz, $CDCl_3$)-**3i**

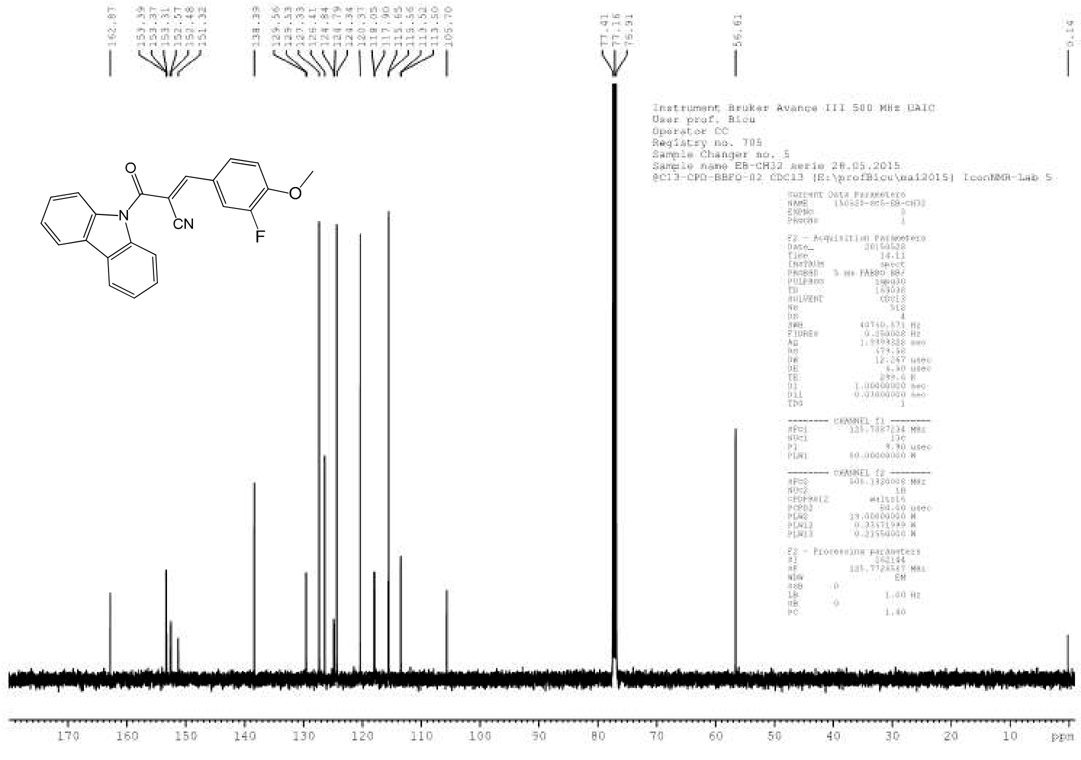

IR-3i

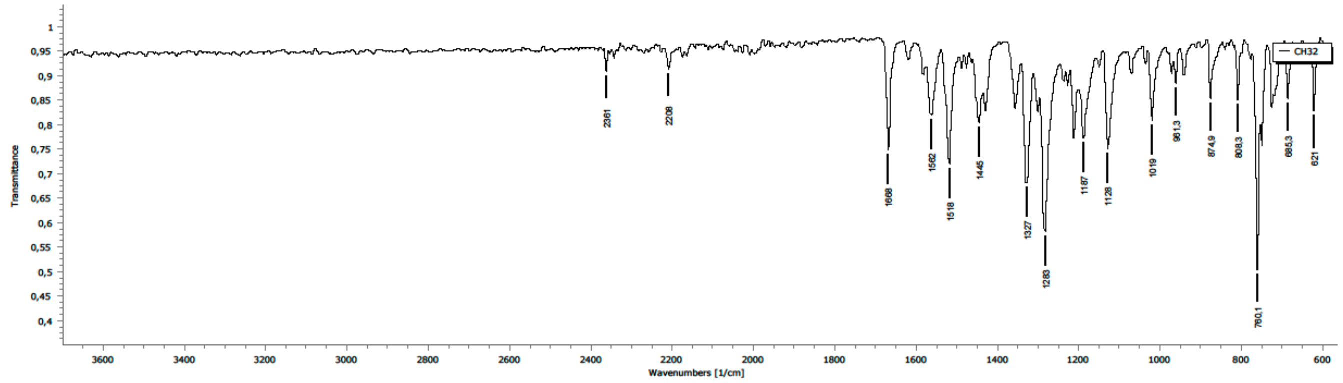

^{1}H NMR (500 MHz, $CDCl_3$)-**3j**

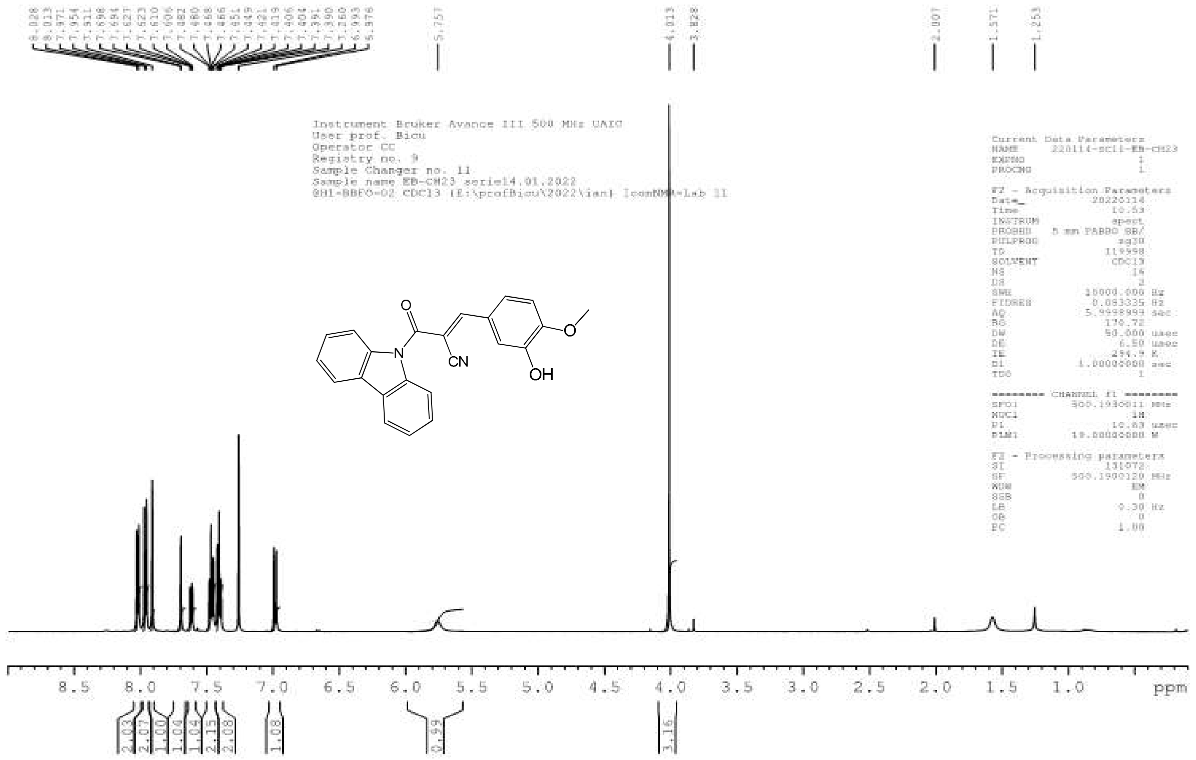

^{13}C NMR (125 MHz, $CDCl_3$)-**3j**

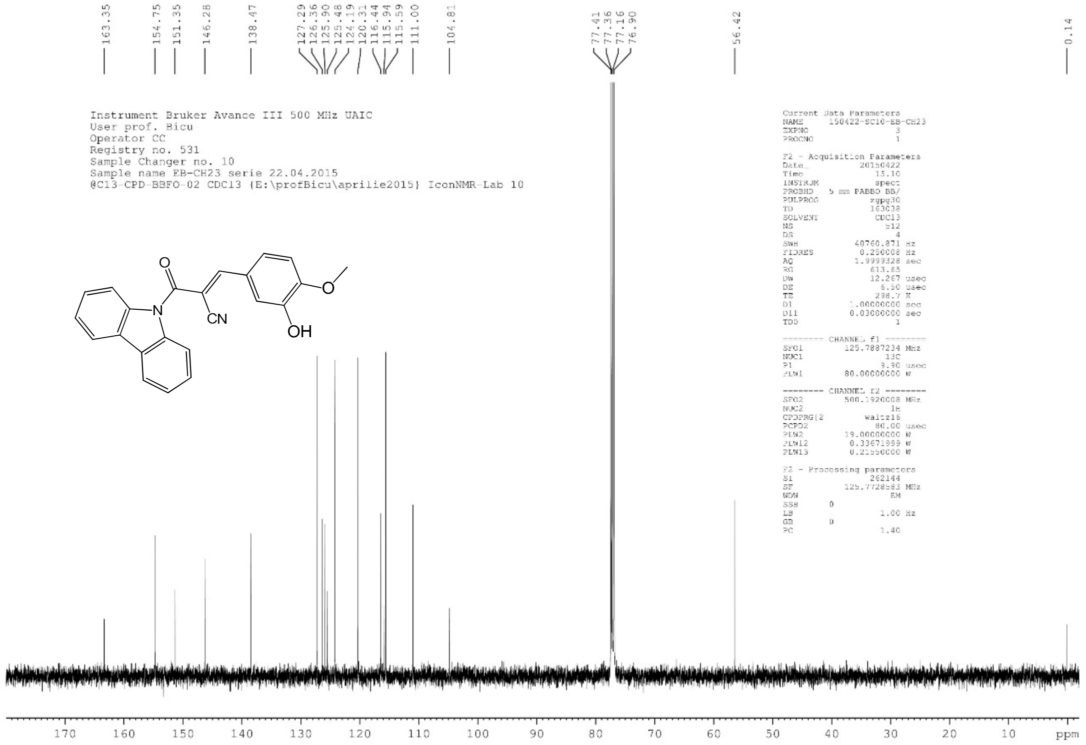

IR-3j

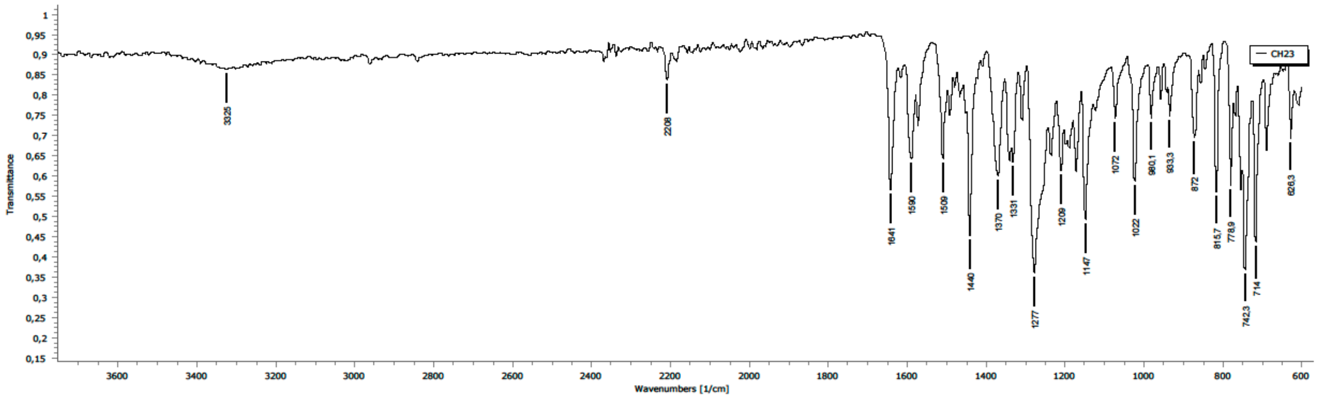

^{1}**H NMR** (500 MHz, $CDCl_3$)-**3k**

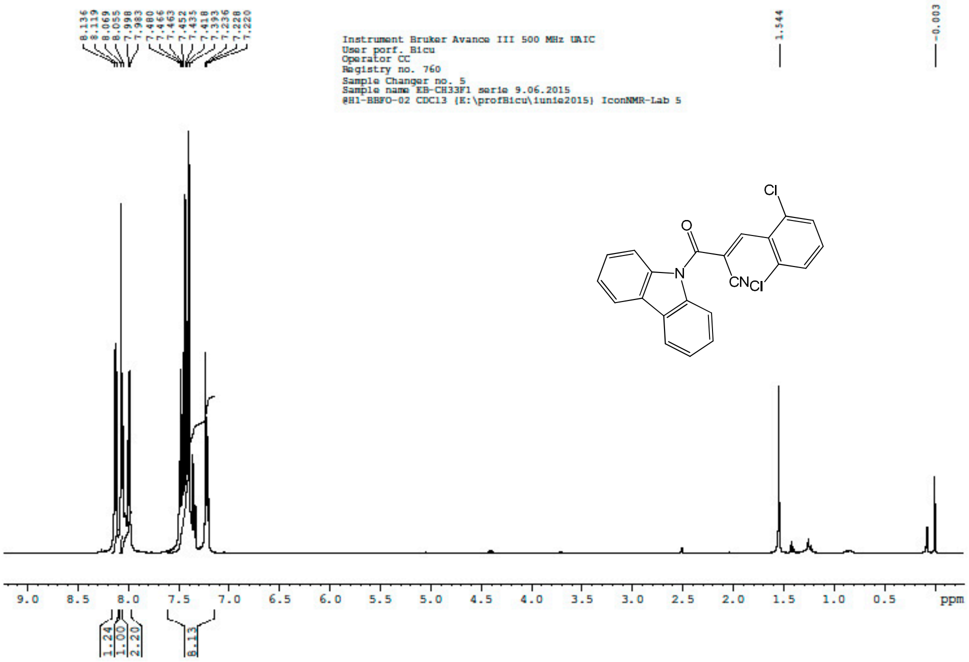

^{13}**C NMR** (125 MHz, $CDCl_3$)-**3k**

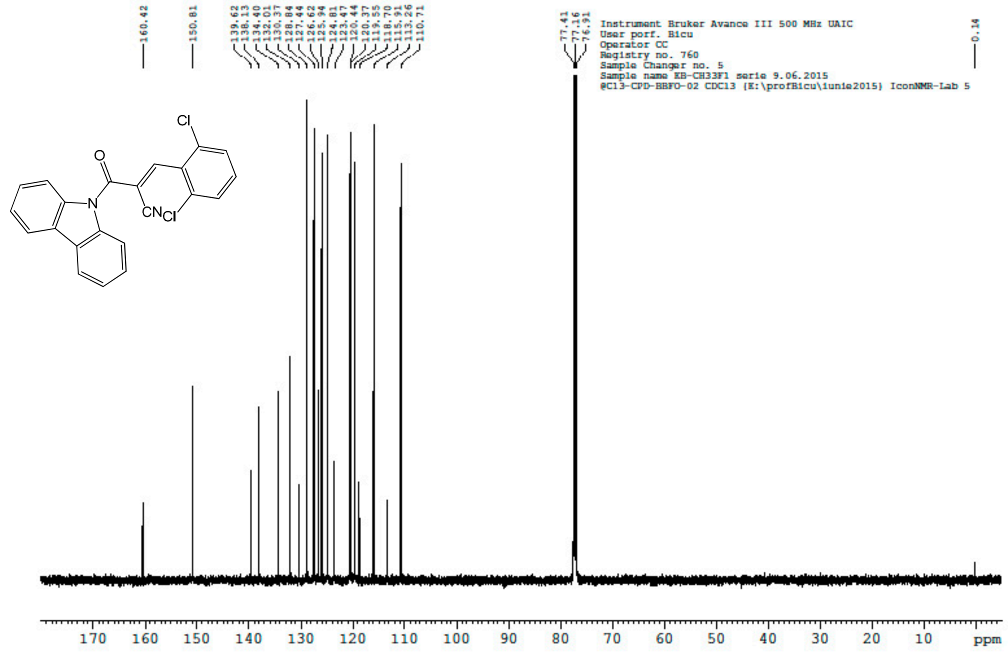

^{13}**C-DEPT NMR** (125 MHz, $CDCl_3$)-**3k**

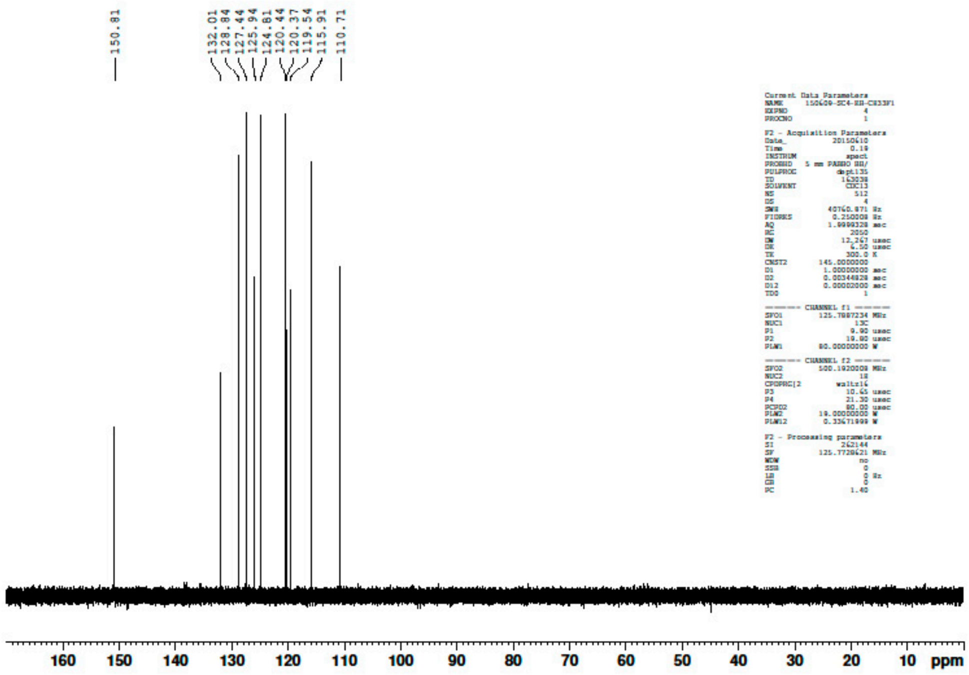

HMQC NMR (500 MHz, $CDCl_3$)-**3k**

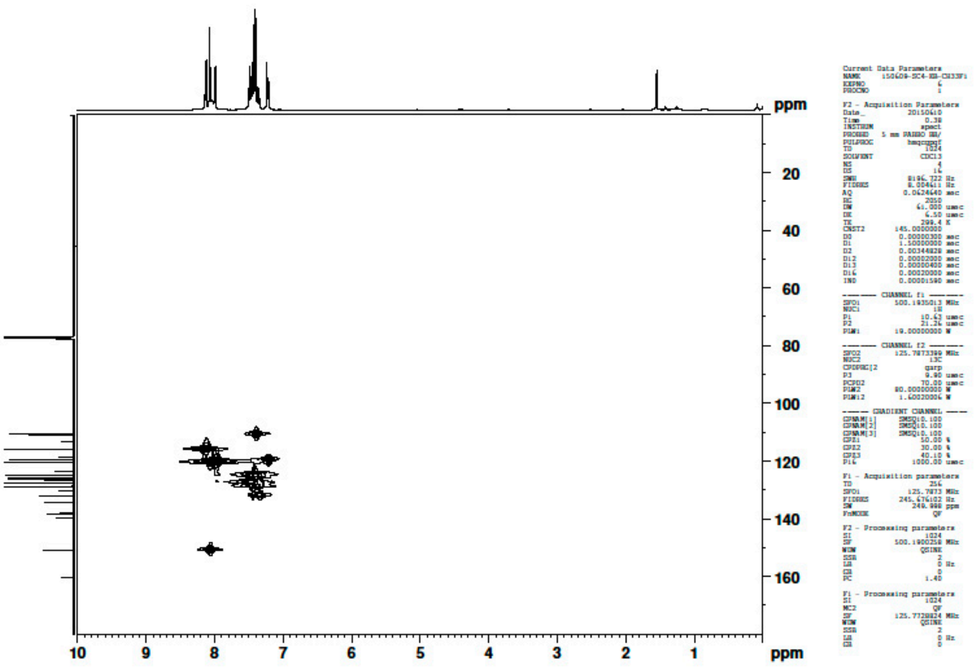

IR-3k

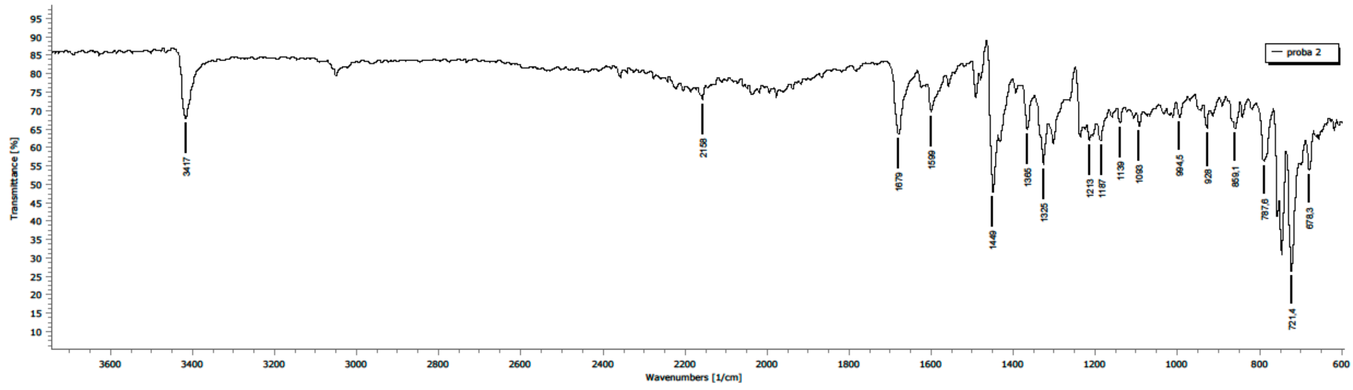

^{1}H NMR (500 MHz, DMSO-d_6)-**3l**

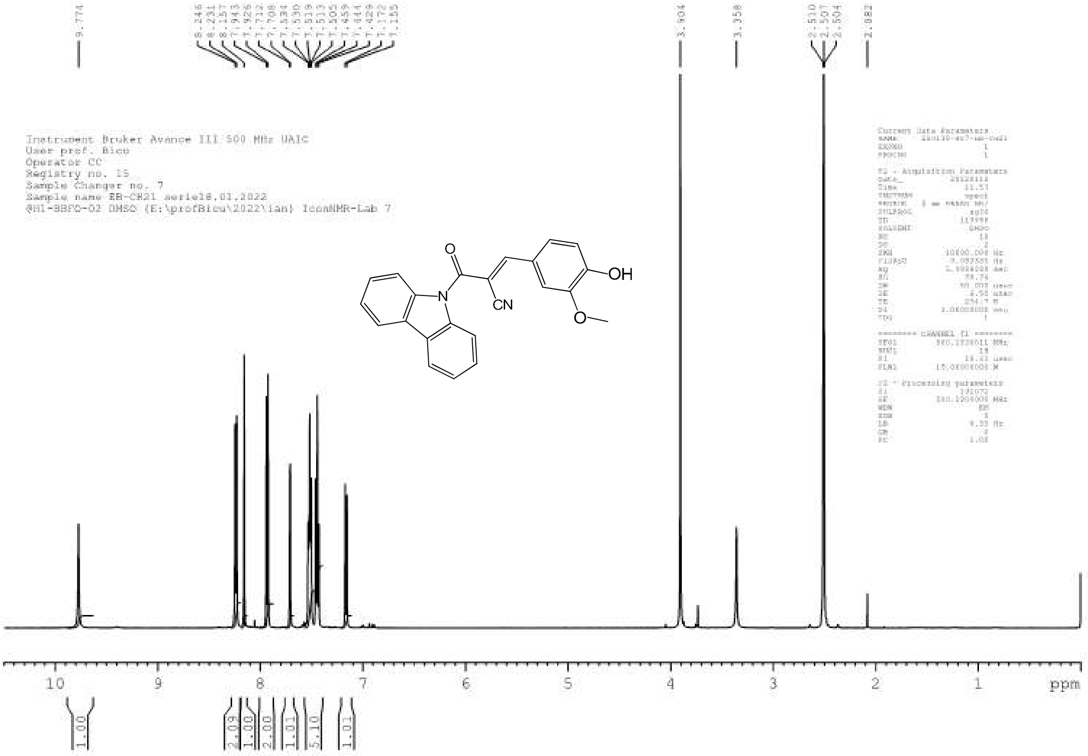

^{13}C NMR (125 MHz, DMSO-*d*$_6$)- **3l**

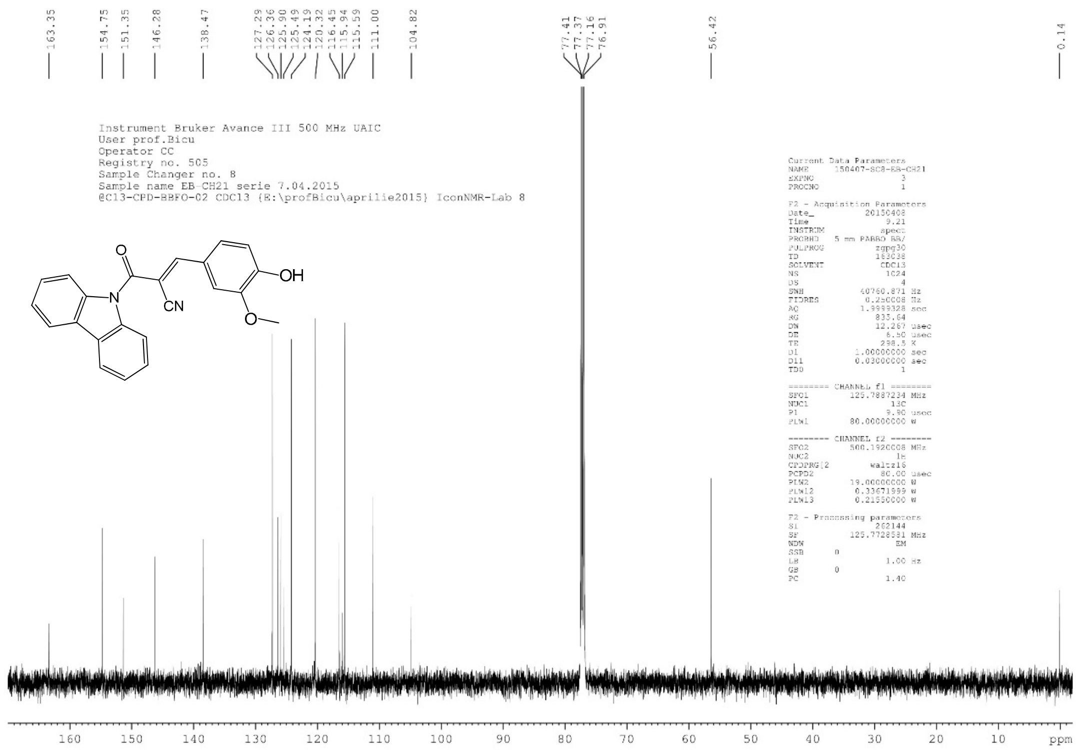

IR-3l

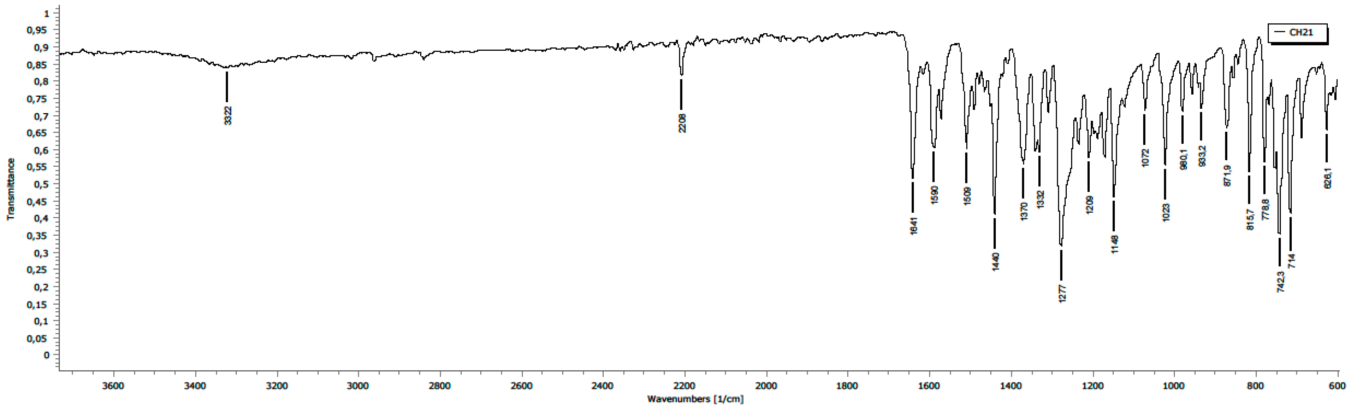

^{1}**H NMR** (500 MHz, $CDCl_3$)-**3m**

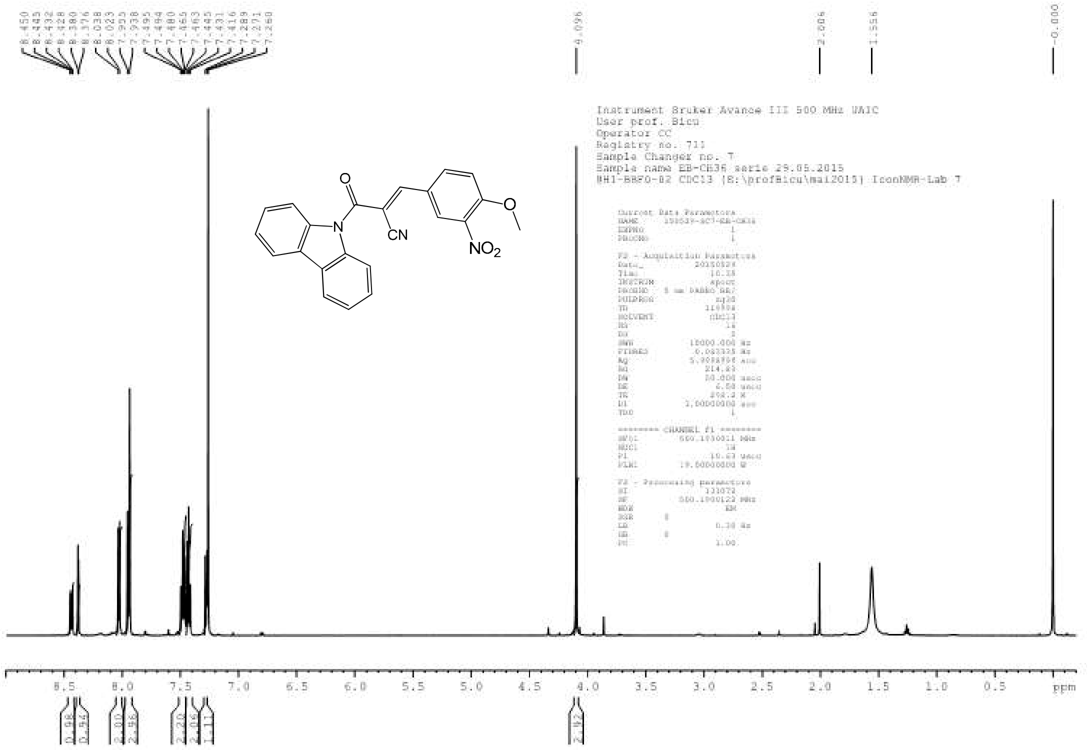

^{13}**C NMR** (125 MHz, $CDCl_3$)- **3m**

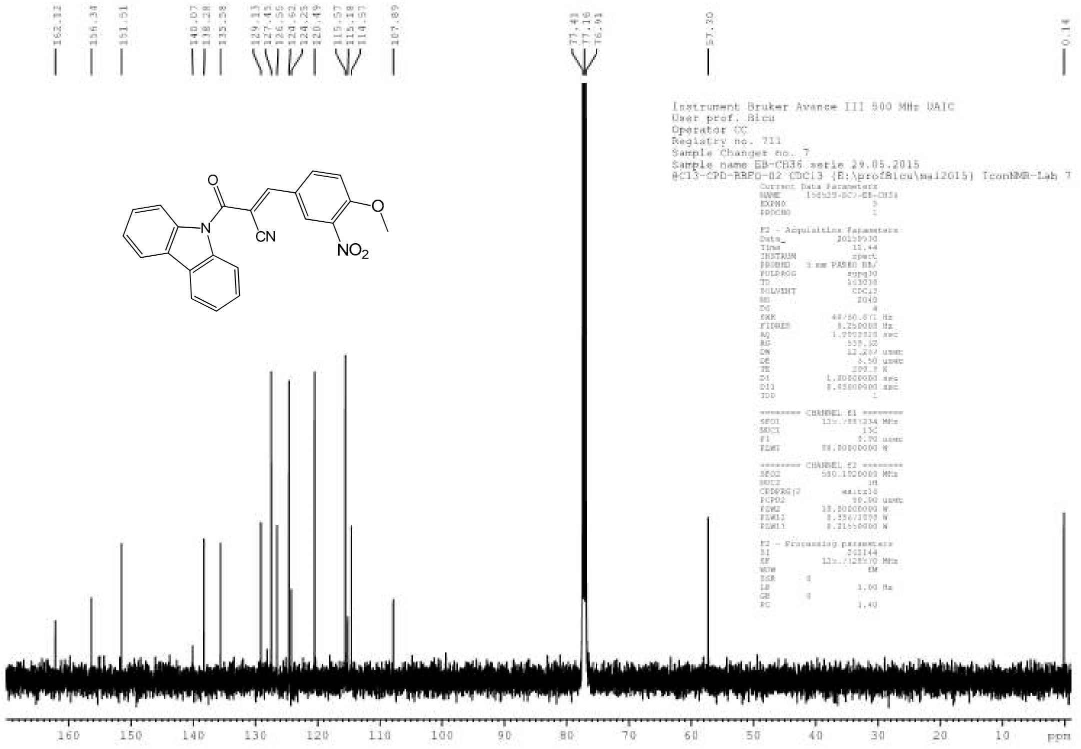

IR-3m

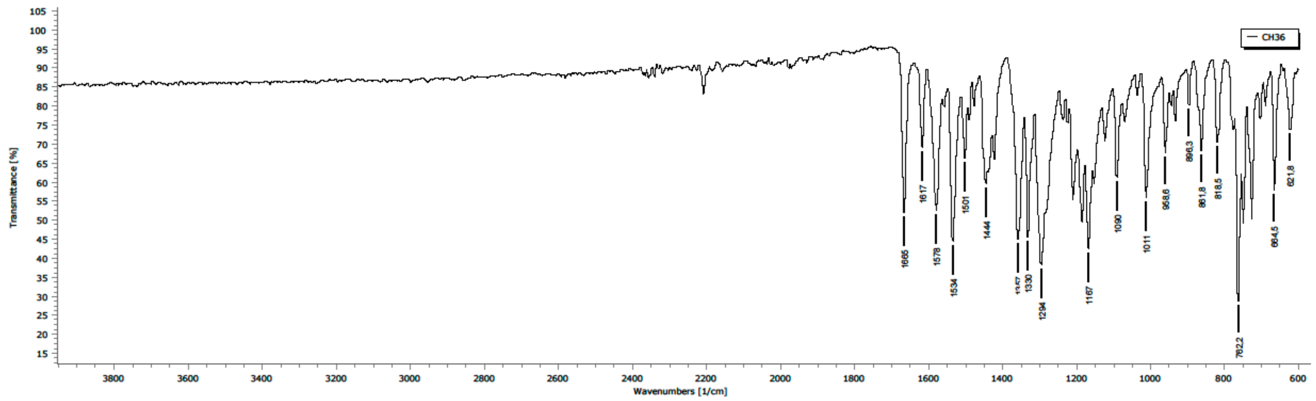

^{1}H NMR (500 MHz, $CDCl_3$)-**3n**

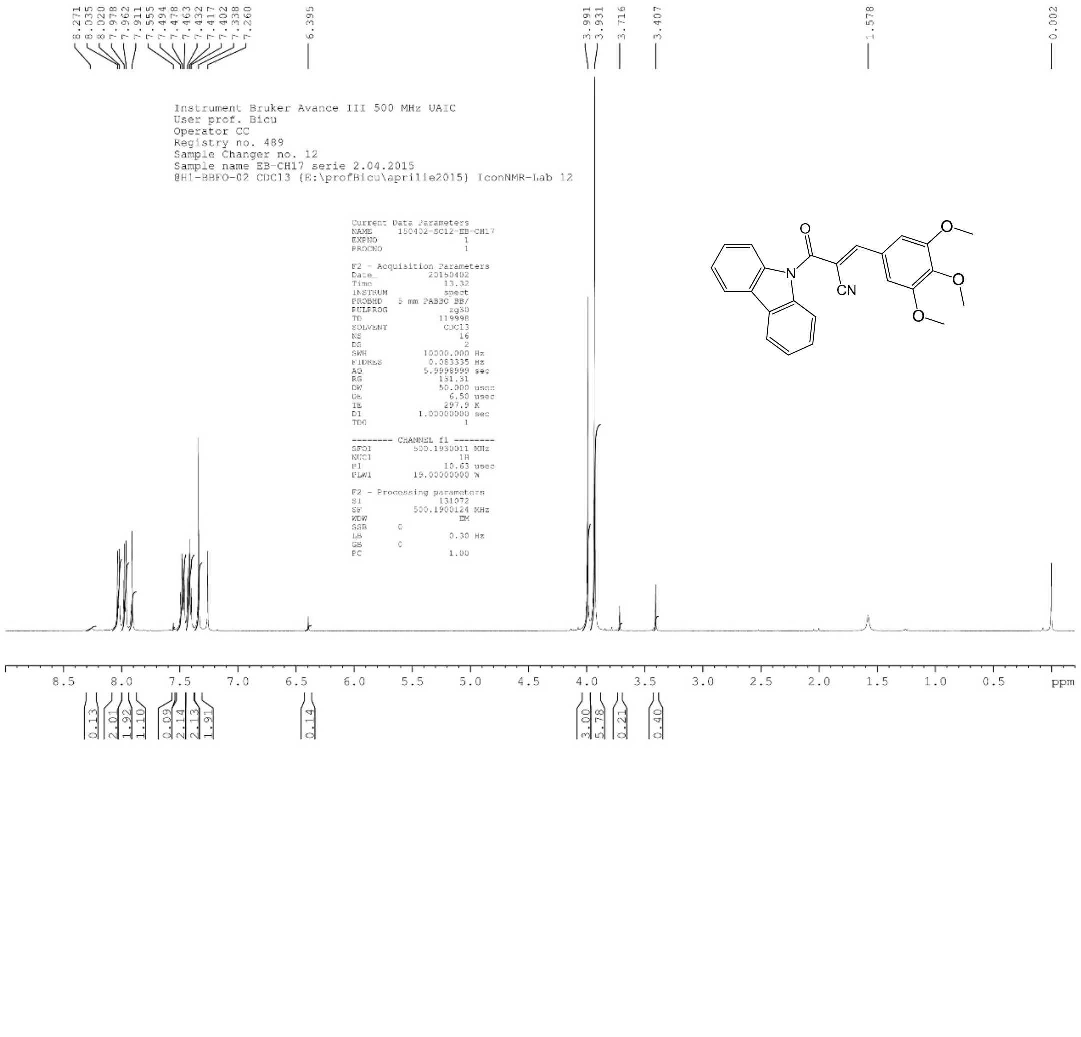

^{13}C NMR (125 MHz, $CDCl_3$)- **3n**

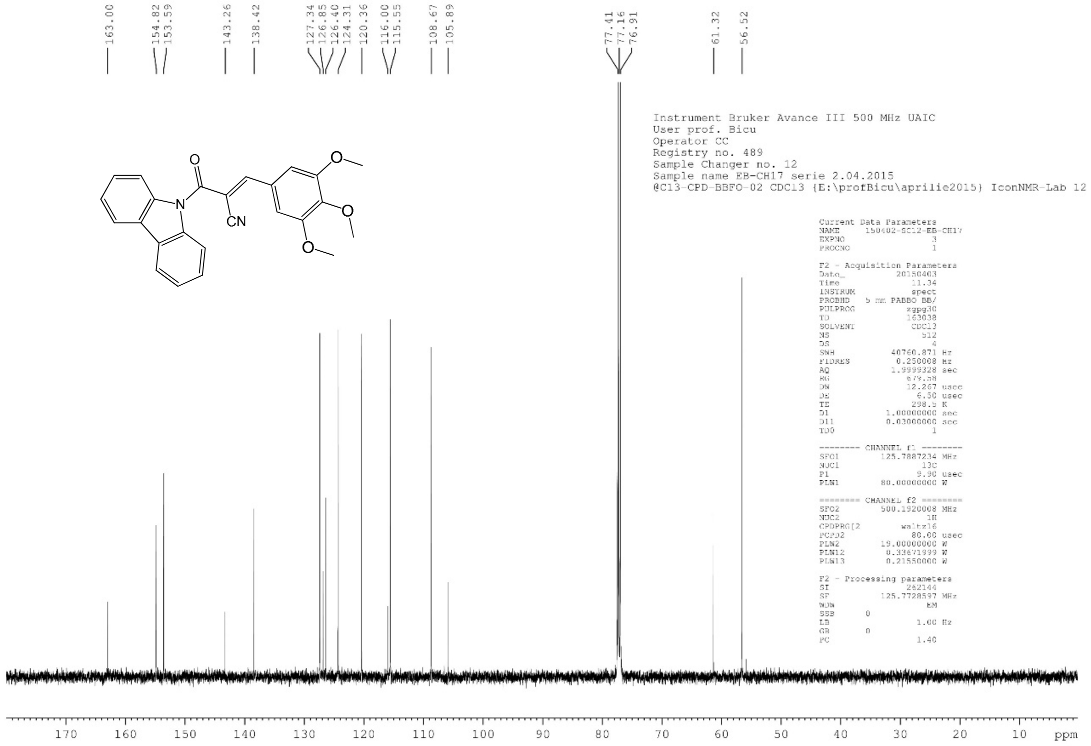

IR-3n

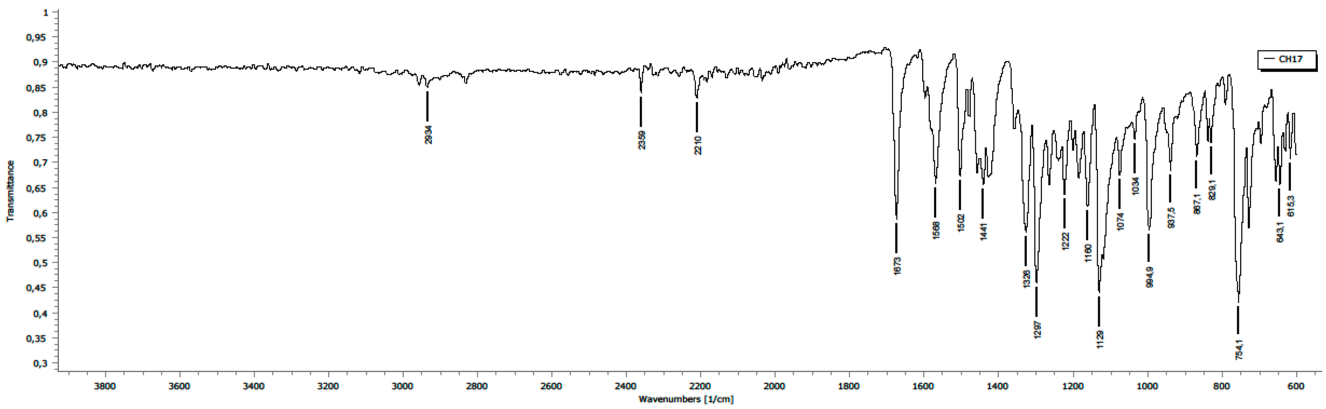

^{1}H NMR (500 MHz, $CDCl_3$)-**3o**

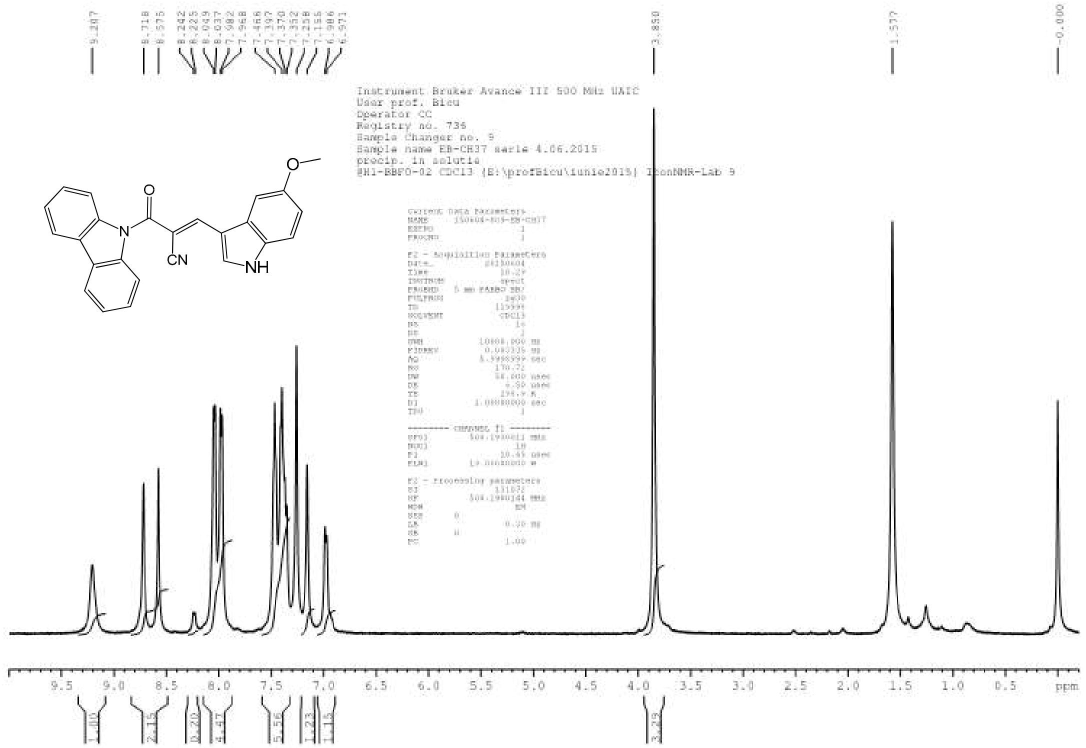

^{13}C NMR (125 MHz, $CDCl_3$)- **3o**

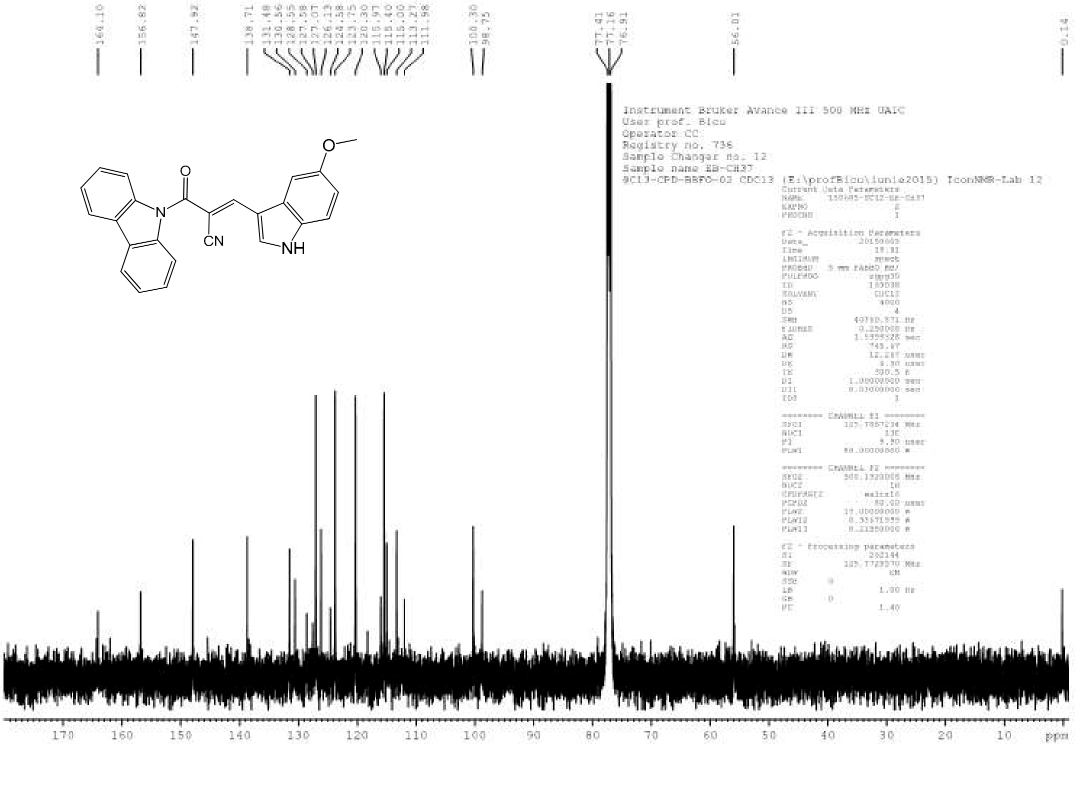

IR-3o

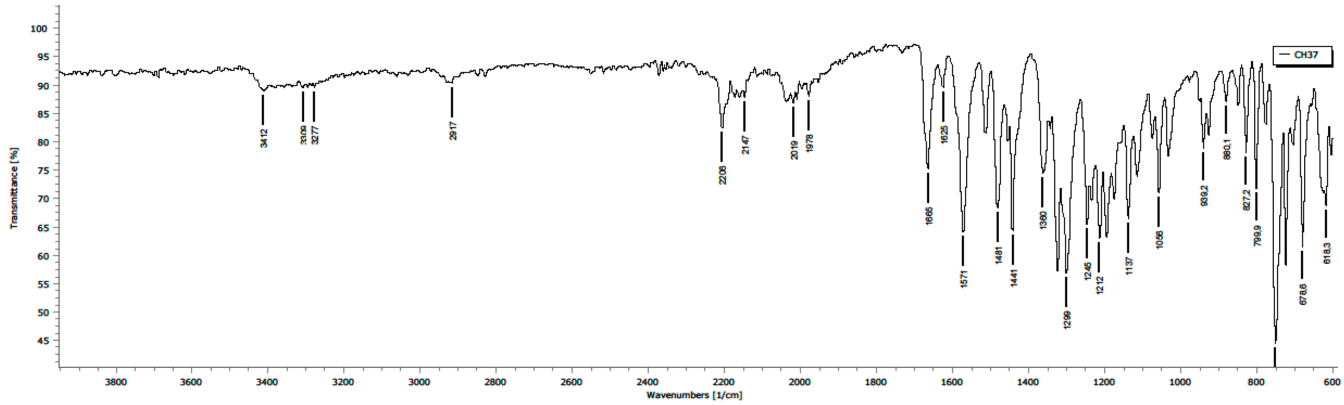

^{1}H NMR (500 MHz, $CDCl_3$)-**3p**

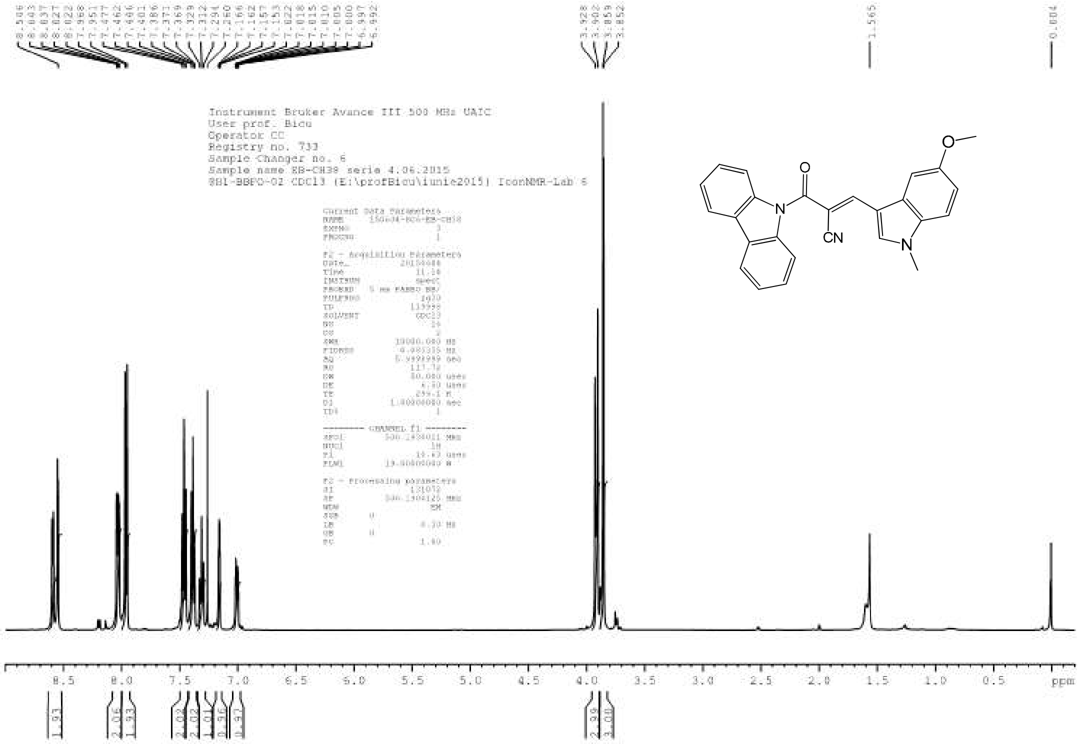

^{13}**C NMR** (125 MHz, $CDCl_3$)-**3p**

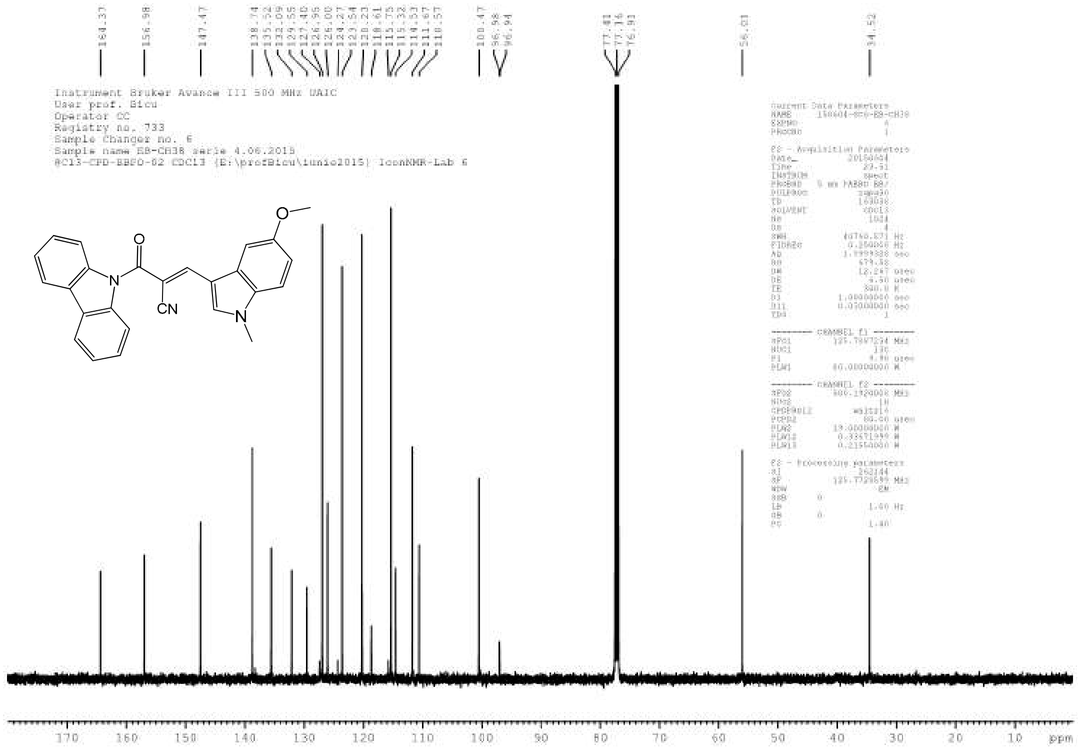

IR-3p

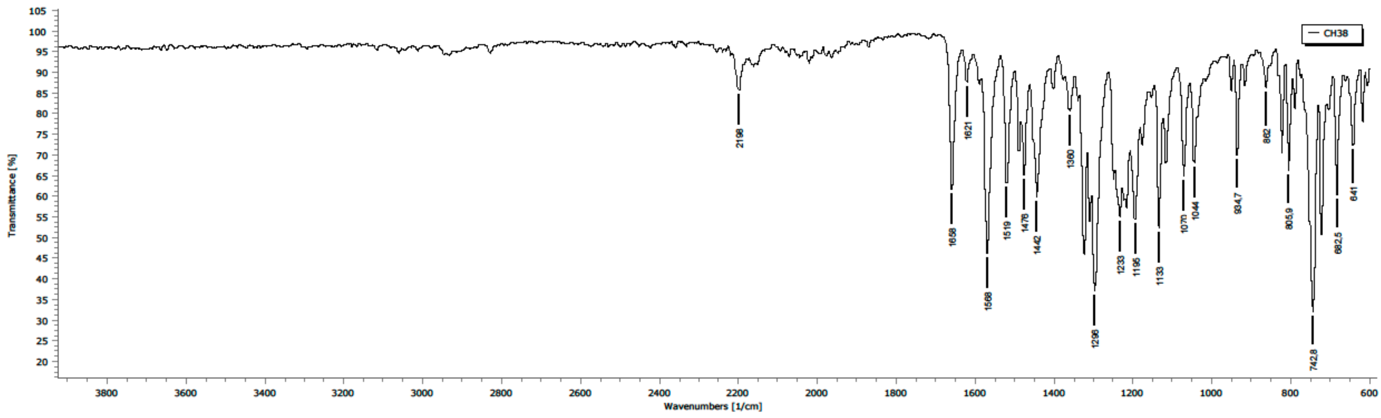

^{1}H NMR (500 MHz, $CDCl_3$)-**4b**

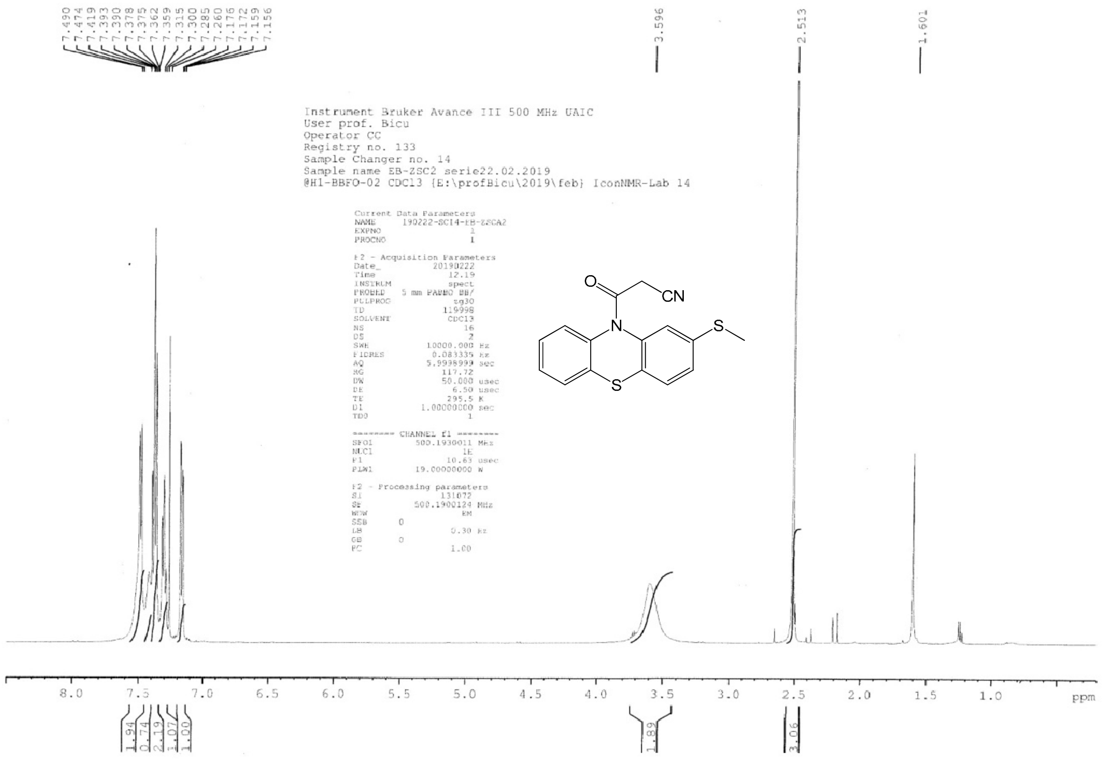

^{13}C NMR (125 MHz, $CDCl_3$)-**4b**

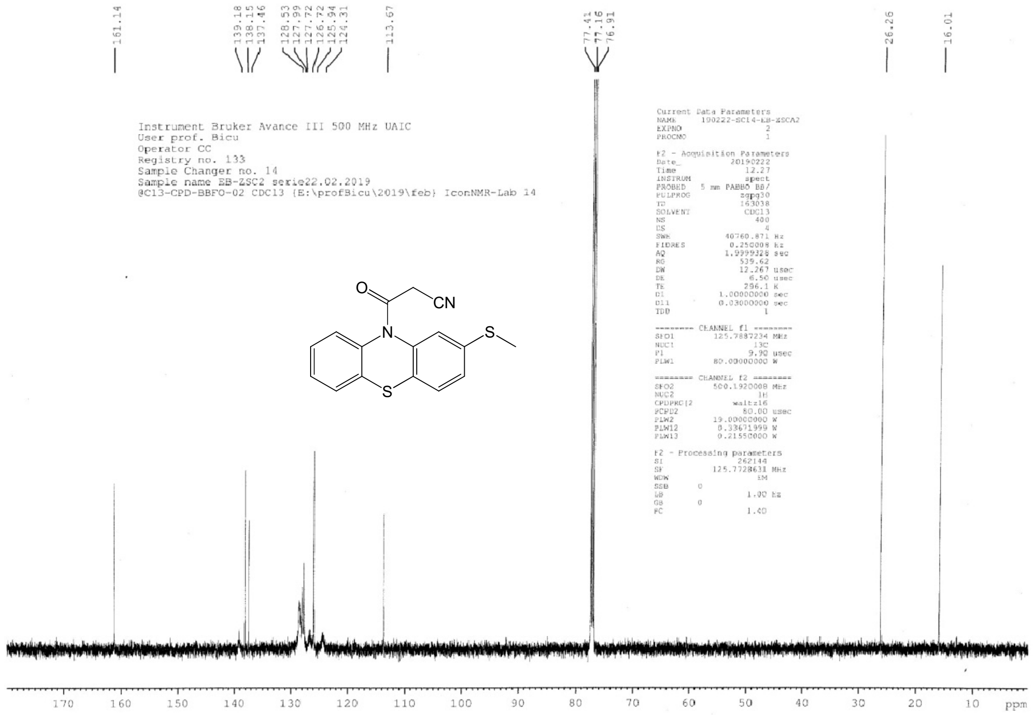

IR-4b

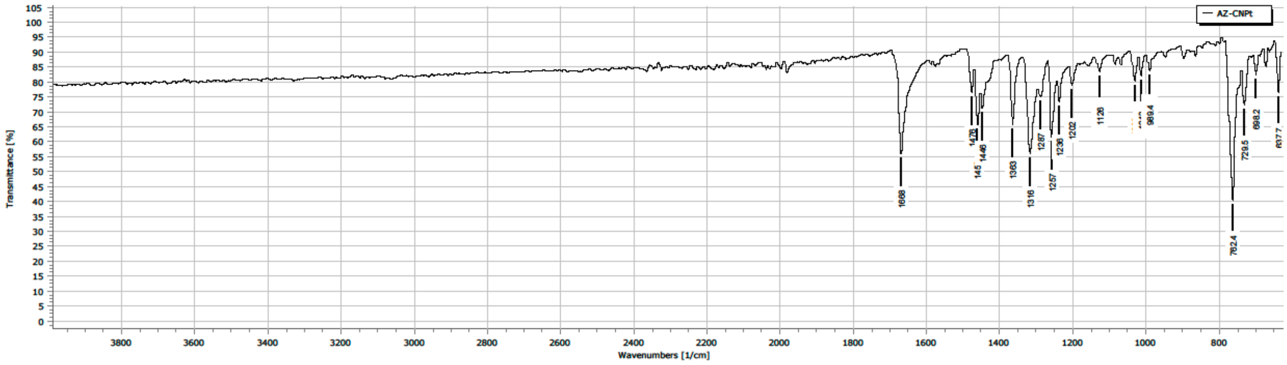

AZ-CNPt
Transmittance [%]
Wavenumbers [1/cm]
105
100
95
90
85
80
75
70
65
60
55
50
45
40
35
30
25
20
15
10
5
0
3800
3600
3400
3200
3000
2800
2600
2400
2200
2000
1800
1600
1400
1200
1000
800
1668
1476
145
1446
1363
1316
1287
1257
1236
1202
1126
989.4
762.4
729.5
698.2
637.7

One-dose full graphs obtained on NCI-60 cancer cell lines panel

Compound 2k

Developmental Therapeutics Program One Dose Mean Graph	NSC: D-827147 / 1	Conc: 1.00E-5 Molar	Test Date: Nov 30, 2020
	Experiment ID: 2011OS67		Report Date: Dec 16, 2020

Panel/Cell Line	Growth Percent
**Leukemia**	
CCRF-CEM	18.99
HL-60(TB)	12.80
K-562	16.38
MOLT-4	30.46
RPMI-8226	21.32
SR	34.35
**Non-Small Cell Lung Cancer**	
A549/ATCC	47.28
EKVX	34.77
HOP-62	59.93
HOP-92	44.06
NCI-H226	50.43
NCI-H322M	86.10
NCI-H460	45.51
NCI-H522	5.48
**Colon Cancer**	
COLO 205	40.79
HCT-116	24.34
HCT-15	27.26
HT29	12.19
KM12	24.49
SW-620	34.51
**CNS Cancer**	
SF-268	55.83
SF-295	14.96
SF-539	7.54
SNB-19	41.00
SNB-75	8.61
U251	42.09
**Melanoma**	
MALME-3M	55.69
M14	29.49
MDA-MB-435	11.71
SK-MEL-2	35.60
SK-MEL-28	58.52
UACC-257	56.96
UACC-62	41.80
**Ovarian Cancer**	
IGROV1	40.17
OVCAR-3	37.49
OVCAR-4	59.49
OVCAR-5	63.07
OVCAR-8	52.43
**Renal Cancer**	
786-0	49.97
A498	10.83
ACHN	39.92
CAKI-1	38.49
RXF 393	38.76
SN12C	33.94
TK-10	71.28
UO-31	50.45
**Prostate Cancer**	
PC-3	28.83
DU-145	51.28
**Breast Cancer**	
MCF7	23.07
MDA-MB-231/ATCC	53.23
HS 578T	37.77
BT-549	55.83
T-47D	29.86
MDA-MB-468	12.00
Mean	37.21
Delta	31.73
Range	80.62

Mean Growth Percent - Growth Percent

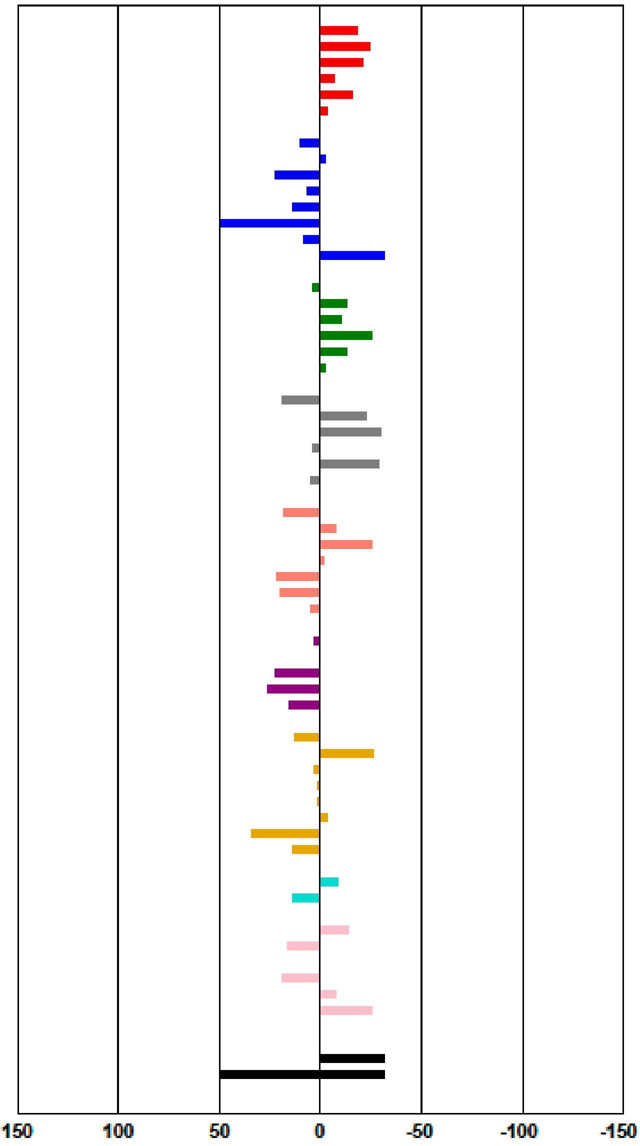

Compound 2l

Developmental Therapeutics Program One Dose Mean Graph	NSC: D-826472 / 1	Conc: 1.00E-5 Molar	Test Date: Oct 26, 2020
	Experiment ID: 2010OS59		Report Date: Dec 02, 2020

Panel/Cell Line	Growth Percent
**Leukemia**	
CCRF-CEM	51.35
HL-60(TB)	80.79
K-562	56.82
MOLT-4	60.08
SR	101.63
**Non-Small Cell Lung Cancer**	
A549/ATCC	66.11
EKVX	71.42
HOP-62	70.62
HOP-92	109.28
NCI-H226	64.54
NCI-H23	75.15
NCI-H322M	95.35
NCI-H460	95.46
NCI-H522	60.62
**Colon Cancer**	
COLO 205	86.61
HCC-2998	96.25
HCT-116	59.81
HCT-15	55.49
HT29	57.67
KM12	50.69
SW-620	85.43
**CNS Cancer**	
SF-268	70.97
SF-295	79.40
SF-539	87.65
SNB-19	91.14
U251	73.15
**Melanoma**	
MALME-3M	84.81
M14	64.12
MDA-MB-435	80.36
SK-MEL-2	94.37
SK-MEL-28	96.92
SK-MEL-5	69.56
UACC-257	92.25
UACC-62	89.12
**Ovarian Cancer**	
IGROV1	86.39
OVCAR-3	83.95
OVCAR-4	102.58
OVCAR-5	81.30
OVCAR-8	61.92
NCI/ADR-RES	71.20
SK-OV-3	93.59
**Renal Cancer**	
786-0	86.54
ACHN	76.55
CAKI-1	51.01
RXF 393	73.39
SN12C	80.34
TK-10	84.49
UO-31	60.69
**Prostate Cancer**	
PC-3	39.92
DU-145	85.09
**Breast Cancer**	
MCF7	75.30
MDA-MB-231/ATCC	80.04
HS 578T	80.12
BT-549	98.43
T-47D	42.87
MDA-MB-468	68.29
Mean	76.59
Delta	36.67
Range	69.36

Mean Growth Percent - Growth Percent

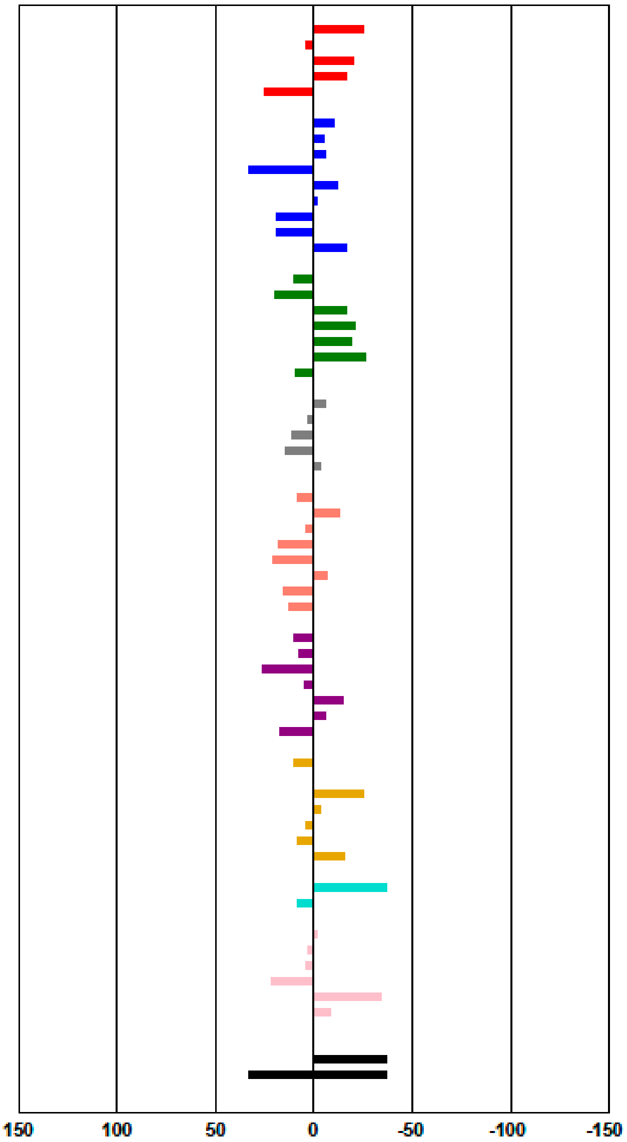

Compound 2o

Developmental Therapeutics Program One Dose Mean Graph	NSC: D-826274 / 1	Conc: 1.00E-5 Molar	Test Date: Oct 19, 2020
	Experiment ID: 2010OS56		Report Date: Nov 12, 2020

Panel/Cell Line	Growth Percent
**Leukemia**	
CCRF-CEM	53.54
HL-60(TB)	54.06
K-562	58.15
MOLT-4	58.85
RPMI-8226	64.14
SR	86.86
**Non-Small Cell Lung Cancer**	
A549/ATCC	77.02
EKVX	75.36
HOP-62	73.85
NCI-H226	85.58
NCI-H23	72.43
NCI-H322M	87.82
NCI-H460	79.54
NCI-H522	73.44
**Colon Cancer**	
COLO 205	111.11
HCC-2998	87.66
HCT-116	69.98
HCT-15	57.47
HT29	86.06
KM12	84.77
SW-620	72.74
**CNS Cancer**	
SF-268	78.87
SF-295	88.98
SF-539	94.57
SNB-19	79.80
SNB-75	64.95
U251	75.90
**Melanoma**	
LOX IMVI	63.77
M14	85.24
MDA-MB-435	64.49
SK-MEL-2	93.50
SK-MEL-28	83.87
SK-MEL-5	89.45
UACC-257	68.87
UACC-62	62.40
**Ovarian Cancer**	
IGROV1	80.95
OVCAR-3	66.62
OVCAR-4	73.76
OVCAR-5	94.35
OVCAR-8	82.26
NCI/ADR-RES	68.71
SK-OV-3	99.01
**Renal Cancer**	
786-0	83.46
A498	71.88
ACHN	95.92
CAKI-1	60.42
RXF 393	67.90
SN12C	77.21
TK-10	119.10
UO-31	67.87
**Prostate Cancer**	
PC-3	55.14
DU-145	75.06
**Breast Cancer**	
MCF7	41.74
MDA-MB-231/ATCC	104.82
HS 578T	84.83
BT-549	87.30
T-47D	53.12
MDA-MB-468	40.50
Mean	76.16
Delta	35.66
Range	78.60

Mean Growth Percent - Growth Percent

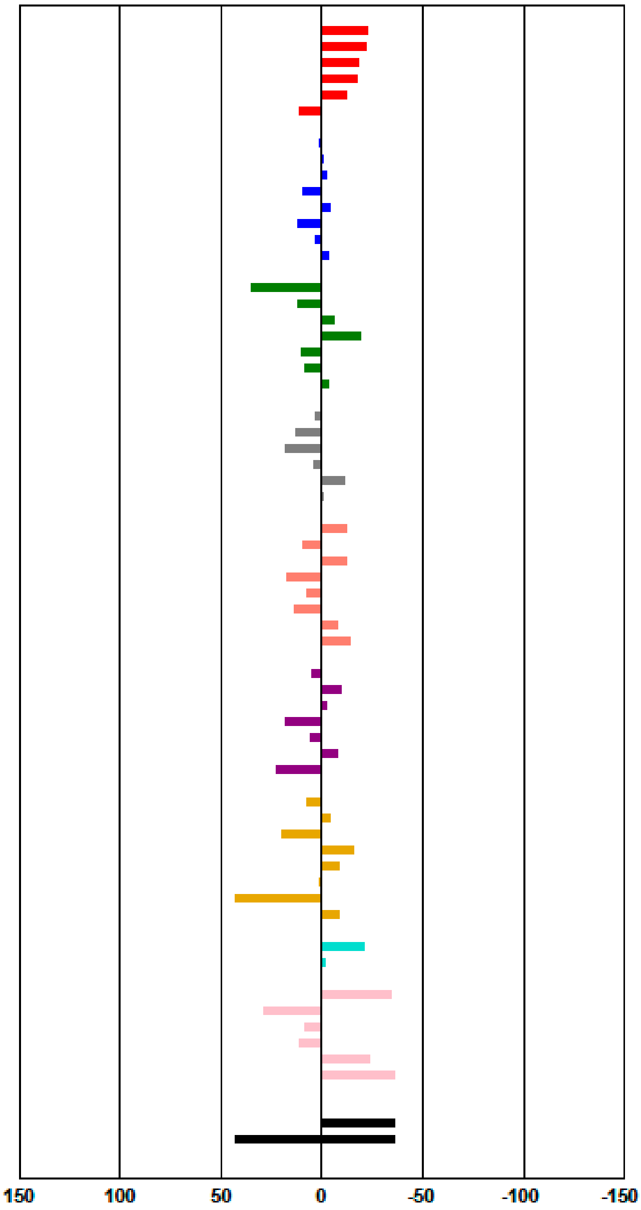

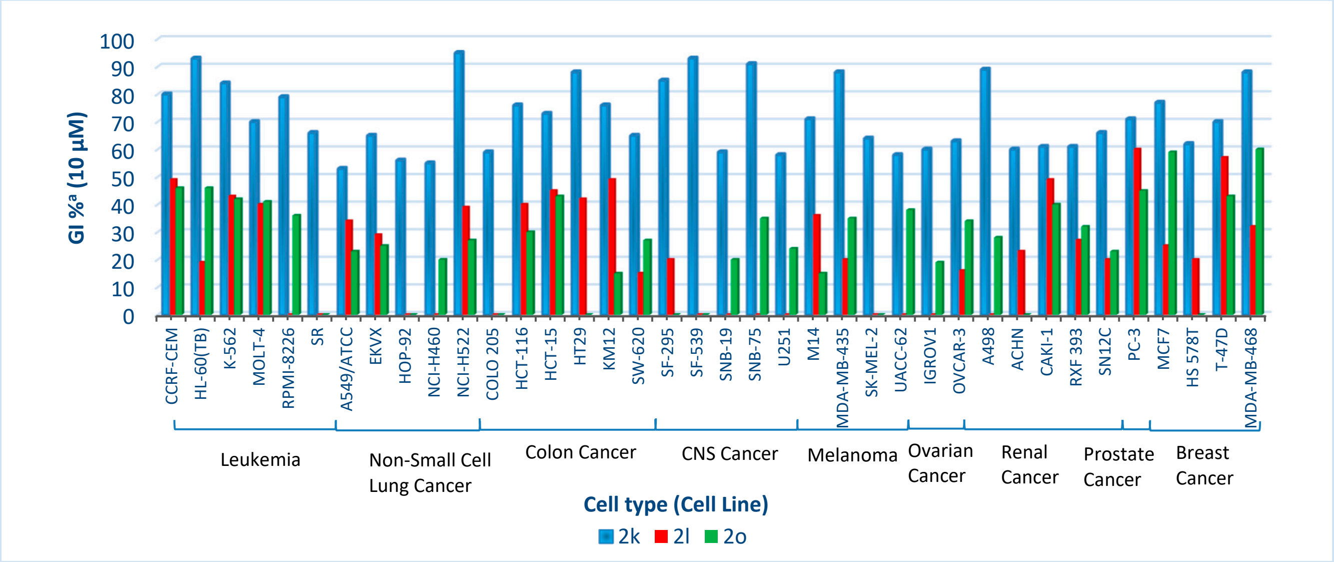

Figure S1. Results of the *in vitro* human cancer cell growth inhibition for selected compounds **2k, 2l** and **2o**.

Docking FTase

Compound 1l

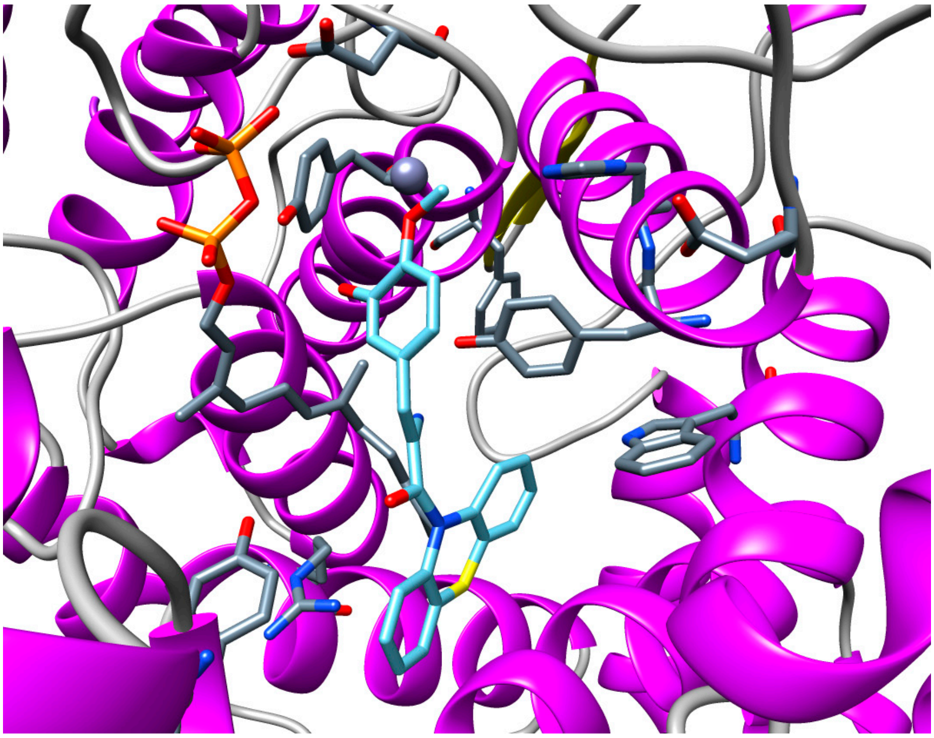

Compound 3a

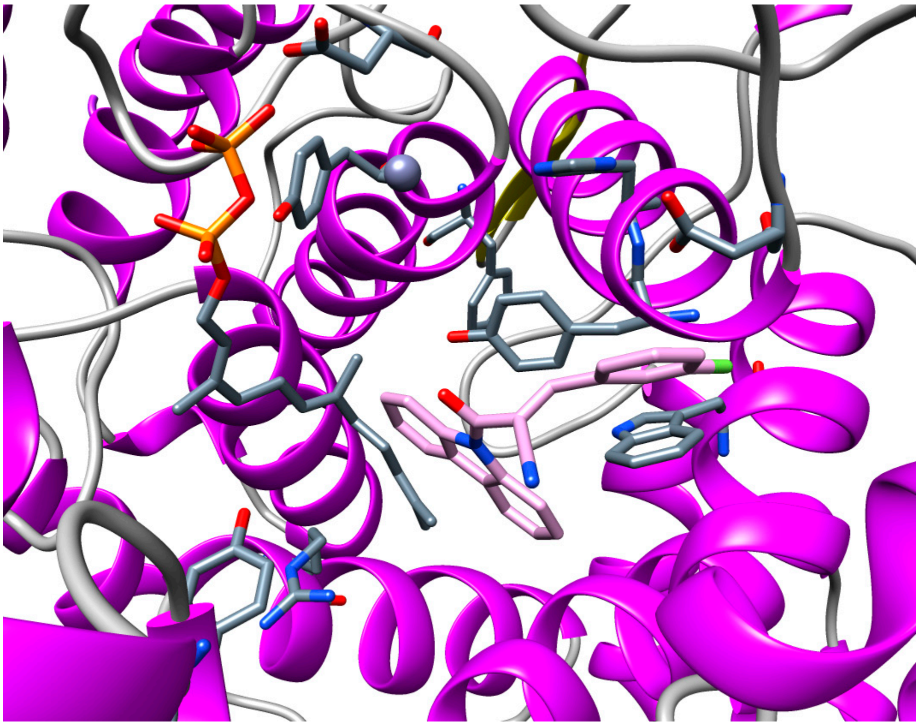

Compound 3b

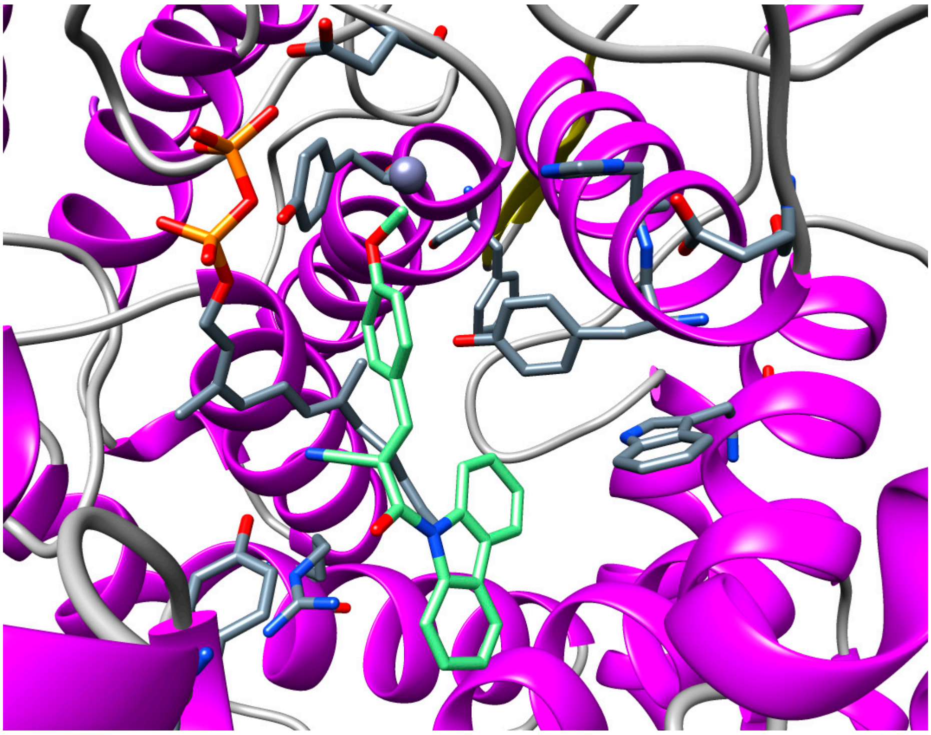

Compound 3d

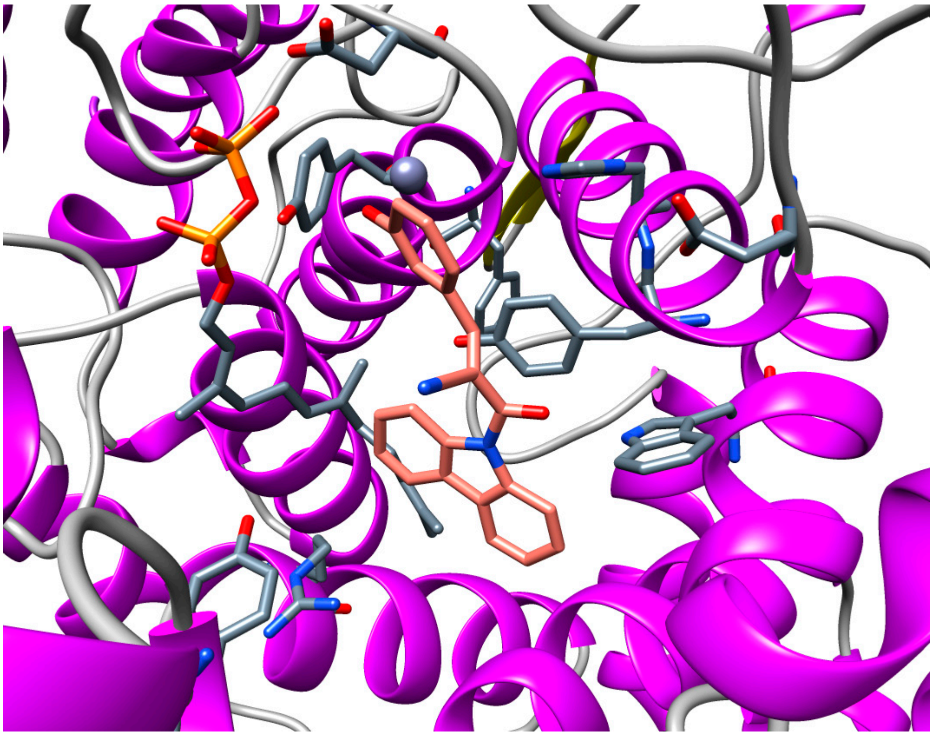

Compound 3e

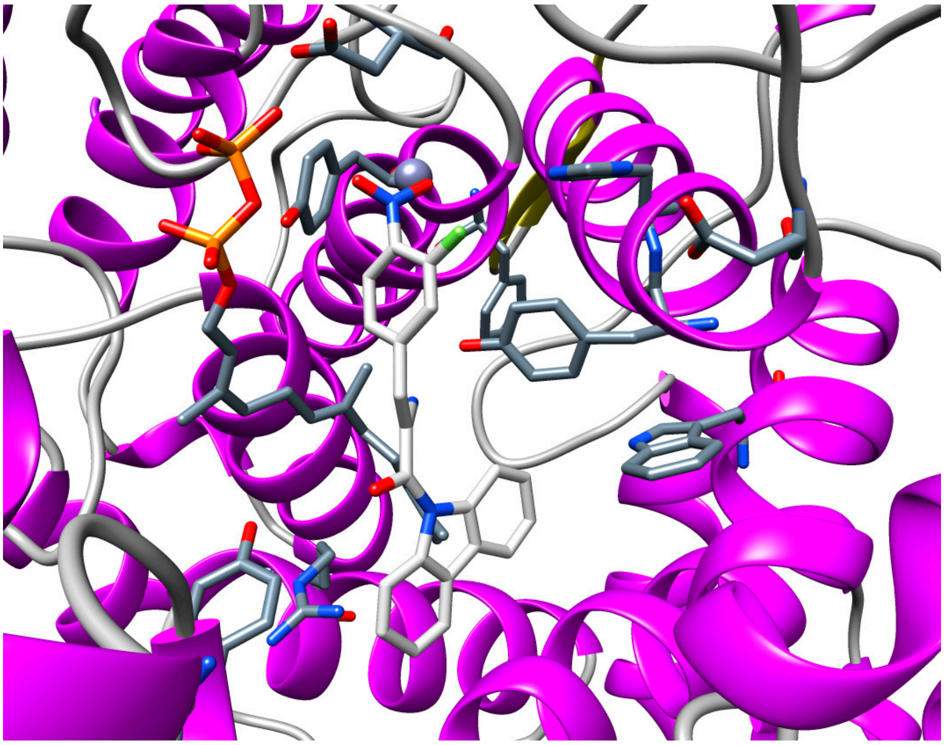

Compound 3i

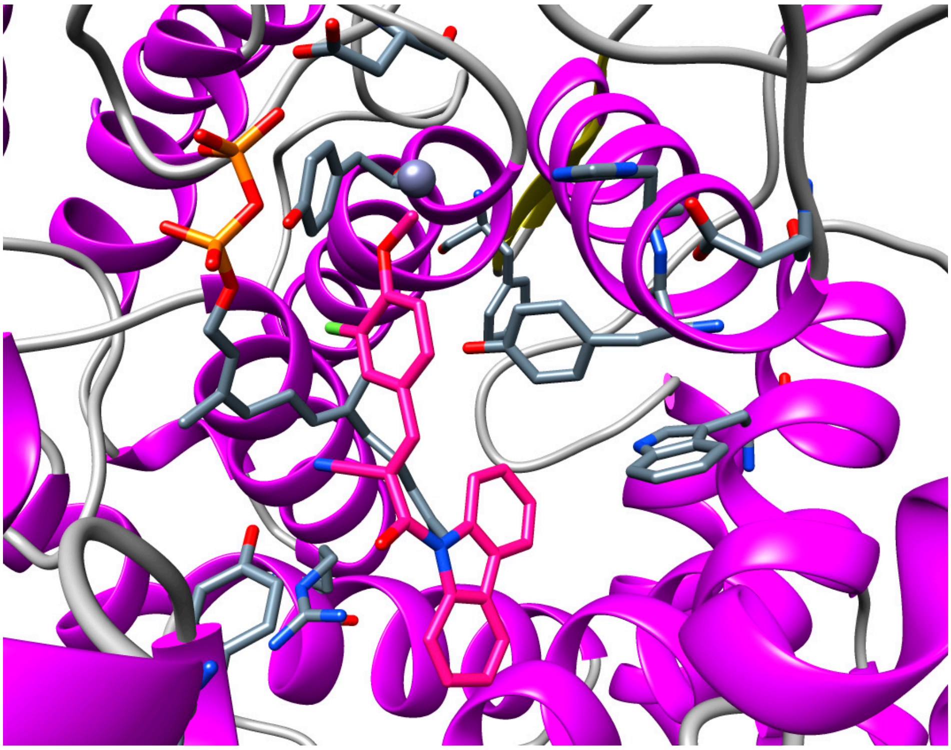

Compound 3j

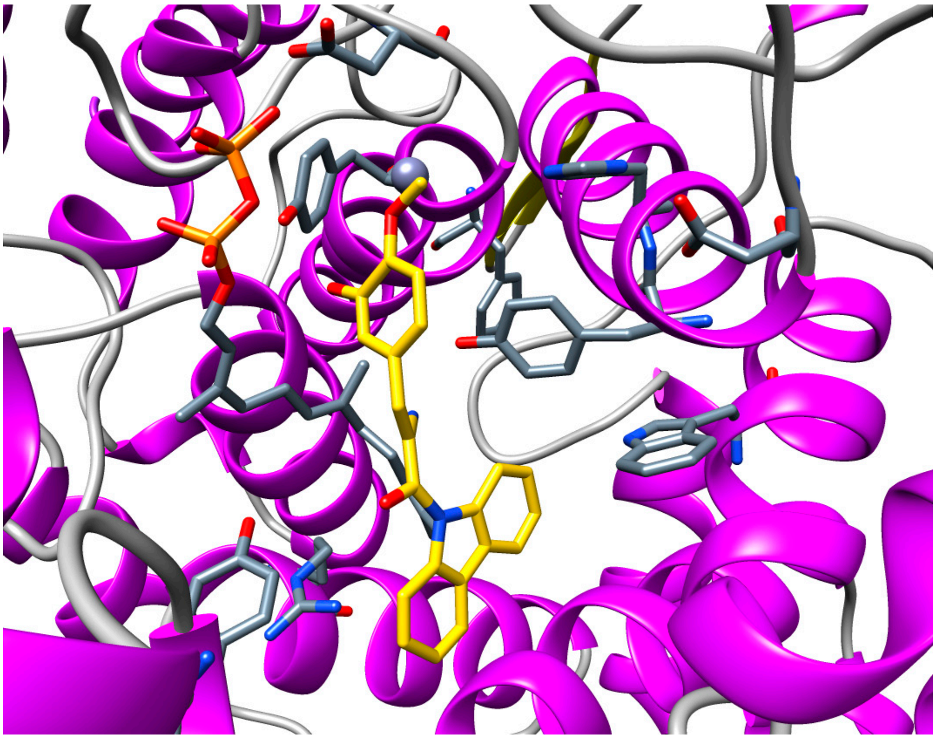

Compound 3l

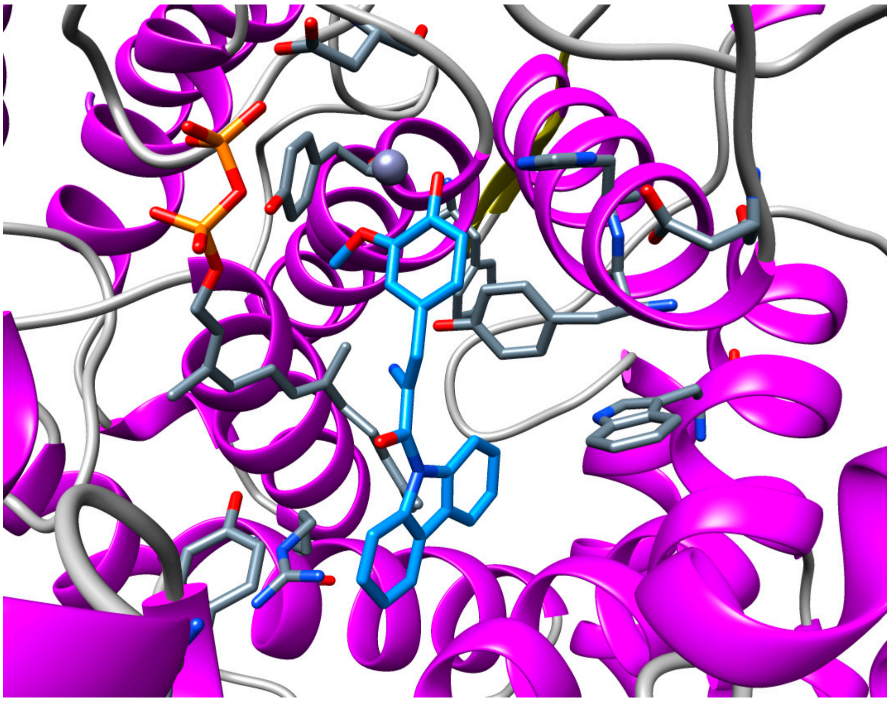

Phenstatin

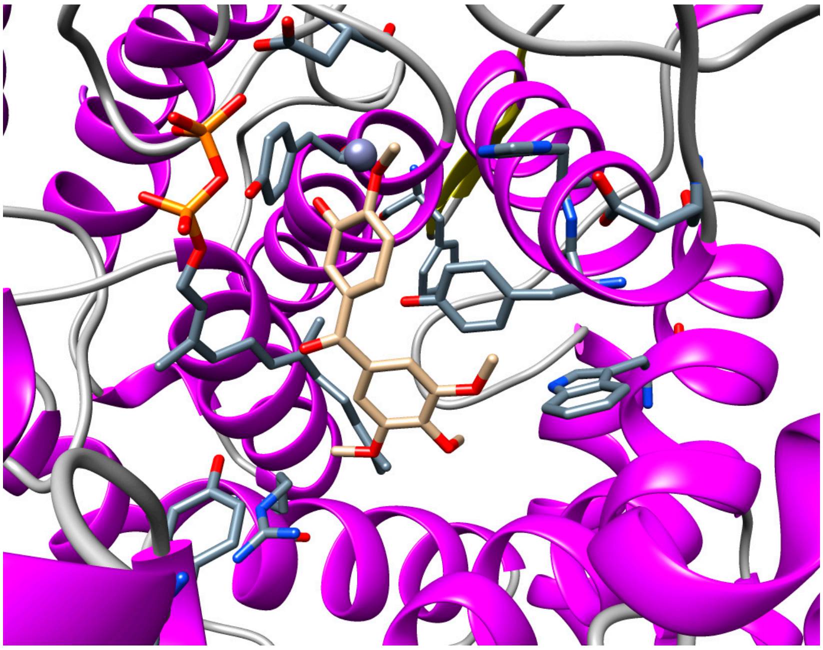

Docking tubulin (colchicine binding site)

Compound 1l (40% of the solutions)

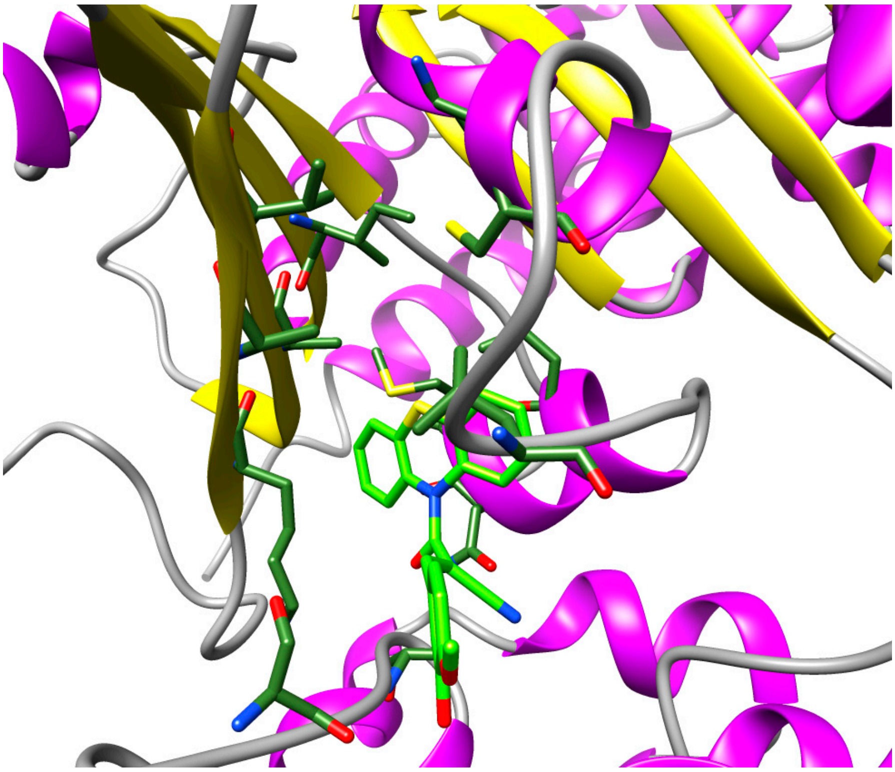

Compound 1l (60% of the solutions)

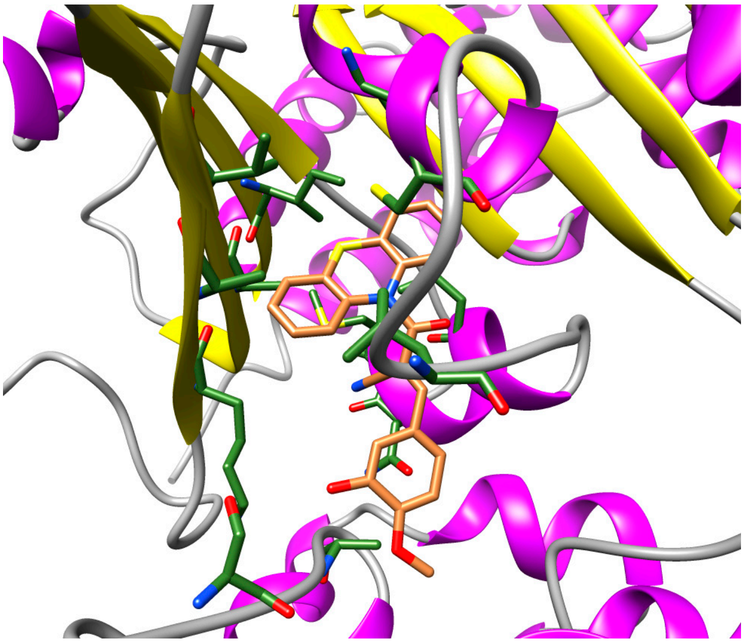

Compound 3a

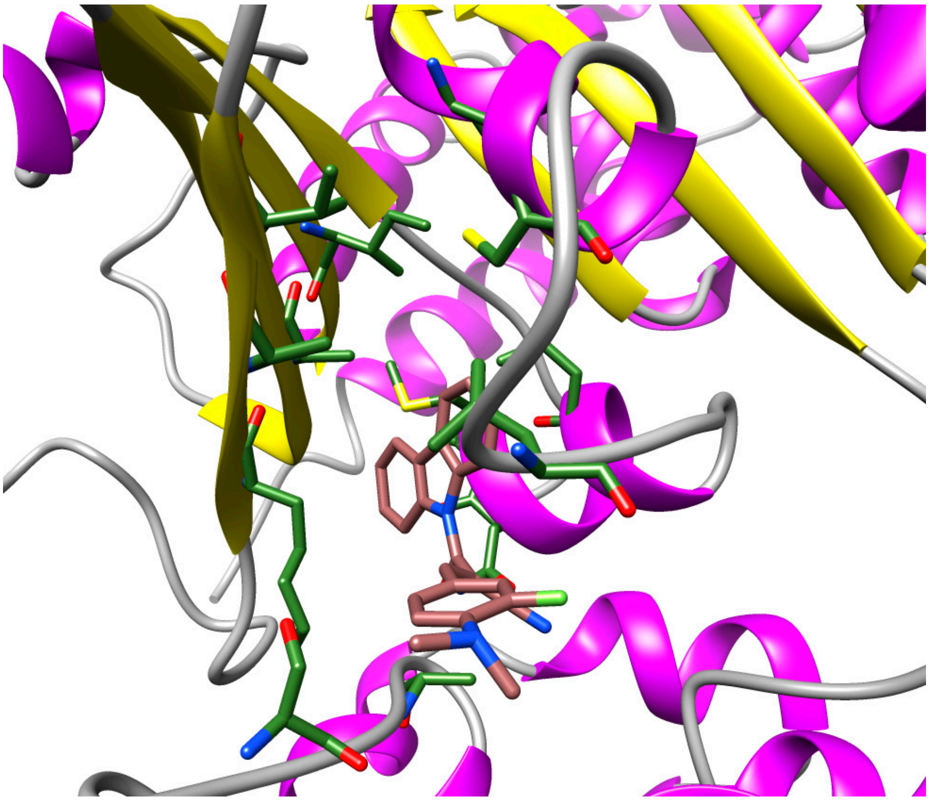

Compound 3b

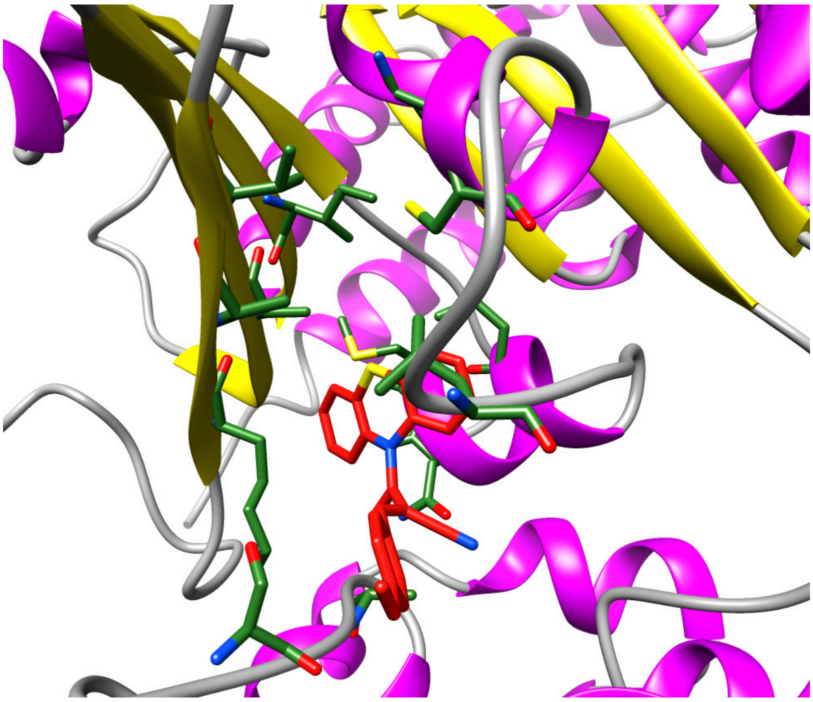

Compound 3d

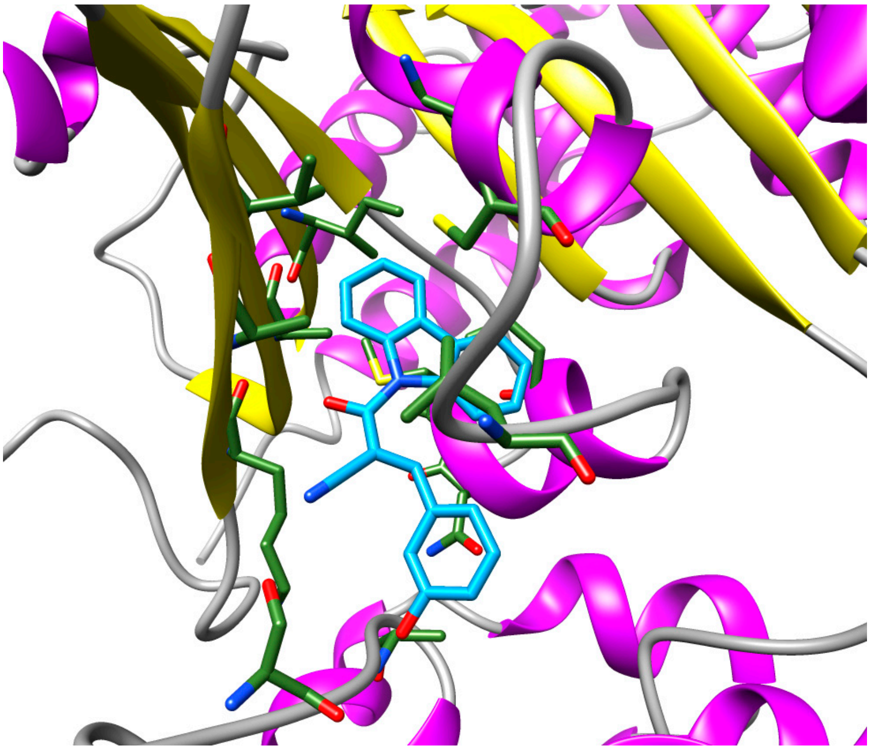

Compound 3e

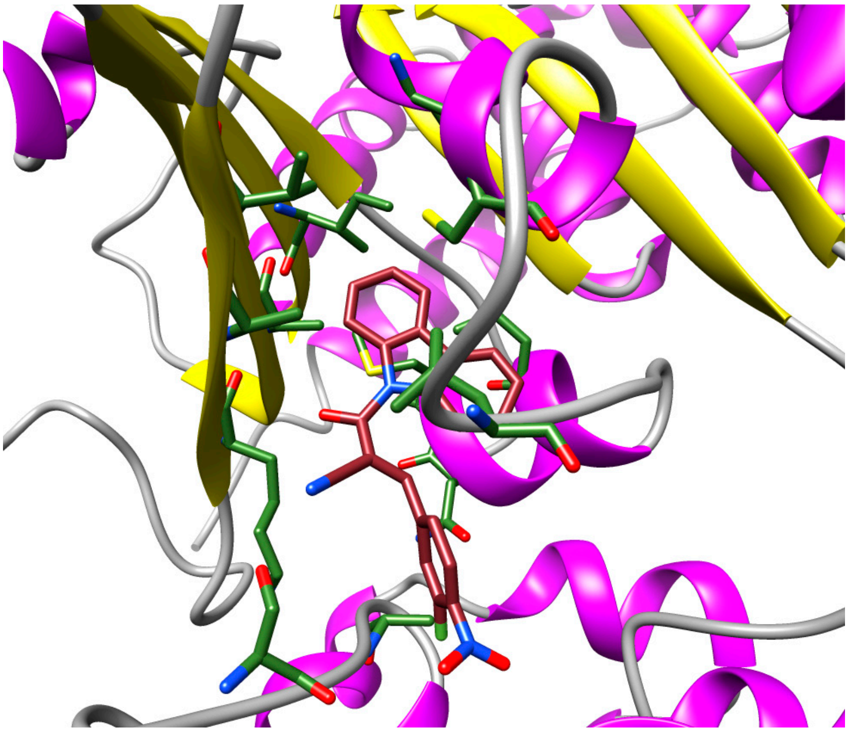

Compound 3i

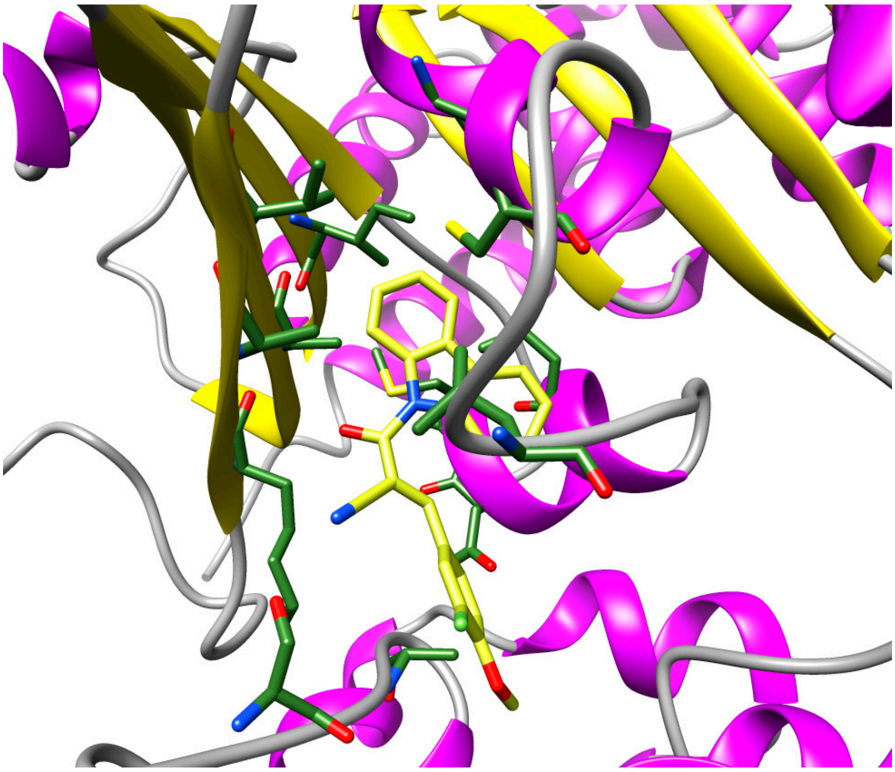

Compound 3j

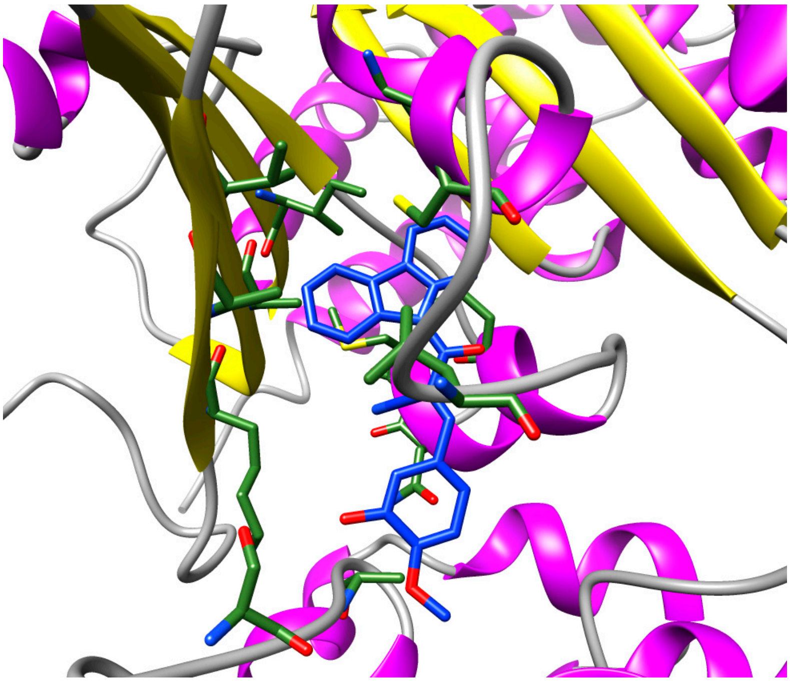

Compound 3l

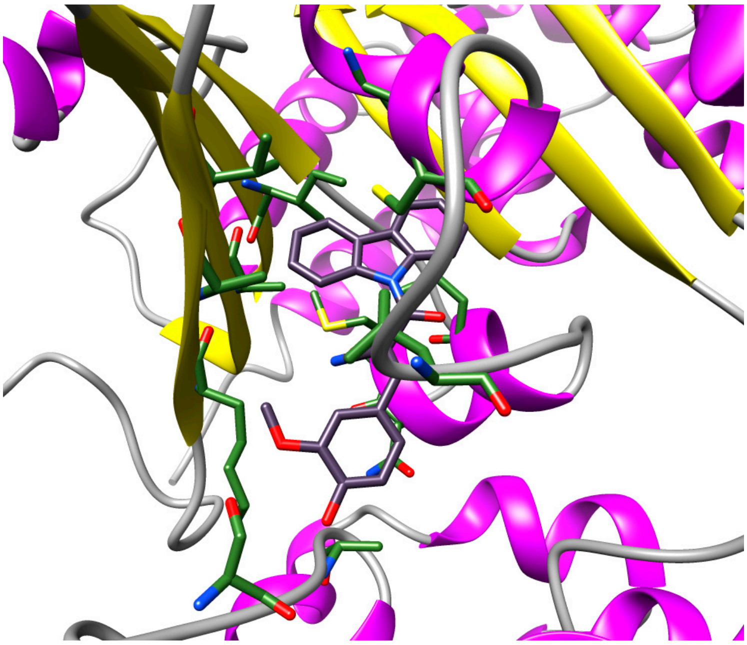

Phenstatin

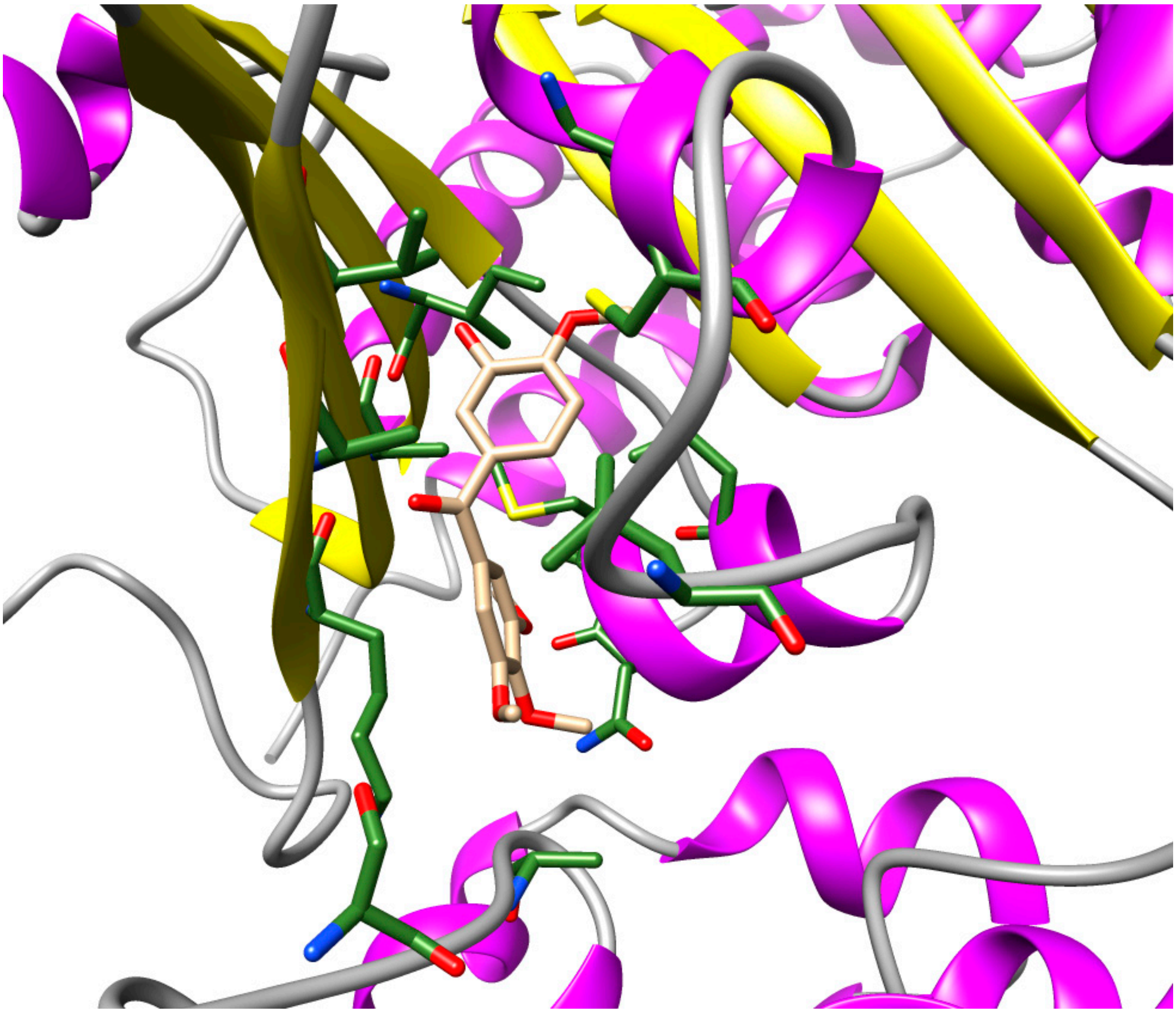

Supplement: Supplementary file 1 [file pharmaceuticals-16-00888-s001.zip › pharmaceuticals-2433650-supplementary.pdf]
